# Integrated Multiomics Analyses of the Molecular Landscape of Sarcopenia in Alcohol‐Related Liver Disease

**DOI:** 10.1002/jcsm.13818

**Published:** 2025-04-30

**Authors:** Nicole Welch, Pugazhendhi Kannan, Saurabh Mishra, Annette Bellar, Vandana Agrawal, Grahame Kidd, Emily Benson, Ryan Musich, Raya Tabbalat, Ling Li, J. Mark Brown, Belinda Willard, Karyn A. Esser, Laura E. Nagy, Srinivasan Dasarathy

**Affiliations:** ^1^ Departments of Gastroenterology and Hepatology Cleveland Clinic, Lerner Research Institute Cleveland Ohio USA; ^2^ Departments of Inflammation and Immunity Cleveland Clinic, Lerner Research Institute Cleveland Ohio USA; ^3^ 3D EM Ultrastructural Imaging and Computation Core Cleveland Clinic, Lerner Research Institute Cleveland Ohio USA; ^4^ Departments of Proteomics Core Cleveland Clinic, Lerner Research Institute Cleveland Ohio USA; ^5^ Departments of Cancer Biology Cleveland Clinic, Lerner Research Institute Cleveland Ohio USA; ^6^ Department of Physiology and Aging University of Florida Gainesville Florida USA

**Keywords:** alcohol‐related liver disease, hypoxia‐inducible factor‐1‐alpha, mitochondrial oxidative dysfunction, protein acetylation, redox ratio, sarcopenia, senescence, sirtuins

## Abstract

**Background:**

Skeletal muscle is a major target for ethanol‐induced perturbations, leading to sarcopenia in alcohol‐related liver disease (ALD). The complex interactions and pathways involved in adaptive and maladaptive responses to ethanol in skeletal muscle are not well understood. Unlike hypothesis‐driven experiments, an integrated multiomics‐experimental validation approach provides a comprehensive view of these interactions.

**Methods:**

We performed multiomics analyses with experimental validation to identify novel regulatory mechanisms of sarcopenia in ALD. Studies were done in a comprehensive array of models including ethanol‐treated (ET) murine and human‐induced pluripotent stem cell–derived myotubes (hiPSCm), skeletal muscle from a mouse model of ALD (mALD) and human patients with alcohol‐related cirrhosis and controls. We generated 13 untargeted datasets, including chromatin accessibility (assay for transposase accessible chromatin), RNA sequencing, proteomics, phosphoproteomics, acetylomics and metabolomics, and conducted integrated multiomics analyses using UpSet plots and feature extraction. Key findings were validated using immunoblots, redox measurements (NAD^+^/NADH ratio), imaging and senescence‐associated molecular phenotype (SAMP) assays. Mechanistic studies included mitochondrial‐targeted *Lactobacillus brevis* NADH oxidase (MitoLbNOX) to increase redox ratio and MitoTempo as a mitochondrial free radical scavenger.

**Results:**

Multiomics analyses revealed enrichment in mitochondrial oxidative function, protein synthesis and senescence pathways consistent with the known effects of hypoxia‐inducible factor 1α (HIF1α) during normoxia. Across preclinical and clinical models, HIF1α targets (*n* = 32 genes) and signalling genes (*n* > 100 genes) (*n* = 3 ATACseq, *n* = 65 phosphoproteomics, *n* = 10 acetylomics, *n* = 6 C2C12 proteomics, *n* = 106 C2C12 RNAseq, *n* = 64 hiPSC RNAseq, *n* = 30 hiPSC proteomics, *n* = 3 mouse proteomics, *n* = 25 mouse RNAseq, *n* = 8 human RNAseq, *n* = 3 human proteomics) were increased. Stabilization of HIF1α (C2C12, 6hEtOH 0.24 ± 0.09; *p* = 0.043; mALD 0.32 ± 0.074; *p* = 0.005; data shown as mean difference ± standard error mean) was accompanied by enrichment in the early transient and late change clusters, −log(*p*‐value) = 1.5–3.8, of the HIF1α signalling pathway. Redox ratio was reduced in ET myotubes (C2C12: 15512 ± 872.1, *p* < 0.001) and mALD muscle, with decreased expression of electron transport chain components (CI–V, *p* < 0.05) and Sirt3 (C2C12: 0.067 ± 0.023, *p* = 0.025; mALD: 0.41 ± 0.12, *p* = 0.013). Acetylation of mitochondrial proteins was increased in both models (C2C12: 107364 ± 4558, *p* = 0.03; mALD: 40036 ± 18 987, *p* = 0.049). Ethanol‐induced SAMP was observed across models (P16: C2C12: 0.2845 ± 0.1145, *p* < 0.05; hiPSCm: 0.2591, *p* = 0.041). MitoLbNOX treatment reversed redox imbalance, HIF1α stabilization, global acetylation and myostatin expression (*p* < 0.05).

**Conclusions:**

An integrated multiomics approach, combined with experimental validation, identifies HIF1α stabilization and accelerated post‐mitotic senescence as novel mechanisms of sarcopenia in ALD. These findings show the complex molecular interactions leading to mitochondrial dysfunction and progressive sarcopenia in ALD.

## 1 Introduction

Sarcopenia or skeletal muscle loss is recognized in a number of chronic diseases and is most severe and progresses in patients with alcohol‐related liver disease (ALD) even after abstinence [1]. Sarcopenia contributes to higher mortality, more frequent and longer duration of hospitalization and poor quality of life in ALD with no effective therapies, primarily because of a limited understanding of the underlying mechanisms [2, 3]. Mechanistic studies show dysregulated protein homeostasis (proteostasis) with decreased protein synthesis and increased autophagy in sarcopenia of ALD [2, 4, 5, 6], processes that are regulated by cellular ATP levels [7]. Consistently, mitochondrial oxidative dysfunction with defects in components/assembly of the electron transport chain (ETC) have been reported with ethanol exposure and in ALD [8]. Targeted, hypotheses‐driven approaches do not, however, allow for simultaneous evaluation of multiple cellular responses that occur during both adaptive and maladaptive responses [5, 9, 10]. Less is known about the interactions of these biological processes, their functional consequences and initiation of multiple transcriptional, translational and posttranslational modifications and their effect on cellular responses. Complex interactions between different biological modalities (chromatin, RNA, protein, metabolites) and regulatory molecules are evaluated using orthogonal designs where multiomics studies include temporal changes in the expression of molecules with experimental validation [9, 11]. Such orthogonal approaches can identify the global landscape of cellular responses to stressors in metabolically active organs like skeletal muscle. A number of standard pipelines and pathway analyses use both shared and unique algorithms to analyse unbiased datasets making replicating and interpreting conclusions challenging [12]. Additionally, a number of shared molecules across pathways can result in interactions/intersections across cellular responses [9, 11, 12]. In many instances, the consequence of alterations in expression and responses to these molecules is also context dependent. Therefore, such unbiased data analyses using simultaneous analytical tools in specific organs/cellular systems provide novel biological interpretations and help identify novel therapeutic targets.

Ethanol‐induced tissue injury to the skeletal muscle is well recognized [4, 5, 8], but the global molecular responses and their potential impact on mediating sarcopenia are not known. We used our previously reported method of vertical integration across molecular layers including chromosomal access that determines transcription using assay for transpose accessible chromatin sequencing (ATACseq), bulk RNA sequencing (RNAseq), proteomics, phosphoproteomics and acetylomics [9]. We horizontally integrated these unbiased data across myotubes and skeletal muscle from mice with ALD and human patients. Temporal clustering, correlations and interactions of our datasets allowed us to demonstrate that ethanol causes perturbations in solute transporters, extracellular matrix molecules, hypoxia‐inducible factor‐1α (HIF1α) signalling/target genes, mitochondrial ETC components, NAD and sirtuin pathways with post‐mitotic senescence. These data provide mechanistic explanations for persistent and progressive sarcopenia in ALD and clinical observations of limited response to anabolic interventions.

## 2 Materials and Methods

All chemicals and reagents were obtained from Sigma‐Aldrich (St. Louis, MO), and antibodies were obtained from Cell Signaling Technology (Danvers, MA) unless specified. Details of these are described in Table S1. Tables S2 and S3 are also provided.

Studies were done in a comprehensive array of models including differentiated murine C2C12 myotubes, human‐induced pluripotent stem cell–derived myotubes (hiPSCm), mouse model of alcohol‐associated liver disease (mALD) and skeletal muscle from human patients with cirrhosis (CIR) and controls (CTL) as described earlier [5, 8, 9, 11, 13, 14, 15]. All animal studies were approved by the Institutional Animal Care Use Committee (0000‐2610). All human studies were performed after obtaining a written informed consent and approved by the Cleveland Clinic Institutional Review Board (IRB 14‐1287).

Details of the methods used, analytical approaches and statistical methods have been previously reported [4, 5, 8, 9, 11, 13, 14, 15] and are described in the Supporting Information.

## 3 Results

### 3.1 Integrated Multiomics Analyses Identify Shared and Unique Molecules Across Molecular Layers and Datasets

**FIGURE 1:**
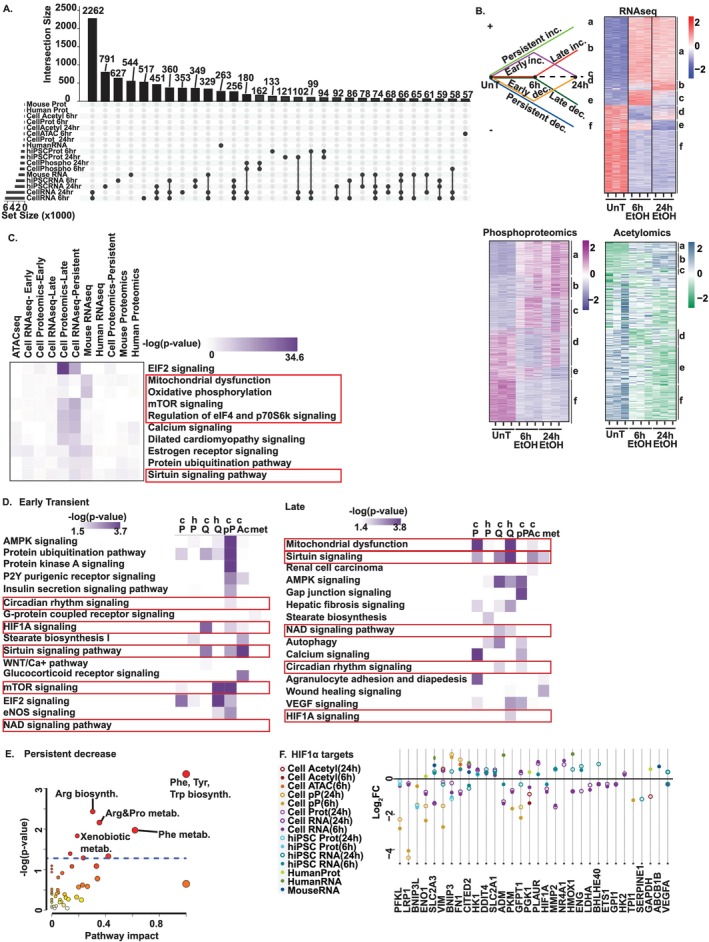
Differentially expressed molecules show conserved regulators of cellular function with ethanol. Assay for transposase accessible chromatin sequencing (ATACseq), RNA sequencing (RNAseq; Q), proteomics (P), phosphoproteomics (Pp), acetylomics (Ac) and metabolomics was generated in differentiated murine C2C12 (c) and human‐induced pluripotent stem cell–derived (h) myotubes treated without (UnT)/with 100 mM ethanol (EtOH) for 6 and 24 h, gastrocnemius muscle from mouse model of alcohol‐associated liver disease and humans with alcohol‐associated cirrhosis/healthy controls. Differentially expressed molecules (DEMs) were identified. (A) UpSet plot: Y‐axis is the number of shared molecules between datasets. Dots represent dataset(s) containing DEMs. (B) Line diagram and heatmaps showing temporal clusters of responses in murine myotubes—early transient: change only at 6 h EtOH; late: change only at 24 h EtOH; persistent: change at both 6 h EtOH and 24 h EtOH versus UnT; and pseudosilent: change only at 24 h EtOH versus 6 h EtOH. (C) Functional enrichment using QIAGEN Ingenuity Pathway Analysis. (D) Functional enrichment within early transient and late change temporal clusters. (E) Pathway impact dot‐plot of untargeted metabolomics at 24 h EtOH in myotubes. Higher impact values indicate the relative significance of a pathway. Circle size: impact; circle colour: increasing significance from white to red. (F) Integrated hierarchical scatterplots of intersecting DEMs that are HIF1α targets. The dot colour corresponds to the dataset. Significance cutoffs: for ATACseq (*p* < 0.005); RNAseq myotubes (*p*‐adjusted < 0.05); all others *p* < 0.05. Red box: Critical components experimentally validated.

UpSet plots of 17 multiomics datasets revealed fewer shared differentially expressed molecules (DEMs) as the number of intersection sets increased (Figure 1A and Table S4). The greatest number of shared DEMs occurred within the same molecular layer across models, suggesting concordant transcriptional and translational responses. However, this was not seen between different molecular layers, as chromosomal accessibility does not always result in transcription, nor are all transcripts translated [16]. Fewer shared DEMs across in vivo datasets than in vitro as reported earlier in other models [9] may result from the contribution of multiple organ interactions and adaptive responses to ethanol disposal and metabolism. Molecular responses following ethanol exposure occurred in temporal patters: early transient (temporary increase/decrease), late (delayed increase/decrease), persistent (early and sustained increase/decrease) and pseudosilent (increase/decrease between early and late timepoints, but unchanged from baseline at both) changes (Figure 1B). Pathway analyses showed that mitochondrial dysfunction/oxidative phosphorylation, mTORC1 and sirtuin signalling pathways were most enriched in response to ethanol exposure across models (Figure 1C, Tables S5 and S6 and Figures S1–S10). Early transient and late change clusters both showed enrichment of circadian rhythm, sirtuin, HIF1α and NAD signalling, whereas mTOR signalling was enriched only in the early transient cluster. Mitochondrial dysfunction was enriched in both the late change and persistent change clusters (Figures 1D and S11). Persistent changes could be a consequence of adaptive responses, whereas a late increase could be a maladaptive response, and early transient responses might reflect an adaptive exhaustion, as has been noted in other conditions [17].

Ethanol exposure is known to alter metabolites in circulation and the liver [18], but skeletal muscle metabolites in ALD are not known. We therefore identified differentially expressed metabolites using mass spectrometry. Pathways enriched in early transient cluster of metabolites that are decreased included ‘steroid hormone biosynthesis’ and ‘taurine and hypotaurine metabolism’ and suggest short‐term reductions in hormone regulation and detoxification. Late change cluster metabolites that were decreased included enriched pathways of ‘histidine’ and ‘beta‐alanine metabolism’, whereas persistently decreased metabolites showed enrichment in xenobiotic, amino acid and protein synthesis pathways and indicated reduced amino acid availability. In contrast, pathways like ‘Drug metabolism–cytochrome P450’ and ‘Glyoxylate and dicarboxylate metabolism’ in the late change cluster metabolites that were increased suggest ongoing detoxification. These observations show the complex temporal dynamics of metabolite responses and help identify potential therapeutic targets for sarcopenia in ALD (Figures 1E and S8).

Recent evidence suggests that the transcription factor, HIF1α, regulates many of the enriched pathways in ethanol‐exposed muscle [15]. Consistently, a number of DEMs in HIF1α signalling and target genes were identified on integrated hierarchical scatterplots (IHSP) that included phosphofructokinase, low‐density lipoprotein receptor‐related protein 1 and BCL2 interacting protein‐3‐like in addition to a number of other canonical genes (Figure 1F). Similar IHSP for mitochondrial‐targeted and senescence‐associated genes showed a number of molecules that are either upregulated or downregulated in response to ethanol exposure (Figure S12). Consistent with our recent findings that HIF1α inhibits mitochondrial oxidative function [15], we then investigated skeletal muscle mitochondrial structure and function in ALD.

### 3.2 Ethanol Induces Functional and Structural Perturbations in Mitochondria in Myotubes and Muscle Tissue

**FIGURE 2:**
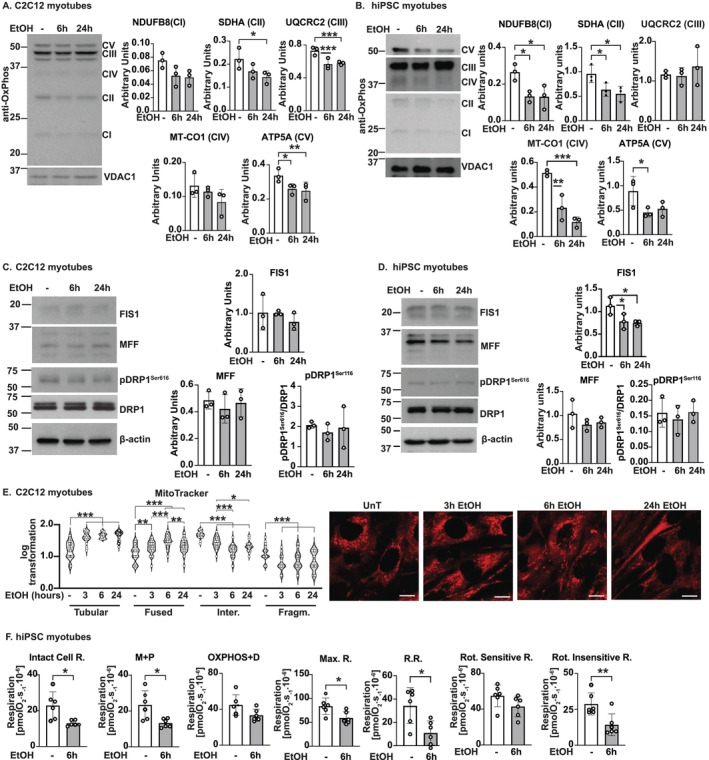
Ethanol‐induced mitochondrial structural and functional perturbations in myotubes. Differentiated C2C12 or human‐induced pluripotent stem cell–derived myotubes were treated without (UnT)/with 3, 6 or 24 h 100 mM ethanol (EtOH). Representative immunoblots and densitometries of (A,B) components of each electron transport chain complex; (C,D) mitochondrial structural proteins dynamin‐related protein 1 (DRP1), phosphorylated‐DRP1^Ser616^, fission, mitochondrial 1 (FIS1) and mitochondrial fission factor (MFF); (E) representative confocal photomicrographs and violin plots. Orange: MitoTracker Orange stained mitochondria. *n* = 30 measurements each from *n* = 3 biological replicates per treatment. Scale bar: 5 mm. (F) Mitochondrial oxygen consumption by high‐resolution respirometry in response to substrate–uncoupler–inhibitor titration. Intact cell respiration, oxidative phosphorylation (OXPHOS) + ADP(D), maximum respiration (Max. R.), reserve respiratory capacity (R.R.), rotenone (Rot.)‐sensitive and ‐insensitive respiration. Data mean ± SD from *n* ≥ 3. 2‐groups: Student's *t*‐test. > 2‐groups: One‐way ANOVA and uncorrected Fisher's LSD. *p < 0.05; **p < 0.01; ***p < 0.001. Loading control shown: (B) VDAC1: OxPhos CV/CII/CI. (C) β‐actin: MFF; (D) β‐actin: FIS1/MFF.

Expression of critical components of ETC Complexes II, III and V was less in ethanol‐treated murine and hiPSC myotubes, whereas that of Complexes I and IV was also lower in hiPSC myotubes (Figure 2A,B). FIS1 expression was lower in hiPSC myotubes, but other regulators of mitochondrial biogenesis showed no changes (Figures 2C,D and S13A,B). The altered mitochondrial structure has been reported to accompany oxidative dysfunction. We found that mitochondria in ethanol‐treated myotubes display more tubular and fused morphology than fragmented and intermediate forms compared with untreated myotubes (Figure 2E). We then assessed whether the reduction in ETC component expression and morphological changes altered mitochondrial oxidative function in situ. Consistent with previous reports of reduced Complex I function in the ETC with ethanol exposure and in mALD, we observed decreased intact cell respiration, maximum respiration, reserve respiratory capacity and rotenone insensitive (Complex II) function in ethanol‐treated hiPSCm (Figures 2F and S13C).

**FIGURE 3:**
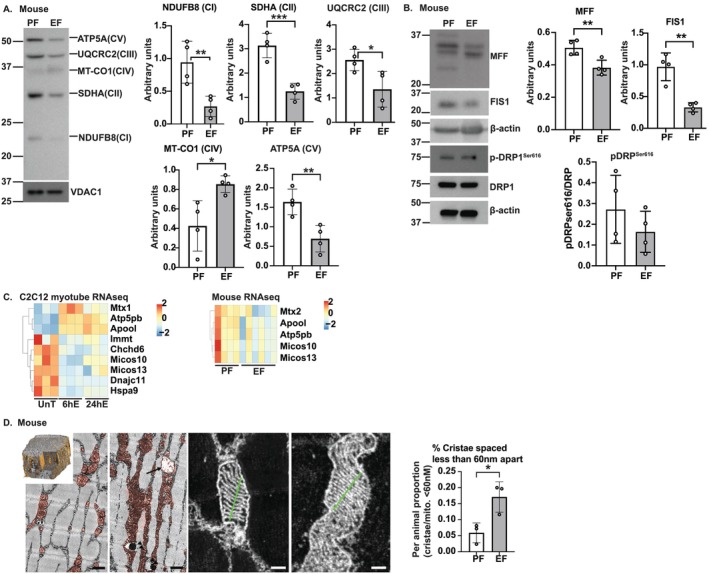
Mitochondrial perturbations in skeletal muscle in mice with alcohol‐associated liver disease. Studies were done in gastrocnemius muscle from pair‐fed (PF) or mice‐fed ethanol (EF) and C2C12 myotubes without/with 100 mM ethanol (EtOH) treatment for 6 and 24 h. (A,B) Representative immunoblots and densitometries of mouse skeletal muscle: (A) Components of each electron transport chain complex. (B) Mitochondrial structural proteins dynamin‐related protein 1 (DRP1), phosphorylated‐DRP1^Ser616^, fission, mitochondrial 1 (FIS1) and mitochondrial fission factor (MFF). (C) Heatmaps of differentially expressed molecules in RNAseq from myotubes and mouse skeletal muscle (REACTOME cristae formation geneset). (D) Cristae spacing using three‐dimensional electron microscopy (3D EM) of murine gastrocnemius muscle (inset: reconstructed muscle fibre, orange: mitochondria). Data mean ± SD from *n* ≥ 3 per group. Scale bars 1 μm (black), 0.5 μm (white). 2‐groups: Student's *t*‐test. *p < 0.05; **p < 0.01; ***p < 0.001. β‐actin shown from FIS1, p‐DRP1^Ser616^.

In vivo relevance of these cellular data was noted in studies in gastrocnemius muscle from mALD that showed lower expression of critical components of Complexes I, II, III and V (Figure 3A). Mitochondrial biogenesis markers showed that expression of two molecules, known to be involved in mitochondrial fission, Fis1 homologue (FIS1) and mitochondrial fission factor (MFF), was less in mALD muscle (Figure 3B and S13D). We then evaluated whether markers of cristae formation that regulate the efficiency of oxidative phosphorylation were altered. Transcriptomics showed that a decrease in Micos10 and Micos13, which maintain cristae junction, was noted across models (Figure 3C). Consistently, there was a higher proportion of mALD skeletal muscle mitochondria with narrow cristae on 3D electron microscopy than pair‐fed controls (Figure 3D). We and others have shown skeletal muscle mitochondrial oxidative dysfunction in response to ethanol exposure and ALD [5, 8, 19]. In the present studies, we show translational relevance in hiPSCm that shows mitochondrial oxidative dysfunction in response to ethanol treatment.

### 3.3 Ethanol Lowers Redox Ratio and Sirtuin Expression in Myotubes and Muscle Tissue

**FIGURE 4:**
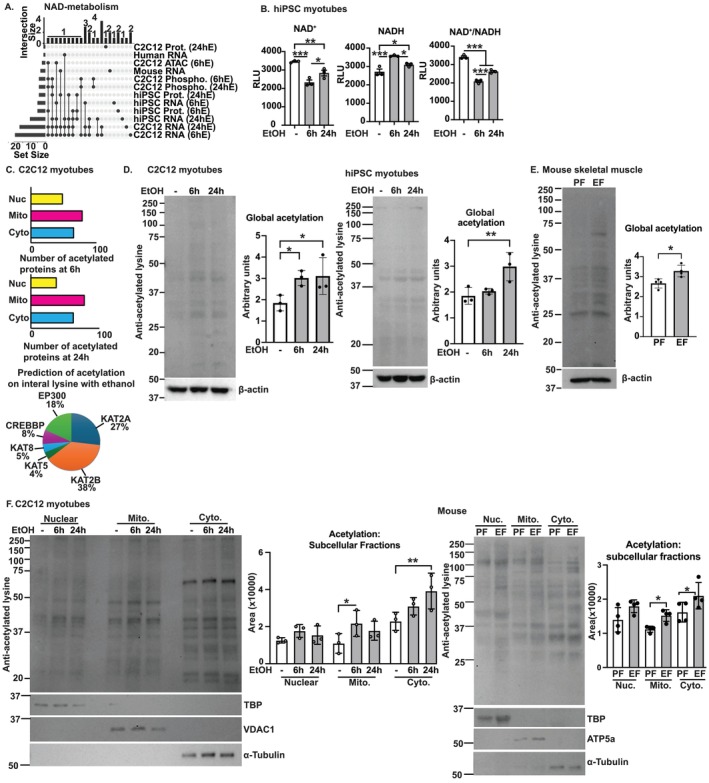
Ethanol‐induced lower redox ratio causes global acetylation and less sirtuin expression in myotubes and skeletal muscle. Studies done in differentiated murine and human‐induced pluripotent stem cell–derived (hiPSC) myotubes and skeletal muscle from mice (ethanol‐ and pair‐fed [EF and PF]) and humans without/with cirrhosis. (A) UpSet plot of adenine dinucleotide pathway genes. Y‐axis: number of shared molecules between datasets. Dots: dataset(s) containing shared/unique differentially expressed molecules. (B) Fluorescence‐based quantification of NAD^+^, NADH and the NAD^+^/NADH ratio in hiPSC myotubes. (C) Subcellular acetylomics in myotubes. (D,E) Representative immunoblots and densitometry of global protein acetylation. (F) Representative immunoblots and densitometry of global acetylation in mitochondrial, cytosolic and nuclear subcellular fractions. Significance cutoffs: ATACseq (*p* < 0.005); myotube RNAseq (*p*‐adjusted < 0.05); all others (*p* < 0.05). All experimental data mean ± SD from *n* ≥ 3 using one‐way ANOVA with uncorrected Fisher's LSD. *p < 0.05; **p < 0.01; ***p < 0.001.

Impaired mitochondrial oxidative function contributes to a lower redox ratio by altering adenine dinucleotide oxidation/reduction [14]. Our bioinformatics data show the enrichment of genes in the NAD metabolism pathway in our models (Figure 4A). Experimentally, less NAD^+^, more NADH and lower NAD^+^/NADH ratio are noted in ethanol‐treated hiPSC myotubes (Figure 4B), which is consistent with our previous report in murine myotubes [8]. In the untargeted acetylomics analyses, the number of mitochondrial‐targeted proteins is higher than those in nuclear and cytoplasmic proteins (Figure 4C). Consistent with our previous data that redox ratio regulates lysine acetylation, we note that global acetylation of proteins is higher in murine myotubes and muscle from mALD (Figure 4D,E). Subcellular fractions show significant increases in global acetylation in mitochondria and cytosol in murine myotubes and muscle from mALD (Figure 4F). We then identified predicted sites for lysine acetyltransferases that show a preponderance of the acetylated residues are targets of KAT2(A/B) followed by EP300 (CBP and p300) [20]. Our data show increased muscle protein acetylation in ALD, which may be responsible for some of the functional perturbations because acetylation impairs protein function [14]. We then determined the effect of ALD on lysine deacetylases, which also determined the protein acetylation status.

### 3.4 Ethanol Treatment Decreases Sirtuin Expression by Inhibiting NADH Oxidase

**FIGURE 5:**
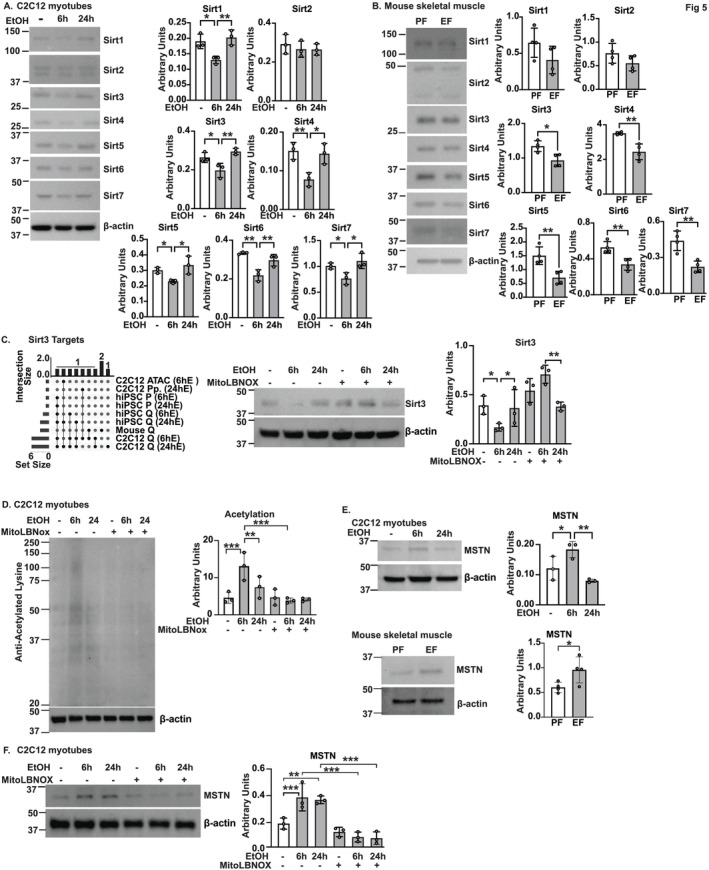
Redox ratio regulates sirtuin expression and acetylation during ethanol treatment. Studies were done in differentiated murine C2C12 myotubes treated without/with 6 or 24 h of 100 mM ethanol (EtOH) and skeletal muscle from pair‐ and ethanol‐fed (PF and EF) mice. Representative immunoblots and densitometries of Sirtuins (Sirt1–7) in (A) myotubes, (B) mouse skeletal muscle; (C) Sirt3 in response to mitochondrial‐directed *Lactobacillus brevis* NADH oxidase (MitoLbNOX) with ethanol treatment in myotubes and UpSet plot of differentially expressed Sirt3 target genes across datasets. Expression of (D) acetylated protein without/with MitoLbNOX in myotubes; (E) myostatin (MSTN) in myotubes and muscle; (F) MSTN in without/with MitoLbNOX. Data mean ± SD from *n* = 3 (myotubes); *n* = 4 (mice)/group. 2‐groups: Student's *t*‐test; > 2‐groups: One‐way ANOVA with uncorrected Fisher's LSD. *p < 0.05; **p < 0.01; ***p < 0.001. Significance: ATACseq (*p* < 0.005); myotube RNAseq (*p*‐adjusted < 0.05); others (*p* < 0.05). β‐actin shown from (A) Sirt1/3/5/7 and (B) Sirt6/7.

Sirtuins are lysine deacetylases that are responsive to redox changes and decrease skeletal muscle senescence. Our bioinformatics analyses showed enrichment in sirtuin signalling and target pathways (Figure 1C,D). Experimentally, we showed that the expression of most known sirtuins (Sirt1,3–7) was less in ethanol‐treated murine, but not in hiPSC, myotubes (Figure 5A and S14). Consistently, expression of Sirt3–7 was less in mALD skeletal muscle (Figure 5B). Bioinformatics analyses showed differential expression of sirtuin target genes across models (Figure 5C). Sirtuins are regulated by redox ratio and MitoLbNOX that increases NADH oxidation and redox ratio, reversed ethanol‐induced reduction in Sirt3 expression (Figure 5C). Consistently, ethanol‐induced global acetylation was also reversed by MitoLbNOX in murine myotubes (Figure 5D). Functional consequences of ethanol‐induced acetylation showed increased acetyl‐p65NFkB in myotubes (Figure S15) with higher expression of myostatin in myotubes and mouse skeletal muscle, which is transcriptionally regulated by p65‐NFkB (Figure 5E). Similar to reduction in acetylation, myostatin expression was also less with MitoLbNOX in ethanol‐treated myotubes (Figure 5F). In addition to the lysine deacetylase function, Sirt3 also improves mitochondrial oxidative function and reduces free radical generation [21]. Sirt3 depletion causes HIF1α stabilization via an increase in reactive oxygen species in cancer cells [22], whereas HIF1α depletion results in increased expression of Sirt3 in skeletal muscle [15]. These data show that the redox‐Sirt3 axis is impaired and results in the upregulation of myostatin, a known contributor to sarcopenia [23].

### 3.5 Ethanol Exposure Results in Normoxic Stabilization of Hypoxia‐Inducible Factor 1α

**FIGURE 6:**
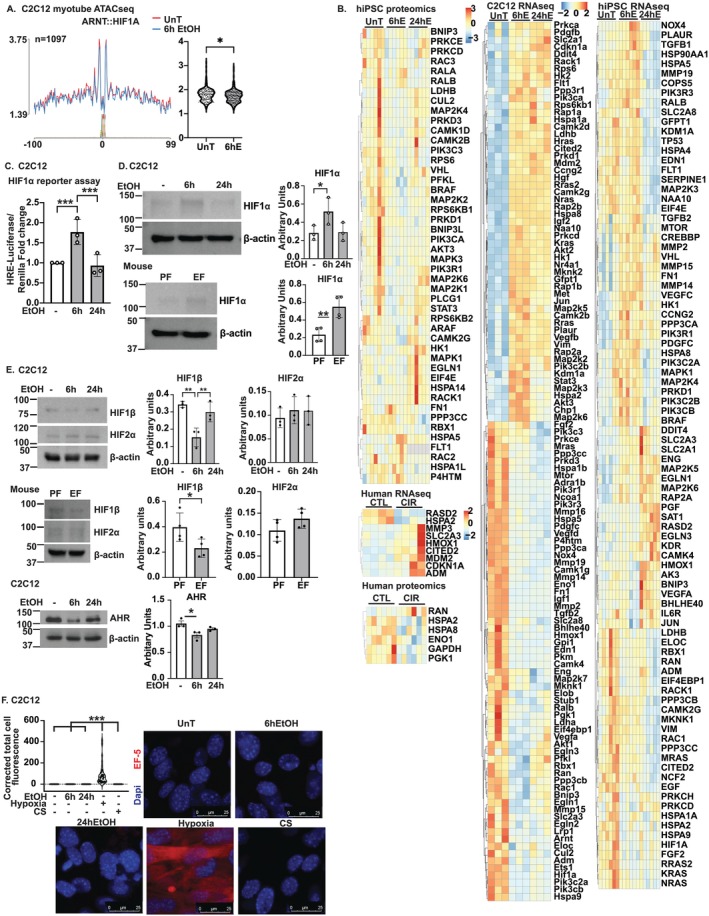
HIF1α transcription factor alterations in skeletal muscle in response to ethanol. Differentiated murine C2C12 or induced human pluripotent stem cell–derived myotubes (hiPSCm) myotubes treated with 6 and 24 h 100 mM EtOH and skeletal muscle from pair‐ and ethanol‐fed (PF and EF) mice and human patients. (A) ATAC‐seq footprinting plot showing decreased accessibility for all hypoxia‐inducible factor‐1α (aryl hydrocarbon receptor nuclear translocator) motifs at 6 h EtOH versus UnT. (B) Heatmaps of HIF1α target/signalling molecules. (C) Reporter assay for HIF1α. (D,E) Representative immunoblots and densitometry of hypoxia‐inducible factor (HIF) 1α, HIF1β, HIF2α, aryl hydrocarbon receptor (AHR). (F) Representative photomicrographs of myotubes stained with [2‐(2‐nitro‐lH‐imidazol‐l‐yl)‐N‐(2,2,3,3,3‐pentafluoropropyl) acetamide] EF5 (red) for hypoxia and 4′,6‐diamidino‐2‐phenylindole (DAPI) for nuclei in myotubes grown at 20% and 1% O_2_. Significance: ATACseq (*p* < 0.005); myotube RNAseq (*p*‐adjusted < 0.05); others (*p* < 0.05). Data mean ± SD from *n* ≥ 3 biological replicates. 2‐groups: Student's *t*‐test; > 2‐groups: One‐way ANOVA with uncorrected Fisher's LSD. *p < 0.05; **p < 0.01; ***p < 0.001. β‐actin shown for (E) HIF1β (C2C12); HIF1β (mouse). RLU: Relative luciferase units.

In silico analyses using ATACseq to evaluate chromatin accessibility and predict transcription factor binding showed reduced activity of BHL HE40 and HIF1α‐HIF1β (aryl hydrocarbon receptor nuclear translocase; ARNT), which bind to HIF1α motifs, in response to ethanol treatment (Figure 6A). These data suggest that increased HIF1α binding at these sites may reduce access for competing TFs. Targeted heatmaps of HIF1α targets and signalling molecules across multiomics studies in C2C12 myotubes showed that multiple targets were altered in response to ethanol exposure (Figures 6B and S16). RNAseq in murine myotubes and skeletal muscle from mALD and human ALD showed upregulation of a number of HIF1α targets including glucose transporters (SLC2A1, SLC2A3), glycolysis enzymes (hexokinase 1,2) and DNA damage‐inducible transcript 4 (DDIT4/REDD1). Expression of HIF1α transcriptional modulator, CREB/P300 interacting transactivator with glutamate–aspartate–rich carboxy‐terminal domain‐2 (CITED2), was also higher in ALD. Proteomics showed fewer canonical proteins than in RNAseq (Figures 6B and S16), potentially due to HIF1α‐mediated inhibition of mRNA translation or some of its target proteins (DDIT4/REDD1). Acetylomics and phosphoproteomics indicated altered post‐translational modifications in HIF1α signalling and target genes, suggesting that ethanol‐induced HIF1α targets may differ from those activated by hypoxia (Figures 6B and S16).

Consistent with our untargeted studies, a HIF1α reporter assay showed significantly increased activity (Figure 6C), and immunoblots confirmed HIF1α stabilization, reduced HIF1β expression and unchanged HIF2α expression in ethanol‐treated myotubes and gastrocnemius muscle from mALD (Figure 6D,E). Aryl hydrocarbon receptor (AHR), which competes with HIF1β for binding to HIF1α, was less in ethanol‐treated myotubes (Figure 6E). Since ethanol has been reported to cause hypoxia in hepatocytes [24], and canonical stabilization of HIF1α occurs during hypoxia, we tested whether the observed HIF1α stabilization in myotubes was due to hypoxia. However, despite HIF1α stabilization, ethanol did not induce cellular hypoxia as shown by our EF5 assay (Figure 6F).

**FIGURE 7:**
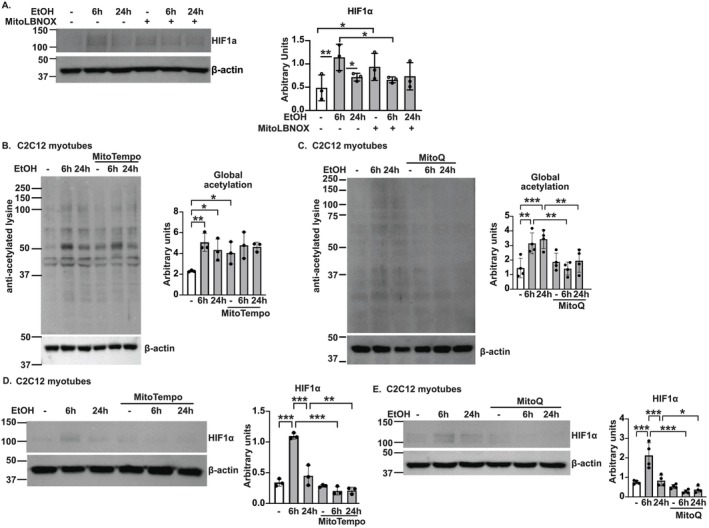
Reversal of acetylation and expression of HIF1α. Studies done in differentiated murine C2C12 myotubes treated without/with 6 or 24 h of 100 mM ethanol (EtOH) transfected with/without mitochondrial‐directed *Lactobacillus brevis* NADH oxidase (MitoLbNOX), MitoTempo or MitoQ. Representative immunoblots and densitometries of (A) hypoxia‐inducible factor‐1a (HIF1α) without/with MitoLbNOX; (B,C) global acetylation; and (D,E) HIF1α without/with MitoTempo or MitoQ. Data mean ± SD, *n ≥* 3 biological replicates. One‐way ANOVA with uncorrected Fisher's LSD. *p < 0.05; **p < 0.01; ***p < 0.001.

Ethanol‐induced HIF1α stabilization was reversed by MitoLbNOX (mitochondrial targeted *Lactobacillus brevis* NADH oxidase) (Figure 7A), indicating that ethanol‐induced stabilization of HIF1α during normoxia is redox dependent. MitoLbNOX restores redox balance by increasing NADH oxidation and decreasing free radical generation and oxidative stress [14]. Reversal of protein acetylation and HIF1α stabilization by mitochondrial‐targeted antioxidants (MitoQ) shows that both lower redox ratio and higher mitochondrial free radicals contribute to these effects in ALD (Figure 7B–E). Mitochondrial oxidative dysfunction, reduced sirtuin expression and increased protein acetylation observed in ALD are known contributors to/consequences of cellular senescence. We then experimentally assessed post‐mitotic senescence in muscle from ALD models.

### 3.6 Ethanol Causes Post‐Mitotic Senescence in Skeletal Muscle

**FIGURE 8:**
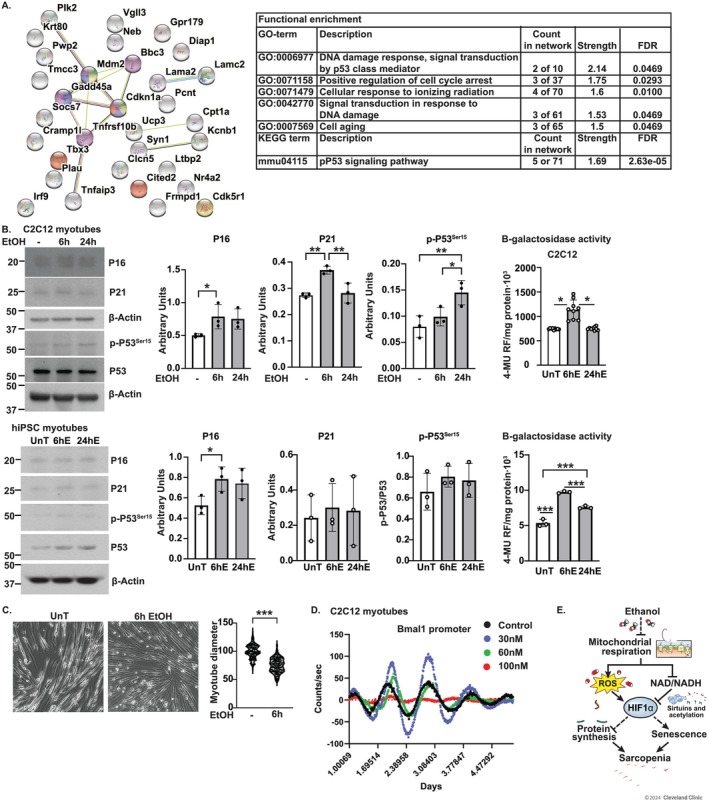
Ethanol causes senescence‐associated molecular phenotype in myotubes and skeletal muscle. Studies done in differentiated murine and human‐induced pluripotent stem cell–derived (hiPSC) myotubes treated with 100 mM ethanol (EtOH) and skeletal muscle from humans with/without alcohol‐associated cirrhosis). (A) STRING network and functional enrichment analyses from human and murine myotube RNAseq. (B) Representative immunoblots and densitometry of senescence markers, p16, p21 and phosphorylated‐p53^Ser15^ and senescence‐associated β‐galactosidase activity in murine and hiPSC myotubes. 4‐MU: 4‐methyl‐umbelliferone. RFU: Relative fluorescence unit. (C) Representative photomicrographs and diameter of hiPSC myotubes. (D) Luciferase reporter assay for Bmal1 activity. (E) Study schematic. Data mean ± SD from *n* ≥ 3. Significance cutoffs: myotube RNAseq (*p*‐adjusted < 0.05); human RNAseq (*p* < 0.05). 2‐groups: Student's *t*‐test; > 2‐groups: One‐way ANOVA with uncorrected Fisher's LSD. *p < 0.05; **p < 0.01; ***p < 0.001. β‐actin from (B) P21, p‐P53 (C2C12) and P16 (hiPSC).

Bioinformatic analyses including horizontal integration of data across models show that ethanol exposure results in functional enrichment of aging and cell cycle–related processes in shared genes with increased expression in RNAseq in murine myotubes and human skeletal muscle (Figure 8A). We have previously described a senescence‐associated molecular phenotype that includes less myotubes diameter, higher expression of p16, p21 and phosphorylated P53^Ser15^ and greater senescence‐associated β‐galactosidase activity (SABG) activity [13]. In myotubes, ethanol exposure increased the expression of p16, p21 and phosphorylated P53^Ser15^, and SABG activity was higher (Figure 8B). Expression of senescence markers was not altered in muscle from mALD (Figure S17). Similar to prior data in murine myotubes, a diameter of hiPSCm was less with ethanol treatment (Figure 8C). There is increasing recognition of the interaction between circadian rhythms and senescence. Bioinformatics analyses show enrichment of circadian rhythm signalling in ethanol‐treated myotubes and muscle from mALD (Figure S16). Experimentally, a Bmal1‐luciferase reporter showed disruption of circadian rhythm in ethanol‐treated myotubes (Figure 8D). This disruption of circadian patterns in myotubes is consistent with a prior report [25] but needs further dissection of the mechanisms and consequences, including accelerated senescence.

## 4 Discussion

Using complementary comprehensive multiomics analyses and experimental validation approach, we identified the global molecular and metabolic landscape of sarcopenia in ALD. Perturbations at multiple molecular levels in the skeletal muscle were noted with more DEMs on RNAseq than in other datasets independent of the stringency of the cutoff values. Enrichment of pathways related to mitochondrial oxidative function, HIF1a and sirtuin signalling, and senescence were noted across multiple datasets. Consistently, decreased expression of critical genes regulating mitochondrial oxidative function, antioxidants, sirtuin expression and expression of HIF1a were noted across datasets. Experimentally, consistent with our untargeted dataset analyses, we noted decreased mitochondrial oxidative function, lower redox ratio and increased protein acetylation with less sirtuin expression, with increased post‐mitotic senescence in murine myotubes, muscle from mALD and hiPSCm. Mechanistically, restoring redox ratio by increased NADH oxidation or targeting mitochondrial reactive oxygen species with mitochondrial antioxidants, HIF1α stabilization and global acetylation by ethanol were reversed. These data provide novel insights into the global molecular perturbations and help identify the multicomponent responses that contribute to sarcopenia in ALD.

Analyses of untargeted datasets across models of ethanol exposure/ALD identified distinct temporal patterns of clusters of DEMs. We noted early transient changes (an initial increase/decrease followed by a return to baseline), late changes (only delayed increase/decrease in DEM) and persistent changes (early increase/decrease that persists). Similar patterns have been reported in response to another cytotoxin, ammonia [9]. Potential reasons for this include rapid modifications, changes in translational efficiency during ethanol exposure and differences in protein stability in the molecules in different temporal clusters. Given the relatively recent identification of such global responses using large, untargeted datasets, the impact of such clustering on cellular outcomes and their potential as therapeutic targets needs to be evaluated experimentally. In our focused experimental approaches, we have identified the functional relevance of such responses on mitochondrial oxidation, HIF1a stabilization and cellular senescence.

Few studies have systematically evaluated mitochondrial structural alterations and their relation to oxidative function with conflicting data on the impact of increased fusion on oxidative phosphorylation [26, 27, 28]. Mitochondrial structural analyses on confocal microscopy in our models showed an increase in tubular and fused mitochondria with ethanol exposure suggesting more fusion and less fission events. Complementary 3D electron microscopy of muscle from mALD allowed for more detailed structural analyses compared with 2D imaging studies. In skeletal muscle, we noted increased mitochondrial length that was consistent with our confocal data. Our mitochondrial structural data are different from other reports that skeletal muscle mitochondrial fusion is decreased in an Mfn1‐dependent manner in rats fed long‐term ethanol [29]. Differences in experimental design could explain our observations of less expression of mitochondrial fission proteins FIS1 in hiPSC and MFF in gastrocnemius muscle from mALD [30]. An innovative finding of our studies was that skeletal muscle mitochondria from mALD had more cristae that were less apart. Our bioinformatics analyses of cristae regulatory molecules showed higher expression of a number of these genes. Cristae length and area have been related to the efficiency of oxidative phosphorylation and, despite more cristae, oxidative phosphorylation was less suggesting lower efficiency of mitochondrial oxidative function in response to ethanol. This can also explain the lower protein synthesis, contractile strength and human data that show limited benefit of nutrient supplementation in ALD [5, 31, 32].

In addition to the contribution of oxidative phosphorylation to cellular ATP content, maintenance of redox ratio is another critical mitochondrial function. Redox ratio also alters a number of cellular responses including protein acetylation and senescence in skeletal muscle [14] and stabilization of HIF1a in other systems [33]. HIF1a is recently recognized to be a critical transcriptional factor that regulates mitochondrial function and cellular senescence in skeletal muscle [15].

Stabilization of HIF1α in murine myotubes and muscle from mALD without hypoxia is consistent with our previous data that ethanol causes increased free radical and oxidative stress in skeletal muscle [8]. A concomitant reduction in HIF1β, a constitutively expressed protein and a required binding partner for HIF1α signalling [34, 35] were observed. In addition to binding with HIF1α, HIF1β is also a binding partner to aryl hydrocarbon receptor (AHR), and binding of HIF1α with HIF1β competitively inhibits the xenobiotic AHR pathway signalling [35, 36, 37]. Proteomics and metabolomics analyses showed enrichment of the xenobiotic metabolism and AHR signalling pathway. Similar to reports by others in hepatic stellate cells [37], our finding of a simultaneous reduction in AHR and HIF1β expression in the setting of ethanol‐induced stabilization of HIF1α lays the foundation for the evaluation of the role of AHR in mediating the consequences of ethanol.

Ethanol exposure is a known contributor to a sarcopenic phenotype and has been reported to increase the expression of skeletal muscle myostatin, a negative regulator of muscle mass [4]. Though mechanistic studies do not identify myostatin as a direct target of HIF1α, our data suggest non‐canonical regulation via acetyl‐HIF1α is a potential regulatory mechanism to explain increased myostatin during hypoxia reported by others. Interestingly and consistent with data reported by others and us, myostatin expression increased with ethanol exposure when HIF1α was stabilized and acetyl‐HIF1α was higher. Given the significant role of myostatin in sarcopenia with ethanol [4], the mechanistic link between HIF1α and myostatin needs to be evaluated.

Greater post‐mitotic senescence in cellular models including murine and hiPSCm noted in the present studies was not consistently replicated in muscle tissue from mALD that may be related to inter‐organ cross talk and contribution of hepatic metabolism of ethanol as has been shown earlier [9, 15]. Therefore, although in vitro findings can help identify the effects of alcohol and its metabolites on the muscle, it is also possible that our findings in the in vivo models (mALD, humans with ALD) may be more pronounced or altered because of additional contributions that have been identified in vivo (hyperammonaemia due to impaired ureagenesis, systemic inflammatory mediators and hepatocyte‐derived ethanol metabolites) [4]. Differences in rates of liver and muscle ethanol metabolism and metabolite concentrations (acetaldehyde) in circulation and tissue microenvironment can contribute to differences between in vitro and in vivo responses. Whether sarcopenia in ALD worsens liver injury/responses is not addressed in these studies. Of note, sarcopenia is suggested as a contributor to the progression of liver injury in non‐alcoholic fatty liver disease (NAFLD)/metabolic dysfunction associated steatotic liver disease (MASLD) and development of hepatic encephalopathy, a neurological complication of liver disease [38, 39]. Whether altered myokines/myometabolites during sarcopenia can potentially worsen liver injury in ALD with consequent perturbations in other organs needs to be explored in future studies.

In summary, we show that ethanol impairs protein synthesis and mitochondrial oxidative function with reduced expression and activity of critical components of the mitochondrial ETC and causes cellular senescence in skeletal muscle. These perturbations, including lower redox ratios, decreased sirtuin expression and increased mitochondrial protein acetylation, are linked to HIF1α stabilization and post‐mitotic senescence. Thus, complex and interrelated molecular and metabolic mechanisms drive maladaptive responses including adaptive exhaustion in ALD and may also have relevance in other organ dysfunction.

## Conflicts of Interest

The authors declare no conflicts of interest.

## Supporting information


**Data S1** Supporting information

**Data S2** Supporting information

**Table S1** Key reagents

**Table S2** Appendix of supplementary figure panels

**Table S3** Supplementary table appendix

**Table S4** Supporting information

**Table S5** Enriched pathways (by dataset, by cluster) in molecules with increased or decreased differential expression/modification

**Table S6** Shared processes (by cluster) with increased or decreased gene expression/modification

**Table S7** Enriched pathways (by cluster) in metabolites that are either increased or decreased in expression from untargeted metabolomics

**Table S8** Summary of shared pathways among metabolomics clusters

**Table S9** DAVID pathways (by cluster) related mitochondria

**Table S10** Comparative analysis of tri‐carboxylic acid (TCA) cycle processes in proteomics datasets. DEM: differentially expressed metabolites, DEP: differentially expressed proteins

**Table S11** DAVID pathways (by cluster) related to cell cycle, cell damage and senescence

**Figure S1** C2C12 assay for transposase accessible chromatin (ATACseq): QC and overall heatmap. C2C12 myotubes were differentiated and either not treated (UnT) or treated with 100 mM ethanol (EtOH) for 6 h. ATACseq was performed, and differentially accessible areas of the chromatin (DAC) were analysed. (A) Heatmap of DAC. (B) Functional enrichment study showing pathways derived from Ingenuity Pathway Analysis (IPA, QIAGEN Inc). (C) Volcano plot highlighting 25 most changed DAC (orange). (D) Functional enrichment using Gene Ontology (GO): Biological Process (BP), GO: Molecular Function (MF) and Kyoto Encyclopaedia of Genes and Genomes (KEGG). Functional enrichment analysis is used to identify biological processes, pathways or molecular functions that are overrepresented in a set of genes or proteins compared to a background set. The most enriched pathways were shown. Significance for ATACseq taken at *p* < 0.005.
**Figure S2** C2C12 bulk RNA sequencing (RNAseq) cluster heatmaps, pathways and volcano plots. C2C12 myotubes were differentiated and either not treated (UnT) or treated with 100 mM ethanol (EtOH) for 6 or 24 h. RNAseq was performed. Differentially expressed molecules (DEMs) were clustered into early transient (changed at 6 h without change at 24 h EtOH), late (unchanged at 6 h but changed at 24 h), persistent (sustained increase or decrease in expression in the same direction at both 6 and 24 h) and pseudosilent (significant change in expression between 6 and 24 h of EtOH treatment, but not significantly different expression at either treatment timepoint from untreated myotubes) and analysed. (A) Cluster heatmaps. (B) Cluster functional enrichment using Ingenuity Pathway Analysis (IPA, QIAGEN Inc). (C) Scatter plot comparing differentially accessible areas of the chromatin using assay for transposase accessible chromatin (ATACseq) and DEM on RNAseq. (D) Cluster volcano plots highlighting the 25 most changed DEM (orange). (E–G) Cluster functional enrichment using g:Profiler using Gene Ontology (GO): Biological Process (BP), GO: Molecular Function (MF), Kyoto Encyclopaedia of Genes and Genomes (KEGG), REACTOME (REAC) and WikiPathways (WP) for (E) early transient (F) late, (G) persistent and (H) pseudosilent clusters. Functional enrichment analysis is used to identify biological processes, pathways or molecular functions that are overrepresented in a set of genes or proteins compared with a background set. The most enriched pathways were shown for each clusterSignificance for DEM in RNAseq was taken at *p*‐adjusted < 0.05 and DAC for ATACseq at *p* < 0.005. Pathway significance −log(*p*‐value) ≥ 1.3.
**Figure S3** C2C12 untargeted proteomics cluster heatmaps, pathways and volcano plots. C2C12 myotubes were differentiated and either not treated (UnT) or treated with 100 mM ethanol (EtOH) for 6 or 24 h. Untargeted proteomics using gas‐chromatography mass spectrometry was performed. Differentially expressed molecules (DEMs) were clustered into early transient (changed at 6 h without change at 24 h EtOH), late (unchanged at 6 h but changed at 24 h) and persistent (sustained increase or decrease in expression in the same direction at both 6 and 24 h). (A) Cluster heatmaps. (B) Cluster functional enrichment using Ingenuity Pathway Analysis (IPA, QIAGEN Inc). (C) Cluster volcano plots highlighting the 25 most changed DEM (orange). (D–F) Cluster functional enrichment using g:Profiler using Gene Ontology (GO): Biological Process (BP), GO: Molecular Function (MF), Comprehensive Resource of Mammalian Protein (CORUM), Kyoto Encyclopaedia of Genes and Genomes (KEGG), REACTOME (REAC) and WikiPathways (WP) for (D) early transient (E) late and (F) persistent clusters. Functional enrichment analysis is used to identify biological processes, pathways or molecular functions that are overrepresented in a set of genes or proteins compared to a background set. The most enriched pathways were shown for each cluster. Significance for DEM in proteomics was taken at *p* < 0.05. Pathway significance −log(*p*‐value) ≥ 1.3.
**Figure S4** Human‐induced pluripotent stem cell–derived (hiPSC) myotube RNA‐sequencing (RNAseq) cluster heatmaps, pathways and volcano plots. hiPSC‐derived myotubes were differentiated and either not treated (UnT) or treated with 100 mM ethanol (EtOH) for 6 or 24 h. RNAseq was performed. Differentially expressed molecules (DEMs) were clustered into early transient (changed at 6 h without change at 24 h EtOH), late (unchanged at 6 h but changed at 24 h), persistent (sustained increase or decrease in expression in the same direction at both 6 and 24 h) and pseudosilent (significant change in expression between 6 and 24 h of EtOH treatment, but not significantly different expression at either treatment timepoint from untreated myotubes) and analysed. (A) Cluster heatmaps. (B) Cluster functional enrichment using Ingenuity Pathway Analysis (IPA, QIAGEN Inc). (C) Cluster volcano plots highlighting the 25 most changed DEM (orange). (D–G) Cluster functional enrichment using g:Profiler using Gene Ontology (GO): Biological Process (BP), GO: Molecular Function (MF), Kyoto Encyclopaedia of Genes and Genomes (KEGG), REACTOME (REAC) and WikiPathways (WP) for (D) early transient (E) late, (F) persistent and (G) pseudosilent cluster. Functional enrichment analysis is used to identify biological processes, pathways or molecular functions that are overrepresented in a set of genes or proteins compared with a background set. The most enriched pathways were shown for each cluster. Significance for DEM in RNAseq was taken at adjusted *p*‐value < 0.05. Pathway significance −log(*p*‐value) ≥ 1.3.
**Figure S5** Human‐induced pluripotent stem cell–derived (hiPSC) myotube untargeted proteomics cluster heatmaps, pathways and volcano plots. Human‐induced pluripotent stem cell–derived myotubes were differentiated and either not treated (UnT) or treated with 100 mM ethanol (EtOH) for 6 or 24 h. Untargeted proteomics using gas‐chromatography mass spectrometry was performed. Differentially expressed molecules (DEMs) were clustered into early transient (changed at 6 h without change at 24 h EtOH), late (unchanged at 6 h but changed at 24 h), persistent (sustained increase or decrease in expression in the same direction at both 6 and 24 h) and pseudosilent (significant change in expression between 6 and 24 h of EtOH treatment, but not significantly different expression at either treatment timepoint from untreated myotubes) and analysed. (A) Cluster heatmaps. (B) Cluster functional enrichment using Ingenuity Pathway Analysis (IPA, QIAGEN Inc). (C) Cluster volcano plots highlighting the 25 most changed DEM (orange). (D–G) Cluster functional enrichment using g:Profiler using Gene Ontology (GO): Biological Process (BP), GO: Molecular Function (MF), Comprehensive Resource of Mammalian Protein (CORUM), Kyoto Encyclopaedia of Genes and Genomes (KEGG), REACTOME (REAC) and WikiPathways (WP) for (D) early transient (E) late, (F) persistent and (G) pseudosilent cluster. Functional enrichment analysis is used to identify biological processes, pathways or molecular functions that are overrepresented in a set of genes or proteins compared with a background set. The most enriched pathways were shown for each cluster. Significance for DEM in hiPSC proteomics was taken at *p* < 0.05. Pathway significance −log(*p*‐value) ≥ 1.3.
**Figure S6** C2C12 untargeted acetylomics cluster heatmaps, pathways and volcano plots. C2C12 myotubes were differentiated and either not treated (UnT) or treated with 100 mM ethanol (EtOH) for 6 or 24 h. Untargeted acetylomics using gas‐chromatography mass spectrometry was performed. Differentially expressed molecules (DEMs) were clustered into early transient (changed at 6 h without change at 24 h EtOH), late (unchanged at 6 h but changed at 24 h), persistent (sustained increase or decrease in expression in the same direction at both 6 and 24 h) and pseudosilent (significant change in expression between 6 and 24 h of EtOH treatment, but not significantly different expression at either treatment timepoint from untreated myotubes) and analysed. (A) Cluster heatmaps. (B) Cluster functional enrichment using Ingenuity Pathway Analysis (IPA, QIAGEN Inc). (C) Cluster volcano plots highlighting the 25 most changed DEM (orange). (D–G) Cluster functional enrichment using g:Profiler using Gene Ontology (GO): Biological Process (BP), GO: Molecular Function (MF), Kyoto Encyclopaedia of Genes and Genomes (KEGG), REACTOME (REAC) and WikiPathways (WP) for (D) early transient (E) late, (F) persistent and (G) pseudosilent clusters. Functional enrichment analysis is used to identify biological processes, pathways or molecular functions that are overrepresented in a set of genes or proteins compared with a background set. The most enriched pathways were shown for each cluster. Significance for DEM in acetylomics was taken at *p*‐value < 0.05. Pathway significance −log(*p*‐value) ≥ 1.3.
**Figure S7** C2C12 untargeted phosphoproteomics cluster heatmaps, pathways and volcano plots. C2C12 myotubes were differentiated and either not treated (UnT) or treated with 100 mM ethanol (EtOH) for 6 or 24 h. Untargeted acetylomics using gas‐chromatography mass spectrometry was performed. Differentially expressed molecules (DEMs) were clustered into early transient (changed at 6 h without change at 24 h EtOH), late (unchanged at 6 h but changed at 24 h), persistent (sustained increase or decrease in expression in the same direction at both 6 and 24 h) and pseudosilent (significant change in expression between 6 and 24 h of EtOH treatment, but not significantly different expression at either treatment timepoint from untreated myotubes) and analysed. (A) Cluster heatmaps. (B) Cluster functional enrichment using Ingenuity Pathway Analysis (IPA, QIAGEN Inc). (C) Cluster volcano plots highlighting the 25 most changed DEM (orange). (D–G) Cluster functional enrichment using g:Profiler using Gene Ontology (GO): Biological Process (BP), GO: Molecular Function (MF), Kyoto Encyclopaedia of Genes and Genomes (KEGG), REACTOME (REAC) and WikiPathways (WP) for (D) early transient (E) late, (F) persistent and (G) pseudosilent clusters. Functional enrichment analysis is used to identify biological processes, pathways or molecular functions that are overrepresented in a set of genes or proteins compared with a background set. The most enriched pathways were shown for each cluster. Significance for DEM in acetylomics was taken at *p*‐value < 0.05. Pathway significance −log(*p*‐value) ≥ 1.3.
**Figure S8** C2C12 metabolomics and integration with proteome. C2C12 myotubes were differentiated and either not treated (UnT) or treated with 100 mM ethanol (EtOH) for 6 or 24 h. Untargeted metabolomics using high‐performance liquid chromatography (HPLC) using an Agilent C18 column and hydrophilic interaction liquid chromatography (HILIC) were performed using gas‐chromatography/liquid‐chromatography mass spectrometry. (A) Heatmaps and principal component analysis (PCA) plots shown for negative (Neg) and positive (Pos) Ion modes using C18 and HILIC columns. (B) Pathway enrichment analysis performed using MetaboAnalyst is shown as pathway impact plots. Differentially expressed molecules (DEMs) were clustered into early transient (changed at 6 h without change at 24 h EtOH), late (unchanged at 6 h but changed at 24 h), persistent (sustained increase or decrease in expression in the same direction at both 6 and 24 h) and pseudosilent (significant change in expression between 6 and 24 h of EtOH treatment, but not significantly different expression at either treatment timepoint from untreated myotubes) and analysed. Blue solid line indicates significance threshold at −log_10_(*p*‐value) ≥ 1.3. (C) Pathway impact plot and STRING network analyses showing pathway enrichment and molecular connectedness of horizontally integrated proteomic and metabolomic data C2C12 myotube proteomics and metabolomics. Pathway impact plots: Pathways with greater significance are represented by ‘warmer’ or more red, colour of dot. Larger dot size indicates greater pathway connectivity to metabolic systems. STRING network: green = decreased expression, red = increased expression, grey = no differential/unchanged expression. Significance for differentially expressed molecules on metabolomics was set using Welch’s *t*‐test *p* < 0.05 and for proteomics Student’s *t*‐test *p* < 0.05.
**Figure S9** Mouse RNAseq and proteomics. Wild‐type C57BL/6J mice were or pair‐fed (PF) or fed ethanol (EF) to generate a mouse model of alcohol associated liver disease (mALD). Gastrocnemius muscle was obtained and bulk RNA sequencing (RNAseq) and proteomics using gas‐chromatography mass spectrometry were performed. Functional enrichment using g:Profiler using Gene Ontology (GO): Biological Process (BP), GO: Molecular Function (MF), Comprehensive Resource of Mammalian Protein (CORUM), Kyoto Encyclopaedia of Genes and Genomes (KEGG), REACTOME (REAC) and WikiPathways (WP) using differentially expressed molecules (DEMs) from (A) RNAseq and (B) untargeted proteomics. Significance for DEM was set at *p* < 0.05.
**Figure S10** Human RNAseq and proteomics. Vastus lateralis muscle from humans without (CTL) or with alcohol associated cirrhosis (CIR) were obtained and bulk RNA sequencing (RNAseq) and proteomics using gas‐chromatography mass spectrometry were performed. Functional enrichment using g:Profiler using Gene Ontology (GO): Biological Process (BP), GO: Molecular Function (MF), Comprehensive Resource of Mammalian Protein (CORUM), Kyoto Encyclopaedia of Genes and Genomes (KEGG), REACTOME (REAC) and WikiPathways (WP) using differentially expressed molecules (DEMs) from (A) RNAseq and (B) proteomics. Significance for differentially expressed genes and proteins set at *p* < 0.05.
**Figure S11** Cell cluster IPA comparison analysis. Ingenuity Pathway Analysis (QIAGEN Inc.) was performed for the overall cell clusters for (A) persistent and (B) pseudosilent clusters. Significance threshold set at −log_10_(*p*‐value) ≥ 1.3.
**Figure S12** Targeted hierarchical scatter plots. Scatterplots are shown for differentially expressed moledugenes that are significant within (A) senescence‐related molecules (using CellAge and CSgene), (B) TCA cycle, (C) MitoCarta3.0 and (D) HIF1α signalling genelists.
**Figure S13** Mitochondrial markers of fusion are not different with ethanol treatment in myotubes and mouse skeletal muscle. C2C12 and human‐induced pluripotent stem cell–derived (hiPSC) myotubes were differentiated and either not treated (UnT) or treated with 100 mM ethanol (EtOH) for 6 or 24 h. Skeletal muscle was taken from a mouse model of alcohol associated liver disease (mALD), where mice were either ethanol‐fed (EF) or pair‐fed (PF). Representative densitometry and immunoblots of mitochondrial structural proteins Mitofusin 1 (MFN1), Mitofusin 2 (MFN2) and Optic Atrophy 1 (OPA1) in (A) C2C12 and (B) hiPSC myotubes. (C) Mitochondrial respiration measured by high‐resolution respirofluorometry with substrate–uncoupler–inhibitor titration protocols. Oxidative phosphorylation + ADP(D), +Succinate(S), Complex(C) II and CIV respiration measured. (D) Representative densitometry and immunoblots of MFN1, MFN2 and OPA1 in mouse skeletal muscle. Representative loading controls (B‐actin) shown are from the following immunoblots: A. MFN2, B. DRP1, D. MFN2. Data as mean ± SD, *n* ≥ 3 biological replicates. 2‐group: Student’s *t*‐test. > 2‐group: One‐way ANOVA with uncorrected Fisher’s LSD.
**Figure S14** Sirtuin expression is unchanged in human‐induced pluripotent stem cell–derived (hiPSC) myotubes. hiPSC myotubes were differentiated and either not treated (UnT) or treated with 100 mM ethanol (EtOH) for 6 or 24 h. Representative densitometry and immunoblots for Sirtuin 1–7 in hiPSC are shown for *n* = 3 biological replicates. Data shown as mean ± SD. > 2‐group: One‐way ANOVA with uncorrected Fisher’s LSD.
**Figure S15** Acetylated (Ac)‐p65NFkB expression is higher with ethanol in myotubes. C2C12 myotubes were differentiated and either not treated (−) or treated with 100 mM ethanol (EtOH) for 6 or 24 h. Representative immunoblots and densitometry for Ac‐p65NFkB. Data shown as mean ± SD. > 2‐group: One‐way ANOVA with uncorrected Fisher’s LSD. *p < 0.05. *n* = 3 biological replicates
**Figure S16** Genelist heatmaps. C2C12 myotubes were differentiated and either not treated (UnT) or treated with 100 mM ethanol (EtOH) for 6 or 24 h. Skeletal muscle was taken from a mouse model of alcohol‐associated liver disease (mALD) and from human subjects with alcohol‐associated cirrhosis and compared to their respective controls. Heatmaps are shown for differentially expressed molecules identified in (A) HIF1α targets on phosphoproteomics, acetylomics and proteomics. (B) Senescence‐related molecules (using CellAge and CSgene), MitoCarta3.0 and HIF1α signalling in C2C12 myotube proteomics. (C) Circadian rhythm–related molecules in C2C12 myotube ATACseq, proteomics, acetylomics and phosphoproteomics. Significance: ATACseq (*p* < 0.005); myotube RNAseq (*p*‐adjusted < 0.05); others (*p* < 0.05).
**Figure S17** Senescence markers are unchanged with acute‐on‐chronic ethanol feeding in mouse skeletal muscle. Wild‐type C57BL/6J mice were or pair‐fed (PF) or fed ethanol (EF) to generate a mouse model of alcohol associated liver disease (mALD). Representative immunoblots and densitometry of senescence markers, p16, p21 and phosphorylated‐p53^Ser15^ mouse skeletal muscle. Representative loading control (β‐actin) shown was taken from the P53 membrane. All experimental data mean ± SD from *n* ≥ 3. Data shown as mean ± SD.
