# Supplementary material for: Integrated Multiomics Analyses of the Molecular Landscape of Sarcopenia in Alcohol‐Related Liver Disease
Source: J Cachexia Sarcopenia Muscle. 2025 Apr 30;16(3):e13818. doi: 10.1002/jcsm.13818 (PMC12044136; doi:10.1002/jcsm.13818)
Supplement: Supplementary file 1 — Data S1 Supporting information [file JCSM-16-e13818-s002.docx]

**Supplementary Figure Legends**

**S.Fig 1. C2C12 Assay for transposase accessible chromatin (ATACseq): QC and overall heatmap.**

C2C12 myotubes were differentiated and either not treated (UnT) or treated with 100mM ethanol (EtOH) for 6h. ATACseq was performed, and differentially accessible areas of the chromatin (DAC) were analyzed. **A.** Heatmap of DAC. **B.** Functional enrichment study showing pathways derived from Ingenuity Pathway Analysis (IPA, QIAGEN, Inc). **C.** Volcano plot highlighting 25 most changed DAC (orange). **D**. Functional enrichment using Gene Ontology (GO): Biological Process (BP), GO: Molecular Function (MF), and Kyoto Encyclopedia of Genes and Genomes (KEGG). Functional enrichment analysis is used to identify biological processes, pathways, or molecular functions that are overrepresented in a set of genes or proteins compared to a background set. The most enriched pathways were shown. Significance for ATACseq taken at p<0.005.

**S.Fig 2. C2C12 bulk RNA sequencing (RNAseq) cluster heatmaps, pathways, and volcano plots.** C2C12 myotubes were differentiated and either not treated (UnT) or treated with 100mM ethanol (EtOH) for 6 or 24h. RNAseq was performed. Differentially expressed molecules (DEM) were clustered into Early Transient (changed at 6h without change at 24h EtOH), Late (unchanged at 6h but changed at 24h), Persistent (sustained increase or decrease in expression in the same direction at both 6h and 24h), and Pseudosilent (significant change in expression between 6h and 24h of EtOH treatment, but not significantly different expression at either treatment timepoint from untreated myotubes) and analyzed. **A.** Cluster heatmaps. **B**. Cluster functional enrichment using Ingenuity Pathway Analysis (IPA, QIAGEN, Inc). **C.** Scatter plot comparing differentially accessible areas of the chromatin using assay for transposase accessible chromatin (ATACseq) and DEM on RNAseq**. D.** Cluster volcano plots highlighting the 25 most changed DEM (orange). **E-G**. Cluster functional enrichment using g:Profiler using Gene Ontology (GO): Biological Process (BP), GO: Molecular Function (MF), Kyoto Encyclopedia of Genes and Genomes (KEGG), REACTOME (REAC), and WikiPathways (WP) for **E.** Early transient **F.** Late, **G.** Persistent, **H.** Pseudosilent cluster. Functional enrichment analysis is used to identify biological processes, pathways, or molecular functions that are overrepresented in a set of genes or proteins compared to a background set. The most enriched pathways were shown for each clusterSignificance for DEM in RNAseq was taken at padj<0.05 and DAC for ATACseq at p<0.005. Pathway significance -log(p-value)>1.3.

**S.Fig 3. C2C12 untargeted proteomics cluster heatmaps, pathways, and volcano plots.** C2C12 myotubes were differentiated and either not treated (UnT) or treated with 100mM ethanol (EtOH) for 6 or 24h. Untargeted proteomics using gas-chromatography mass spectrometry was performed. Differentially expressed molecules (DEM) were clustered into Early Transient (changed at 6h without change at 24h EtOH), Late (unchanged at 6h but changed at 24h), Persistent (sustained increase or decrease in expression in the same direction at both 6h and 24h). **A.** Cluster heatmaps. **B**. Cluster functional enrichment using Ingenuity Pathway Analysis (IPA, QIAGEN, Inc). **C**. Cluster volcano plots highlighting the 25 most changed DEM (orange). **D-F**. Cluster functional enrichment using g:Profiler using Gene Ontology (GO): Biological Process (BP), GO: Molecular Function (MF), Comprehensive Resource of Mammalian Protein (CORUM), Kyoto Encyclopedia of Genes and Genomes (KEGG), REACTOME (REAC), and WikiPathways (WP) for **D.** Early transient **E.** Late, and **F.** Persistent clusters. Functional enrichment analysis is used to identify biological processes, pathways, or molecular functions that are overrepresented in a set of genes or proteins compared to a background set. The most enriched pathways were shown for each cluster. Significance for DEM in proteomics was taken at p<0.05. Pathway significance -log(p-value)>1.3.

**S.Fig 4. Human induced pluripotent stem cell-derived (hiPSC) myotube RNA-sequencing (RNAseq) cluster heatmaps, pathways, and volcano plots.** hiPSC-derived myotubes were differentiated and either not treated (UnT) or treated with 100mM ethanol (EtOH) for 6 or 24h. RNAseq was performed. Differentially expressed molecules (DEM) were clustered into Early Transient (changed at 6h without change at 24h EtOH), Late (unchanged at 6h but changed at 24h), Persistent (sustained increase or decrease in expression in the same direction at both 6h and 24h), and Pseudosilent (significant change in expression between 6h and 24h of EtOH treatment, but not significantly different expression at either treatment timepoint from untreated myotubes) and analyzed. **A.** Cluster heatmaps. **B**. Cluster functional enrichment using Ingenuity Pathway Analysis (IPA, QIAGEN, Inc). **C**. Cluster volcano plots highlighting the 25 most changed DEM (orange). **D-G**. Cluster functional enrichment using g:Profiler using Gene Ontology (GO): Biological Process (BP), GO: Molecular Function (MF), Kyoto Encyclopedia of Genes and Genomes (KEGG), REACTOME (REAC), and WikiPathways (WP) for **D.** Early transient **E.** Late, **F.** Persistent, **G.** Pseudosilent cluster. Functional enrichment analysis is used to identify biological processes, pathways, or molecular functions that are overrepresented in a set of genes or proteins compared to a background set. The most enriched pathways were shown for each cluster. Significance for DEM in RNAseq was taken at adjusted p-value<0.05. Pathway significance -log(p-value)>1.3.

**S.Fig 5. Human induced pluripotent stem cell derived (hiPSC) myotube untargeted proteomics cluster heatmaps, pathways, and volcano plots.** Human induced pluripotent stem cell-derived myotubes were differentiated and either not treated (UnT) or treated with 100mM ethanol (EtOH) for 6 or 24h. Untargeted proteomics using gas-chromatography mass spectrometry was performed. Differentially expressed molecules (DEM) were clustered into Early Transient (changed at 6h without change at 24h EtOH), Late (unchanged at 6h but changed at 24h), Persistent (sustained increase or decrease in expression in the same direction at both 6h and 24h), and Pseudosilent (significant change in expression between 6h and 24h of EtOH treatment, but not significantly different expression at either treatment timepoint from untreated myotubes) and analyzed. **A.** Cluster heatmaps. **B**. Cluster functional enrichment using Ingenuity Pathway Analysis (IPA, QIAGEN, Inc). **C**. Cluster volcano plots highlighting the 25 most changed DEM (orange). **D-G**. Cluster functional enrichment using g:Profiler using Gene Ontology (GO): Biological Process (BP), GO: Molecular Function (MF), Comprehensive Resource of Mammalian Protein (CORUM), Kyoto Encyclopedia of Genes and Genomes (KEGG), REACTOME (REAC), and WikiPathways (WP) for **D.** Early transient **E.** Late, **F.** Persistent, **G.** Pseudosilent cluster. Functional enrichment analysis is used to identify biological processes, pathways, or molecular functions that are overrepresented in a set of genes or proteins compared to a background set. The most enriched pathways were shown for each cluster. Significance for DEM in hiPSC proteomics was taken at p<0.05. Pathway significance -log(p-value)>1.3.

**S.Fig 6. C2C12 untargeted acetylomics cluster heatmaps, pathways, and volcano plots.** C2C12 myotubes were differentiated and either not treated (UnT) or treated with 100mM ethanol (EtOH) for 6 or 24h. Untargeted acetylomics using gas-chromatography mass spectrometry was performed. Differentially expressed molecules (DEM) were clustered into Early Transient (changed at 6h without change at 24h EtOH), Late (unchanged at 6h but changed at 24h), Persistent (sustained increase or decrease in expression in the same direction at both 6h and 24h), and Pseudosilent (significant change in expression between 6h and 24h of EtOH treatment, but not significantly different expression at either treatment timepoint from untreated myotubes) and analyzed. **A.** Cluster heatmaps. **B**. Cluster functional enrichment using Ingenuity Pathway Analysis (IPA, QIAGEN, Inc). **C**. Cluster volcano plots highlighting the 25 most changed DEM (orange). **D-G**. Cluster functional enrichment using g:Profiler using Gene Ontology (GO): Biological Process (BP), GO: Molecular Function (MF), Kyoto Encyclopedia of Genes and Genomes (KEGG), REACTOME (REAC), and WikiPathways (WP) for **D.** Early transient **E.** Late, **F.** Persistent, **G.** Pseudosilent cluster. Functional enrichment analysis is used to identify biological processes, pathways, or molecular functions that are overrepresented in a set of genes or proteins compared to a background set. The most enriched pathways were shown for each cluster. Significance for DEM in acetylomics was taken at pvalue<0.05. Pathway significance -log(p-value)>1.3.

**S.Fig 7. C2C12 untargeted phosphoproteomics cluster heatmaps, pathways, and volcano plots.** C2C12 myotubes were differentiated and either not treated (UnT) or treated with 100mM ethanol (EtOH) for 6 or 24h. Untargeted acetylomics using gas-chromatography mass spectrometry was performed. Differentially expressed molecules (DEM) were clustered into Early Transient (changed at 6h without change at 24h EtOH), Late (unchanged at 6h but changed at 24h), Persistent (sustained increase or decrease in expression in the same direction at both 6h and 24h), and Pseudosilent (significant change in expression between 6h and 24h of EtOH treatment, but not significantly different expression at either treatment timepoint from untreated myotubes) and analyzed. **A.** Cluster heatmaps. **B**. Cluster functional enrichment using Ingenuity Pathway Analysis (IPA, QIAGEN, Inc). **C**. Cluster volcano plots highlighting the 25 most changed DEM (orange). **D-G**. Cluster functional enrichment using g:Profiler using Gene Ontology (GO): Biological Process (BP), GO: Molecular Function (MF), Kyoto Encyclopedia of Genes and Genomes (KEGG), REACTOME (REAC), and WikiPathways (WP) for **D.** Early transient **E.** Late, **F.** Persistent, **G.** Pseudosilent cluster. Functional enrichment analysis is used to identify biological processes, pathways, or molecular functions that are overrepresented in a set of genes or proteins compared to a background set. The most enriched pathways were shown for each cluster. Significance for DEM in acetylomics was taken at pvalue<0.05. Pathway significance -log(p-value)>1.3.

**S.Fig 8. C2C12 metabolomics and integration with proteome.** C2C12 myotubes were differentiated and either not treated (UnT) or treated with 100mM ethanol (EtOH) for 6 or 24h. Untargeted metabolomics using high-performance liquid chromatography (HPLC) using an Agilent C18 column and hydrophilic interaction liquid chromatography (HILIC) were performed using gas-chromatography/liquid-chromatography mass spectrometry. **A**. Heatmaps and principal component analysis (PCA) plots shown for negative (Neg) and positive (Pos) Ion modes using C18 and HILIC columns. **B**. Pathway enrichment analysis performed using MetaboAnalyst are shown as pathway impact plots. Differentially expressed molecules (DEM) were clustered into Early Transient (changed at 6h without change at 24h EtOH), Late (unchanged at 6h but changed at 24h), Persistent (sustained increase or decrease in expression in the same direction at both 6h and 24h), and Pseudosilent (significant change in expression between 6h and 24h of EtOH treatment, but not significantly different expression at either treatment timepoint from untreated myotubes) and analyzed. Blue solid line indicates significance threshold at -log_10_(p-value)>1.3. **C**. Pathway impact plot and STRING network analyses showing pathway enrichment and molecular connectedness of horizontally integrated proteomic and metabolomic data C2C12 myotube proteomics and metabolomics. Pathway impact plots: Pathways with greater significance are represented by “warmer”, or more red, color of dot. Larger dot size indicates greater pathway connectivity to metabolic systems. STRING network: Green = decreased expression, Red = increased expression, gray= no differential/unchanged expression. Significance for differentially expressed molecules on metabolomics was set using Welch’s t-test p<0.05 and for proteomics Student’s t-test p<0.05.

**S.Fig 9. Mouse RNAseq and proteomics**. Wild-type C57BL/6J mice were or pair-fed (PF) or fed ethanol (EF) to generate a mouse model of alcohol associated liver disease (mALD). Gastrocnemius muscle was obtained and bulk RNA sequencing (RNAseq) and proteomics using gas chromatography mass spectrometry were performed. Functional enrichment using g:Profiler using Gene Ontology (GO): Biological Process (BP), GO: Molecular Function (MF), Comprehensive Resource of Mammalian Protein (CORUM), Kyoto Encyclopedia of Genes and Genomes (KEGG), REACTOME (REAC), and WikiPathways (WP) using differentially expressed molecules (DEM) from **A**. RNAseq **B**. Untargeted proteomics. Significance for DEM was set at p<0.05.

**S.Fig 10. Human RNAseq and proteomics.** Vastus lateralis muscle from humans without (CTL) or with alcohol associated cirrhosis (CIR) were obtained and bulk RNA sequencing (RNAseq) and proteomics using gas chromatography mass spectrometry were performed. Functional enrichment using g:Profiler using Gene Ontology (GO): Biological Process (BP), GO: Molecular Function (MF), Comprehensive Resource of Mammalian Protein (CORUM), Kyoto Encyclopedia of Genes and Genomes (KEGG), REACTOME (REAC), and WikiPathways (WP) using differentially expressed molecules (DEM) from **A**. RNAseq **B**. Proteomics. Significance for differentially expressed genes and proteins set at p<0.05.

**S.Fig 11. Cell cluster IPA comparison analysis.** Ingenuity Pathway Analysis (QIAGEN, Inc.) was performed for the overall cell clusters for **A**. Persistent and **B**. Pseudosilent clusters. Significance threshold set at -log_10_(p-value)>1.3.

**S.Fig 12. Targeted hierarchical scatter plots.** Scatterplots are shown for differentially expressed moledugenes that are significant within **A**. Senescence related-molecules (using CellAge and CSgene). **B**. TCA cycle, **C**. MitoCarta3.0, and **D**. HIF1α signaling genelists.

**S.Fig 13. Mitochondrial markers of fusion are not different with ethanol treatment in myotubes and mouse skeletal muscle**. C2C12 and human induced pluripotent stem cell-derived (hiPSC) myotubes were differentiated and either not treated (UnT) or treated with 100mM ethanol (EtOH) for 6 or 24h. Skeletal muscle was taken from a mouse model of alcohol associated liver disease (mALD), where mice were either ethanol-fed (EF) or pair-fed (PF). Representative densitometry and immunoblots of mitochondrial structural proteins Mitofusin 1 (MFN1), Mitofusin 2 (MFN2), Optic Atrophy 1 (OPA1) in (**A**)C2C12 and (**B**) hiPSC myotubes. **C**. Mitochondrial respiration measured by high resolution respirofluorometry with substrate-uncoupler-inhibitor titration protocols. Oxidative phosphorylation + ADP(D), +Succinate(S), Complex(C) II, and CIV respiration measured. **D**. Representative densitometry and immunoblots of MFN1, MFN2, and OPA1 in mouse skeletal muscle. Representative loading controls (B-actin) shown are from the following immunoblots: A.MFN2, B.DRP1, D. MFN2. Data as mean+SD, n>3 biological replicates. 2-group: Student’s t-test. >2-group: One-way ANOVA with uncorrected Fisher’s LSD.

**S.Fig 14. Sirtuin expression is unchanged in human induced pluripotent stem cell derived (hiPSC) myotubes.** hiPSC myotubes were differentiated and either not treated (UnT) or treated with 100mM ethanol (EtOH) for 6 or 24h. Representative densitometry and immunoblots for Sirtuin 1-7 in hiPSC are shown for n=3 biological replicates. Data shown as mean+SD. >2-group: One-way ANOVA with uncorrected Fisher’s LSD.

**S.Fig 15**. **Acetylated (Ac)-p65NFkB expression is higher with ethanol in myotubes**. C2C12 myotubes were differentiated and either not treated (-) or treated with 100mM ethanol (EtOH) for 6 or 24h. Representative immunoblots and densitometry for Ac-p65NFkB. Data shown as mean+SD. >2-group: One-way ANOVA with uncorrected Fisher’s LSD. *p<0.05. n=3 biological replicates

**S.Fig 16. Genelist heatmaps.** C2C12 myotubes were differentiated and either not treated (UnT) or treated with 100mM ethanol (EtOH) for 6 or 24h. Skeletal muscle was taken from a mouse model of alcohol associated liver disease (mALD) and from human subjects with alcohol-associated cirrhosis and compared to their respective controls. Heatmaps are shown for differentially expressed molecules identified in **A**. HIF1α targets on phosphoproteomics, acetylomics, proteomics. **B**. Senescence related-molecules (using CellAge and CSgene), MitoCarta3.0, and HIF1α signaling in C2C12 myotube proteomics. **C**. Circadian rhythm-related molecules in C2C12 myotube ATACseq, proteomics, acetylomics, and phosphoproteomics. Significance: ATACseq (p<0.005); myotube RNAseq (p-adjusted<0.05); others (p<0.05).

**S.Fig 17.** **Senescence markers are unchanged with acute-on-chronic ethanol feeding in mouse skeletal muscle**. Wild-type C57BL/6J mice were or pair-fed (PF) or fed ethanol (EF) to generate a mouse model of alcohol associated liver disease (mALD). Representative immunoblots and densitometry of senescence markers, p16, p21 and phosphorylated-p53^Ser15^ mouse skeletal muscle. Representative loading control (β-actin) shown was taken from the P53 membrane. All experimental data mean±SD from n≥3. Data shown as mean+SD.
