# Supplementary material for: Integrated Multiomics Analyses of the Molecular Landscape of Sarcopenia in Alcohol‐Related Liver Disease
Source: J Cachexia Sarcopenia Muscle. 2025 Apr 30;16(3):e13818. doi: 10.1002/jcsm.13818 (PMC12044136; doi:10.1002/jcsm.13818)
Supplement: Supplementary file 2 — Data S2 Supporting information [file JCSM-16-e13818-s007.docx]

**Model and Subject Details**

***In vitro* studies**

**Murine myotubes.** Murine myotubes were generated from C2C12 myoblasts (ATCC) grown to confluence in Dulbecco’s modified Eagle’s medium (DMEM) with 10% fetal bovine serum. Myoblasts were differentiated to myotubes in DMEM with 2% horse serum for 48 hours, and studies were done in untreated or treated with 100mM ethanol for 3, 6 or 24 hours. Standard protocols in our laboratory were used for cell lysates and protein extraction[1]. We have previously reported that our in vitro model used in these studies replicates the sarcopenic phenotype and molecular and functional responses of skeletal muscle in vivo in ALD (mALD, humans with ALD) and have reported over 90% viability in myotubes treated with and without 6h ethanol[1].

**Human induced pluripotent stem cell derived myotubes**. Human induced pluripotent stem cells (hiPSC) were cultured as previously described[2]. In brief, hiPSC were cultured in mTeSR medium (Stem Cell Technologies, Cambridge, MA) with myogenic induction initiated by sequential culture in skeletal muscle induction medium (AMS Biotechnology, Abingdon, UK), myoblast medium (Amsbio-SKM02) and myotube medium (AMS Biotechnology, Abingdon, UK).

***In vivo* studies**

**Mouse model.** 8-10 week-old female C57BL/6 mice (Jackson laboratory) were either pair-fed (PF) or ethanol-fed (EF, mALD). Details of these resources have been published earlier[1]. In brief, mice were allowed unrestricted access to a Lieber-DeCarli liquid diet (Dyets Inc, 710260) containing ethanol (n=4 in each EF group) or maltodextrins isocalorically substituted (n=4 in each PF group). The animals were housed in the biological resource unit under a 12-hour light/day cycle. After acclimating to the control liquid diet for 2 days, the EF group received a liquid diet with 1% ethanol (5.5% total calories) for 2 days, followed by 6% ethanol (32% total calories) for 2 days, a model of ethanol-related liver injury that increases hepatic alanine aminotransferase and triglycerides by us and others[3-5].

Mice were euthanized between 9-11 AM to minimize the effects of circadian changes. The gastrocnemius muscle was harvested, weighed, and a portion was used for cryosections and mitochondrial studies as previously described[1]. The remaining tissue was flash-frozen in liquid nitrogen and stored at −80°C for subsequent assays. *Ethical approval*. All animal protocols and procedures were conducted in accordance with the NIH Guidelines for the Care and Use of Laboratory Animals and the Cleveland Clinic Institutional Animal Care and Use Committee (IACUC, protocol 0000-2610).

We did not measure the blood alcohol concentrations (BAC), but this is a standard model that is used at our center and these concentrations have been replicated over time[6]. The Lieber-DeCarli liquid diet typically results in BAC levels below 150 mg/dL. BACs will range between 100-150 mg/dL during standard chronic feeding[6]​. The alcohol feeding protocol used in this study does induce early-stage alcoholic liver disease (ALD) without binge gavages, but the severity of the disease is less than that with binge gavage. Chronic feeding using the Lieber-DeCarli liquid diet alone results in mild hepatic steatosis and slight elevation of liver enzymes[6]. We have chosen this model to determine the consequences of early ALD because with more severe disease, the confounding effects of multiple organ dysfunction including an increase in intestinal permeability, acute hepatic injury, and consequent cytokine responses could confound interpretation of the effects of ALD. Future studies to evaluate the impact of varying severities of ALD on the liver and skeletal muscle would help determine how the stage and severity of liver disease impact skeletal muscle responses.

**Human tissue samples.** Studies in human subjects were performed following a written informed consent, and were approved by the Institutional Review Board at the Cleveland Clinic and were in conformity with the Helsinki Declaration on human studies. Skeletal muscle samples were obtained from the vastus lateralis muscle from healthy controls (CTL) or humans with alcohol associated cirrhosis (CIR) under IRB 14-1287 and the RNAseq and proteomics data performed and published earlier[7] were used in the present studies for multiomics analyses including vertical/horizontal integration from these published datafiles.

**Method Details**

**Immunoblots.** Immunoblots were generated as previously reported[8]. Briefly, tissue or cellular proteins were extracted, their concentrations measured, separated by gel electrophoresis and electrotransferred to PVDF membranes (Bio-Rad, Hercules, CA, USA). Membranes were incubated with primary antibodies at 1:1000 dilution unless specified, washed in Tris-buffered saline with 0.1% Tween-20, and incubated with appropriate secondary antibodies (1:10,0000 unless specified). Immunoreactivity was detected using a chemiluminescent horseradish peroxidase substrate (Millipore, Billerica, MA, USA), and densitometry quantification was performed using Image J (NIH) as reported earlier[7].

**Hypoxia detection using EF5.** Hypoxia was detected using EF5 as previously described[9]. In brief, myotubes were incubated with 50μM EF5 (generous gift of Cameron Koch, U. Pennsylvania) without/with 100mM ethanol for 6h. Myotubes cultured under hypoxia served as a positive control. EF5 adducts are generated during hypoxia and detected on immunofluorescence using Cy3 conjugated ELK-351 antibody (gift from Cameron Koch). Corrected total fluorescent intensity per cell area was quantified after background subtraction using Image J.

**High Resolution Respirofluorometry.** Standard substrate, uncoupler, inhibitor, titration (SUIT) protocols were utilized to assess mitochondrial function in digitonin-permeabilized hiPSCm as previously described[1]. In brief, complex I substrates (malate, pyruvate, and glutamate), complex II substrate (succinate), and adenosine diphosphate (ADP) were added sequentially. Maximum respiration was then measured in response carbonylcyanide-p-trifluoromethoxyphenylhydrazone (FCCP). Rotenone-sensitive (uncoupled complex I) and rotenone-insensitive (uncoupled complex II) oxygen consumption were then quantified. Response to antimycin A was used to quantify non-mitochondrial residual oxygen consumption. Complex IV dependent oxidation was measured as the difference in oxygen consumption rates in response to sodium azide and tetramethyl phenylene diamine (TMPD) with ascorbate. Calculations were performed using DatLab2 (Oroboros Instruments, Innsbruck, Austria), by recording oxygen concentration and flow rates at 2-second intervals, and corrected for residual oxygen consumption. Oxygen consumption was expressed as pmol.sec^-1^.10-^6^ cells.

**Mitochondrial antioxidants.**

MitoQ (Mitoquinone; MitoQ Limited, Auckland, New Zealand) is a mitochondria-targeted antioxidant designed to selectively accumulate within mitochondria to combat oxidative stress at its source. It is a derivative of the antioxidant coenzyme Q10 (CoQ10) but includes a lipophilic triphenylphosphonium (TPP) cation, which allows it to cross the mitochondrial membrane efficiently. Once inside the mitochondria, MitoQ is reduced to its active form, ubiquinol, where it neutralizes ROS. To investigate the effects of reduction of reactive oxygen species, differentiated C2C12 myotubes were pre-treated with MitoQ, for 1hrs with MitoQ at a final concentration of 200 nM. MitoQ was dissolved in dimethyl sulfoxide (DMSO) and then diluted in culture medium. Control groups received an equivalent volume of DMSO. Treatments were performed for 6 and 24 hours.

MitoTempo (Sigma-Aldrich, St. Louis, MO, USA) is a mitochondria-targeted antioxidant that specifically scavenges superoxide and other reactive oxygen species/free radicals (ROS/FR) within mitochondria. It is a derivative of Tempo (2,2,6,6-tetramethylpiperidine-1-oxyl) and includes a triphenylphosphonium (TPP) cation, which enables it to cross the mitochondrial membrane effectively. Differentiated C2C12 myotubes were pre-treated with MitoTempo for 30 minutes at a final concentration of 25 nM. MitoTempo was dissolved in dimethyl sulfoxide (DMSO) and then diluted in culture medium. Control groups received an equivalent volume of DMSO. Treatments were performed for 6 and 24 hours.

Following treatment, cells were washed with phosphate-buffered saline (PBS) and lysed for subsequent biochemical assays. Protein expression levels of relevant markers were analyzed by Western blotting.

All experiments were conducted in triplicate.

**Myotube diameter.** Differentiated hIPSC-derived myotube diameters were quantified as previously described[10]. Briefly, unstained myotubes were visualized using a light microscope equipped with a camera (Olympus IX2-UCB, Center Valley, PA). Ten random fields were captured within each well of 4-6 biological replicates per group. For quantification, at least 4 random fields were used, analyzing at least 100 myotubes per group. Myotubes without major branch points were selected and diameters were quantified using NIH ImageJ software (imagej.nih.gov/ij/list.html) and expressed as a percentage relative to wild-type controls.

**Mitochondrial morphology**. Mitochondrial morphology was quantified in wild-type myotubes treated with 100mM ethanol for different time points (3-24h) as described by us[9]. In brief, myotubes were stained with MitoTracker Orange (ThermoFisher Scientific, Waltham, MA) and nuclei were counterstained with 4′,6-diamidino-2-phenylindole (DAPI). Images were captured using a Leica TCS-SP8-AOBS inverted confocal microscope equipped with LAS X v3.5.5 software (Leica Microsystems, CMS, GmbH, Wetzlar, Germany). The LAS X software was employed to eliminate bleed-through fluorescent signals using the Dye Separation image processing tool. Two investigators (NW, SD), masked to the treatment group, independently assessed mitochondrial morphology and were categorized as predominantly fused, tubular, intermediate, or fragmented. The proportion of each mitochondrial morphology subtype was normalized to the number of cells.

*3D Electron microscopy (volume electron microscopy)*. Muscle samples were prepared as described in Supplemental Methods.  Image stacks of 300-500 slices were generated at 2.2 kV with 60nm cut thicknesses and 5-7nm/pixel resolution, and were reconstructed and analyzed using ImageJ/FIJI software. Several features in the skeletal muscle from ethanol-fed mice that suggest degenerating mitochondria are present, including swollen, distended mitochondria, and the absence of a mitochondrion in central region. In pair-fed mice, the mitochondria are elongated and parts of the muscle have characteristic mitochondrial and SER organizations that include t-tubule locations. Although in cross sections they appear as paired structures either side of the z-line, the mitochondria span multiple sarcomeres. In most pair-fed muscle samples the mitochondria were of relatively uniform diameter. In ethanol treated animals, however, the muscle fibers were not uniformly affected. In regions where damage was observed, pallid, distended mitochondria with few cristae were common, and in more advanced regions, elongated mitochondria (**Fig 3D**).

**Bioinformatics approaches**

**Multiomic datasets**. Assay for transposase accessible chromatin sequencing (ATACseq), bulk transcriptomics (RNAseq), untargeted global proteomics, phosphoproteomics, and acetylomics were performed using approaches described earlier[11]. We defined a dataset as unbiased data from a molecular layer (ATACseq, RNAseq, total or post-translationally modified proteomics) from a specific model (cell, skeletal muscle from mice or humans).

*Data availability statement*: Untargeted data, including transcriptome and proteomics data have been previously uploaded on public repositories. Data from myotubes treated with 100 mM ethanol for 6 or 24 hours (n=3 biological replicates), and gastrocnemius muscle from mALD or PF mice (n=4 mice in each group) using chronic ethanol feeding with binge as described earlier were used for these analyses[12]. Significance for proteomics and transcriptomics expression levels in cells and tissue was set to p<0.05 as previously reported[12]. Pathway enrichment analysis significance was set at -log(p-value) ≥ 1.3.

**ATACseq.** Computational footprinting was used to assess changes in the activity of a transcription factor during ethanol exposure in C2C12 myotubes as previously reported[7]. In brief, library preparation, sequencing, and analyses were performed with Snakemaker being used for bioinformatics workflow. Differential accessibility analyses were performed using EdgeR Bioconductor package and Footprinting analysis was performed using the HINT tool in the Regulatory Genomics Toolbox (rgt-hint).

**RNAseq.** Total RNA was extracted, evaluated for quality, and used to generate RNA libraries for sequencing as reported by us earlier[7]. The reference genome for the mouse (GRCm38) released by the Genome Reference Consortium in 2012, based on the C57BL/6J Mus musculus strain, was employed. Sequence data have been deposited on GitHub (github.com/atomadam2). Pathway analyses were performed using g:Profiler (KEGG, GO, Reactome) and Qiagen’s Ingenuity Pathway Analysis (IPA) <https://digitalinsights.qiagen.com/IPA>) as previously described[7, 11].

**Proteomics**. Global proteomics analyses were performed in myotubes and muscle tissue as previously described. In brief, proteins were digested with trypsin and untargeted proteomics studies were performed on a Thermo Scientific Fusion Lumos mass spectrometry system (Thermo Scientific, San Jose, CA) by label free quantification and analyzed using MaxQuant[13].

**Phosphoproteomics**. Phosphoproteomics and acetylomics were performed as previously described[2, 11]. In brief, label free proteomics were performed on a Thermo Fisher Fusion Lumos mass spectrometer. Peptide from each sample were phospho-enriched (Thermo Scientific™ High-Select™ TiO2 Phosphopeptide Enrichment Kit), and the eluted peptides Raw mass spectra were searched against the mouse UniProt database using Proteome Discoverer software.

**Acetylomics**. Acetylomics analyses were performed as previously described. In brief, protein extracts were trypsin digested, samples were lyophilized, enriches using PTMSScan acetyl-lysine motif Kit (Cell /Signaling Inc, Danvers, MA). Analyses were done on a ThermoScientific Lumos mass spectrometer for label free quantification using Proteome Discoverer.

**Metabolomics.** Metabolites were extracted from myotubes and subjected to untargeted metabolomics via reversed-phase liquid chromatography (RPLC)/mass spectrometry as previously described. The data were pre-processed via XCMS (platform to analyze metabolomics data) and normalized to protein concentrations. Statistical analyses were performed using MetaboLyzer and MetaboAnalyst. We detected 12,343 and 4,204 total spectral features on RPLC positive and negative ion modes, respectively (**S.Fig. 8**). Of these spectra, we were able to assign putative identifiers to 4615 and 614 features, respectively. The MS/MS spectra of 360 (positive ion mode) and 132 features (negative ion mode) matched unique compounds in the national institute of standards and technology (NIST) spectral library (version 17). Differentially expressed metabolites (DEM) with a significance cutoff of p<0.05 were identified and functional enrichment analyses were performed. Pathways were considered enriched if the p-value<0.05 and the enrichment ratio was computed by Actual/Expected metabolite matches within a pathway.

*Vertical and horizontal integration of datasets*. Following quality assurance measures, upset plots, volcano plots without/with feature extraction and labeling of significant molecules, global and feature extracted heatmaps, functional enrichment analyses using multiple approaches, comparison scatterplots, temporal clustering of responses, and molecular interaction studies using network diagrams were generated using individual dataset unbiased data from the cellular and *in vivo* models as above. Details of these approaches have been previously described[7, 11]. Given the large number of data being analyzed, a combination of supervised and unsupervised approaches with significance levels were defined a priori for datasets.

*Integrated hierarchical scatterplot.* To identify the most DEM across multiple datasets, dot plots were generated based on a hierarchical distribution of the dots (left to right) by the number of unique datasets, the number in the same direction, and the absolute value of the average log fold change. These integrated hierarchical scatterplots (IHSP) allow one to identify critical DEM that are altered with ethanol exposure.

*Upset plots*. Features with the highest average expression across datasets were determined and were compared using UpSet plots. UpSet plots allow for identifying relationships between multiple datasets to identify shared and unique differentially expressed molecules (DEM). Unlike Venn diagrams that also allow for graphical representation of Intersecting (shared) or unique DEMs across datasets, representation becomes challenging when more than 3-4 datasets are included. In contrast, UpSet plots are an efficient approach to depict intersections across a large number of datasets[9].

*Clustering of molecular responses*. We have previously identified temporal clustering of molecular responses to cellular stress[7] and used a similar approach to define clustering of DEMs. Significant changes (increase or decrease in expression) at 6 hours post-ethanol exposure compared to untreated (UnT), but not at 24 hours post-ethanol exposure, were classified as “Early transient” changes. Significant differences in expression between 24 hours and 6 hours post-ethanol exposure, without a difference between either time point and UnT, were termed “Pseudosilent.” A “Late” change was defined as a significant change in expression between UnT and 24 hours post-ethanol exposure, but not at 6 hours post-ethanol exposure. A “persistent” change was defined as a consistent change (increase or decrease in accessibility/expression) at both 6 hours and 24 hours post-ethanol exposure compared to UnT.

Of note, ATACseq analyses did not contain clusters because only one treatment time point was performed—6h ethanol.

Published genesets. Our multiomics datasets were matched against MitoCarta3.0, a comprehensive catalog of mitochondrial localized proteins [14]; CS gene and CellAge for senescence-associated genes; and HIF1α target/signaling pathways from Gene Set Enrichment Analysis (GSEA) as described earlier[2].

A schematic of integrating multiple molecular layers of unbiased datasets followed by experimental validation of critical findings is shown in the **Graphical Abstract**.

*Statistical Approach*

All data are shown as mean±standard deviation unless specified. Quantitative data were compared using Student’s ‘t’ test or analysis of variance with least square difference for post-hoc analyses. For untargeted datasets, significance levels were set to allow up to 8000 DAC/DEG/DEP from each cluster/dataset for enrichment analyses. We therefore varied the significance and fold-change cutoffs for DAC, DEG, and DEP, as previously described[7]. Given the analyses in a comprehensive array of models of in vitro and in vivo ethanol treatment, we set the significance cutoffs within the same range within each dataset from different models. For IHSP, significance cutoff for ATACseq was p<0.005; RNAseq from myotubes p-adjusted<0.05; mouse/human muscle p<0.05; proteomics from myotubes and human muscle p<0.05; mouse muscle p<0.05; phosphoproteomics and acetylomics from myotubes p<0.05.

1. Kumar A, Davuluri G, Welch N, Kim A, Gangadhariah M, Allawy A, et al. Oxidative stress mediates ethanol-induced skeletal muscle mitochondrial dysfunction and dysregulated protein synthesis and autophagy. Free Radic Biol Med. 2019;145:284-99.

2. Mishra S, Welch N, Karthikeyan M, Bellar A, Musich R, Singh SS, et al. Dysregulated cellular redox status during hyperammonemia causes mitochondrial dysfunction and senescence by inhibiting sirtuin-mediated deacetylation. Aging Cell. 2023;22:e13852.

3. Gao B, Xu MJ, Bertola A, Wang H, Zhou Z, Liangpunsakul S. Animal Models of Alcoholic Liver Disease: Pathogenesis and Clinical Relevance. Gene Expr. 2017;17:173-86.

4. Roychowdhury S, McMullen MR, Pisano SG, Liu X, Nagy LE. Absence of receptor interacting protein kinase 3 prevents ethanol-induced liver injury. Hepatology. 2013;57:1773-83.

5. Roychowdhury S, McMullen MR, Pritchard MT, Li W, Salomon RG, Nagy LE. Formation of gamma-ketoaldehyde-protein adducts during ethanol-induced liver injury in mice. Free Radic Biol Med. 2009;47:1526-38.

6. Lamas-Paz A, Hao F, Nelson LJ, Vazquez MT, Canals S, Gomez Del Moral M, et al. Alcoholic liver disease: Utility of animal models. World J Gastroenterol. 2018;24:5063-75.

7. Welch N, Singh SS, Kumar A, Dhruba SR, Mishra S, Sekar J, et al. Integrated multiomics analysis identifies molecular landscape perturbations during hyperammonemia in skeletal muscle and myotubes. J Biol Chem. 2021;297:101023.

8. Kant S, Davuluri G, Alchirazi KA, Welch N, Heit C, Kumar A, et al. Ethanol sensitizes skeletal muscle to ammonia-induced molecular perturbations. J Biol Chem. 2019;294:7231-44.

9. Welch N, Mishra S, Bellar A, Kannan P, Gopan A, Goudarzi M, et al. Differential impact of sex on regulation of skeletal muscle mitochondrial function and protein homeostasis by hypoxia-inducible factor-1alpha in normoxia. J Physiol. 2024;602:2763-806.

10. Attaway AH, Bellar A, Mishra S, Karthikeyan M, Sekar J, Welch N, et al. Adaptive exhaustion during prolonged intermittent hypoxia causes dysregulated skeletal muscle protein homeostasis. J Physiol. 2023;601:567-606.

11. Welch N, Singh SS, Musich R, Mansuri MS, Bellar A, Mishra S, et al. Shared and unique phosphoproteomics responses in skeletal muscle from exercise models and in hyperammonemic myotubes. iScience. 2022;25:105325.

12. Singh SS, Kumar A, Welch N, Sekar J, Mishra S, Bellar A, et al. Multiomics-Identified Intervention to Restore Ethanol-Induced Dysregulated Proteostasis and Secondary Sarcopenia in Alcoholic Liver Disease. Cell Physiol Biochem. 2021;55:91-116.

13. Cox J, Mann M. MaxQuant enables high peptide identification rates, individualized p.p.b.-range mass accuracies and proteome-wide protein quantification. Nat Biotechnol. 2008;26:1367-72.

14. Rath S, Sharma R, Gupta R, Ast T, Chan C, Durham TJ, et al. MitoCarta3.0: an updated mitochondrial proteome now with sub-organelle localization and pathway annotations. Nucleic Acids Res. 2021;49:D1541-D7.
