# Supplementary material for: Integrated Multiomics Analyses of the Molecular Landscape of Sarcopenia in Alcohol‐Related Liver Disease
Source: J Cachexia Sarcopenia Muscle. 2025 Apr 30;16(3):e13818. doi: 10.1002/jcsm.13818 (PMC12044136; doi:10.1002/jcsm.13818)
Supplement: Supplementary file 3 — Table S1 Key reagents [file JCSM-16-e13818-s005.docx]

**S.Table 1**. Key reagents

| **Antibody** | | **Manufacturer** | **Catalog Number; RFID Link** | **Dilution Used** |  |
| --- | --- | --- | --- | --- | --- |
| Alpha-tubulin rabbit pAb | | Cell Signaling Technology, Danvers, MA | 2144 | 1:2000 |  |
| Acetylated-P65-NFkB | | Cell Signaling Technology, Danvers, MA | 3045s | 1:2000 |  |
| Acetylated lysine ab | | Cell Signaling Technology, Danvers, MA | 9441s | 1:2000 |  |
| Anti-mouse IgG, HRP-linked antibody | | Cell Signaling Technology, Danvers, MA | 7076; [AB_330924](http://antibodyregistry.org/AB_330924) | 1:10,000 |  |
| Anti-rabbit IgG, HRP-linked antibody | | Cell Signaling Technology, Danvers, MA | 7074; [AB_2099233](http://antibodyregistry.org/AB_2099233) | 1:10,000 |  |
| ATP5a | | AbCam, Waltham, MA | Ab14748 | 1:2000 |  |
| β-actin (C4) mouse mAb | | Santa Cruz Biotechnology, Santa Cruz, CA | sc-47778; [AB_626632](https://antibodyregistry.org/AB_626632) | 1:10,000 |  |
| DRP1 (D6C7) rabbit mAb | | Cell Signaling Technology, Danvers, MA | 8570s; [AB_10950498](https://antibodyregistry.org/AB_10950498) | 1:2000 |  |
| FIS1 | | Cell Signaling Technology, Danvers, MA | 32525s; | 1:2000 |  |
| HIF-1a (D2U3T) rabbit mAb | | Cell Signaling Technology, Danvers, MA | 14179s; [AB_2622225](https://antibodyregistry.org/AB_2622225) | 1:500 |  |
| HIF-2 alpha/EPAS1 rabbit pAb | | Novus Biologicals, Centennial, CO | NB100-122; [AB_1643862](https://antibodyregistry.org/AB_1643862) | 1:1000 |  |
| HIF-1B/ARNT rabbit mAb | | Cell Signaling Technology, Danvers, MA | 5537; RRID:AB_10694232 | 1:500 |  |
| MFN1 rabbit pAb | | Proteintech Group, Inc., Rosemont, IL | 13798-1-AP; [AB_2266318](https://antibodyregistry.org/AB_2266318) | 1:2000 |  |
| MFN2 rabbit pAb | | Proteintech Group, Inc., Rosemont, IL | 12186-1-AP; [AB_2666320](https://antibodyregistry.org/AB_2666320) | 1:2000 |  |
| MFF rabbit mAb | | Cell Signaling Technology, Danvers, MA | 84580; RRID:AB_2728769) | 1:2000 |  |
| MSTN | | AbCam, Waltham, MA | Ab203076 |  |  |
| OPA1 (D6U6N) Rabbit mAb | | Cell Signaling Technology, Danvers, MA | 80471; [AB_2734117](https://antibodyregistry.org/AB_2734117) | 1:2000 |  |
| phospho-DRP1 (Ser616) rabbit pAb | | Cell Signaling Technology, Danvers, MA | 3455s; [AB_2085352](https://antibodyregistry.org/AB_2085352) | 1:2000 |  |
| phospho-P53 (Ser15) | |  |  |  |  |
| P53 (1C12) mouse mAb | | Cell Signaling Technology, Danvers, MA | 2524s; [AB_331743](https://antibodyregistry.org/AB_331743) | 1:2000 |  |
| phospho-RPS6 (Ser240/244) rabbit pAb | | Cell Signaling Technology, Danvers, MA | 2215s; [AB_331682](https://antibodyregistry.org/AB_331682) | 1:2000 |  |
| P16 (F-4) mouse mAb | | Santa Cruz Biotechnology, Dallas, TX | 74401; [AB_1126945](https://antibodyregistry.org/AB_1126945) | 1:1000 |  |
| P21 rabbit pAb | | Proteintech Group, Inc. (Rosemont, IL) | 10355-1-AP; [AB_2077682](https://antibodyregistry.org/AB_2077682) | 1:1000 |  |
| P65-NFkB | |  |  |  |  |
| Sirt1(D1D7) rabbit aAb | | Cell Signaling Technology, Danvers, MA | 9475a; [AB_2617130](https://antibodyregistry.org/AB_2617130) | 1:2000 |  |
| Sirt2(D4050) rabbit mAb | | Cell Signaling Technology, Danvers, MA | 12650s; [AB_2716762](https://antibodyregistry.org/AB_2716762) | 1:2000 |  |
| Sirt3 rabbit pAb | | AbCam, Waltham, MA | Ab189860 | 1:2000 |  |
| Sirt4 rabbit pAb | | Bio-Vision, Waltham, MA | 3324-30; AB_10974230 | 1:2000 |  |
| Sirt5 (D8C3) rabbit mAb | | Cell Signaling Technology, Danvers, MA | 8782s; [AB_2716763](https://antibodyregistry.org/AB_2716763) | 1:2000 |  |
| Sirt6 (D8D12) rabbit mAb | | Cell Signaling Technology, Danvers, MA | 12486; [AB_2636969](https://antibodyregistry.org/AB_2636969) | 1:2000 |  |
| Sirt7 (D3K5A) rabbit mAb | | Cell Signaling Technology, Danvers, MA | 5360s; [AB_2716764](https://antibodyregistry.org/AB_2716764) | 1:2000 |  |
| TBP | | Cell Signaling Technology, Danvers, MA | 8515 | 1:2000 |  |
| Total OXPHOS Rodent WB Antibody Cocktail mouse mAb | | AbCam, Waltham, MA | ab110413; [AB_2629281](https://antibodyregistry.org/AB_2629281) | 1:1000 |  |
| VDAC1 rabbit pAb | | Cell Signaling Technology, Danvers, MA | 4866s; [AB_2272627](https://antibodyregistry.org/AB_2272627) | 1:2000 |  |
| **Reagent/Software** | **Manufacturer** | | **Catalog Number/Link** | | |
| Amersham ECL Western Blotting Detection Reagents | GE Healthcare Life Sciences, Marlborough, MA | | PRPN2132 | | |
| Immobilon Western Chemiluminescent HRP Substrate | Millipore Sigma, Burlington, MA | | WBKLS0500 | | |
| MitoQ | Selleckchem, Houston, TX | | S8978 | | |
| MitoTEMPO | Millipore Sigma, Burlington, MA | | SML0737 | | |
| MitoTracker Orange CMTMRos | ThermoFisher Thermo Scientific (Waltham, MA) | | M7510 | | |
| NAD/NADH Assay | Promega, Madison, WI | | G9071 | | |
| TransIT®-2020 Transfection Reagent | Mirus Bio LLC (Madison, WI) | | MIR5400 | | |
| GraphPad Prism 9.5.1 | GraphPad (Boston, MA) | | https://www.graphpad.com/ | | |
| ImageJ | National Institutes of Health (Bethesda, MD) | | https://imagej.net/ij/ | | |
| Leica Application Suite X (LAS X) | Leica Microsystems (Morrisville, NC) | | https://www.leica-microsystems.com/products/microscope-software/p/leica-las-x-ls/downloads/ | | |
| MetaboAnalyst | Wishart Research Group, University of Alberta (Edmonton, Alberta, Canada) | | https://www.metaboanalyst.ca/ | | |
| MetaboLyzer | Fornace Lab Informatics, Georgetown University (Washington, D.C.) | | https://sites.google.com/a/georgetown.edu/fornace-lab-informatics/home/metabolyzer | | |
| QIAGEN Ingenuity Pathway Analysis (IPA) | QIAGEN (Germantown, MD) | | https://digitalinsights.qiagen.com/products-overview/discovery-insights-portfolio/analysis-and-visualization/qiagen-ipa/ | | |
| g:Profiler | Eesti Teadusagentuur (Estonian Research Council) and others, Estonia | | https://biit.cs.ut.ee/gprofiler/gost | | |

| **Dataset** | **Project Number/ Database/** | **Project Number/Database/Link** | **Title of dataset** | **Citation** |
| --- | --- | --- | --- | --- |
| C2C12 myotube ethanol-treated ATACseq | SUB14395227; PRJNA1142548 | To be published in SRA upon manuscript  acceptance |  |  |
| C2C12 myotube ATACseq | (Untreated samples) BioProject: PRJNA720403  SRA: SRP313829 | [GEO](https://www.ncbi.nlm.nih.gov/geo/query/acc.cgi?acc=GSE171644) | Integrated molecular landscape perturbations underlie cellular responses during hyperammonemia. | Welch N, et al. *J Biol Chem* 2021 Sep;297(3):101023. PMID: [34343564](https://www.ncbi.nlm.nih.gov/pubmed/34343564) |
| C2C12 myotube ethanol-treated RNAseq | SUB14395227; PRJNA1142548 | [GitHub](https://github.com/atomadam2/Dasarathy_EtOH_C2C12) | Oxidative stress mediates ethanol-induced skeletal muscle mitochondrial dysfunction and dysregulated proteostasis | Kumar et al., 2019 |
| C2C12 myotube ethanol-treated proteomics | To be published upon manuscript acceptance | To be published in PRIDE (proteomeXchange) upon manuscript  acceptance |  | This manuscript |
| C2C12 myotube acetylomics | To be published upon manuscript acceptance | To be published in PRIDE (proteomeXchange) upon manuscript  acceptance |  | This manuscript |
| C2C12 myotube ethanol-treated phosphoproteomics | To be published upon manuscript acceptance | To be published in PRIDE (proteomeXchange) upon manuscript  acceptance |  | This manuscript |
| C2C12 myotube untreated phosphoproteomics | PXD025321 (Untreated samples) | [PRIDE](https://www.ebi.ac.uk/pride/archive/projects/PXD025321) | Hyperammonemia-dependent and independent skeletal muscle | Welch N, et al. *J Biol Chem* 2021 Sep;297(3):101023. PMID: [34343564](https://www.ncbi.nlm.nih.gov/pubmed/34343564) |
| hiPSC myotube RNAseq | SUB14597288, PRJNA1142548 | To be published in SRA upon manuscript  acceptance |  | This manuscript |
| hiPSC myotube proteomics | To be published upon manuscript  acceptance | To be published in PRIDE (proteomeXchange) upon manuscript  acceptance |  | This manuscript |
| Mouse gastrocnemius RNAseq | PRJNA1142548 | SRA | Multiomics-Identified Intervention to Restore Ethanol-Induced Dysregulated Proteostasis and Secondary Sarcopenia in Alcoholic Liver Disease | Singh SS, Kumar A, Welch N, Cell Physiol Biochem. 2021 Feb 6;55(1):91-116. PMID: 33543862. |
| Mouse gastrocnemius proteomics | PXD026955 | [PRIDE](https://www.ebi.ac.uk/pride/archive/projects/PXD026955) | Multiomics-Identified Intervention to Restore Ethanol-Induced Dysregulated Proteostasis and Secondary Sarcopenia in Alcoholic Liver Disease | Singh SS, Kumar A, Welch N, Cell Physiol Biochem. 2021 Feb 6;55(1):91-116. PMID: 33543862. |
| Human gastrocnemius RNAseq (ethanol) | GSE171644 | \| BioProject: PRJNA720403 \| [PRJNA720403](https://www.ncbi.nlm.nih.gov/bioproject/PRJNA720403) \| \| --- \| --- \| \| SRA: SRP313829  https://www.ncbi.nlm.nih.gov/geo/query/acc.cgi?acc=GSE171644 \| [SRP313829](https://www.ncbi.nlm.nih.gov/sra?term=SRP313829) \| | Integrated molecular landscape perturbations underlie cellular responses during hyperammonemia [mouse RNA-seq] | Welch N, et al. *J Biol Chem* 2021 Sep;297(3):101023. PMID: [34343564](https://www.ncbi.nlm.nih.gov/pubmed/34343564) |
| Human gastrocnemius proteomics (ethanol) | PXD031372 | [PRIDE](https://www.ebi.ac.uk/pride/archive/projects/PXD031372) | Metabolic reprogramming during hyperammonemia targets mitochondrial | Dasarathy et al., 2021 |
