# Supplementary material for: Integrated Multiomics Analyses of the Molecular Landscape of Sarcopenia in Alcohol‐Related Liver Disease
Source: J Cachexia Sarcopenia Muscle. 2025 Apr 30;16(3):e13818. doi: 10.1002/jcsm.13818 (PMC12044136; doi:10.1002/jcsm.13818)
Supplement: Supplementary file 4 — Table S2 Appendix of supplementary figure panels [file JCSM-16-e13818-s010.docx]

**S.Table 2.** Appendix of supplementary figure panels

| **Figure** | **Dataset** | **Panels** |
| --- | --- | --- |
| S.Fig 1 (C2C12) | ATACseq | A. Heatmap, B. Volcano plot, C. IPA pathway, D. Functional enrichment |
| S.Fig 2 (C2C12) | RNAseq: Clusters | A. Heatmap, B. Functional enrichment (by cluster), C. ATAC x RNAseq scatterplot IPA pathway, D. Volcano plot, E,F,G,H. Functional enrichment by cluster: E. Early transient, F. Late, G. Persistent, H. Pseudosilent |
| S.Fig 3 (C2C12) | Proteomics: Clusters | A. Heatmap, B. Functional enrichment (by cluster), C. Volcano plot, D,E,F. Functional enrichment by cluster: D. Early transient, E. Late, F. Persistent, G. Pseudosilent |
| S.Fig 4 (hiPSC) | RNAseq: Clusters | A. Heatmap, B. Functional enrichment (by cluster), C. Volcano plot, D,E,F. Functional enrichment by cluster: D. Early transient, E. Late, F. Persistent, G. Pseudosilent |
| S.Fig 5 (hiPSC) | Proteomics: Clusters | A. Heatmap, B. Functional enrichment (by cluster), C. Volcano plot, D,E,F. Functional enrichment by cluster: D. Early transient, E. Late, F. Persistent, G. Pseudosilent |
| S.Fig 6 (C2C12) | Acetylome: Clusters | A. Heatmap, B. Functional enrichment (by cluster), C. Volcano plot, D,E,F. Functional enrichment by cluster: D. Early transient, E. Late, F. Persistent, G. Pseudosilent |
| S.Fig 7 (C2C12) | Phosphoproteome: Clusters | A. Heatmap, B. Functional enrichment (by cluster), C. Volcano plot, D,E,F. Functional enrichment by cluster: D. Early transient, E. Late, F. Persistent, G. Pseudosilent |
| S.Fig 8 (C2C12) | Metabolomics | 1. Heatmaps, B. Impact dot plots, C. Horizontal Integration of metabolomics and proteomic |
| S.Fig 9 (mouse) | Mouse omics | A. Mouse RNAseq: Functional enrichment, B. Mouse proteome: Functional enrichment |
| S.Fig 10 (human) | Human omics | A. Human RNAseq: Functional enrichment, B. Human proteome: Functional enrichment |
| S.Fig 11 | Overall cluster IPA comparison analysis | A. Persistent, B. Pseudosilent |
| S.Fig 12 | Targeted HISP: hierarchical integrated scatter plots | A. Senescence, B. TCA cycle, C. MitoCarta3.0, D. HIF1α signaling |
| S.Fig 13 (hiPSC) | Sirtuin expression in hiPSC | Representative densitometries and immunoblots of Sirtuins 1-7 in hiPSC. |
| S.Fig 14 | Genelist heatmaps | Heatmaps for A. HIF1a signaling; B. Proteomics; C. Circadian Rhythm |
