# Supplementary material for: Integrated Multiomics Analyses of the Molecular Landscape of Sarcopenia in Alcohol‐Related Liver Disease
Source: J Cachexia Sarcopenia Muscle. 2025 Apr 30;16(3):e13818. doi: 10.1002/jcsm.13818 (PMC12044136; doi:10.1002/jcsm.13818)
Supplement: Supplementary file 5 — Table S3 Supplementary table appendix [file JCSM-16-e13818-s003.docx]

**S.Table 3**. Supplementary Table appendix

| **Name** | **Title** | **File type** | **Figure/panel it supports** |
| --- | --- | --- | --- |
| S.Table 1 | Key Reagents | .docx | Supplementary Methods section |
| S.Table 2 | Appendix of S.Fig panels | .docx | Supplementary Methods section |
| S.Table 3 | Appendix of S.Tables | .docx | Supplementary Methods section |
| S.Table 4 | Overall UpSet Plot molecules | .csv | Fig 1A |
| S.Table 5 | Enrichment by dataset and cluster |  | Fig 1C |
| S.Table 6 | Shared and unique cluster enrichment |  | Fig 1D |
| S.Table 7 | Metabolite pathways by cluster |  | Fig 1F |
| S.Table 8 | Metabolites grouped by topic |  | Fig 1F |
| S.Table 9 | Mitochondrial processes by cluster |  | Fig 3 |
| S.Table 10 | Proteomics vs Metabolites – TCA cycle |  | Fig 3 |
| S.Table 11 | Senescence processes by cluster |  | Fig 7 |
