# Supplementary material for: Integrated Multiomics Analyses of the Molecular Landscape of Sarcopenia in Alcohol‐Related Liver Disease
Source: J Cachexia Sarcopenia Muscle. 2025 Apr 30;16(3):e13818. doi: 10.1002/jcsm.13818 (PMC12044136; doi:10.1002/jcsm.13818)
Supplement: Supplementary file 7 — Table S5 Enriched pathways (by dataset, by cluster) in molecules with increased or decreased differential expression/modification [file JCSM-16-e13818-s009.docx]

**S.Table 5**. Enriched pathways (by dataset, by cluster) in molecules with increased or decreased differential expression/modification

| **Dataset** | **Cluster** | **Increased Processes** | **Decreased Processes** |
| --- | --- | --- | --- |
| **Cell Proteomics** | Early Transient | Pyruvate metabolism (ATP-related), Detoxification of hydrogen peroxide (Stress response), Structural molecule activity (Chromatin regulation), Mitochondrial inner membrane (Mitochondria) | Enzyme binding (Chromatin regulation), Protein-containing complex, Small ribosomal subunit, Melanosome |
|  | Late | Proton motive force-driven mitochondrial ATP synthesis (ATP-related), Structural constituent of ribosome (Cell cycle), Translation at presynapse/postsynapse (Chromatin regulation), Inner mitochondrial membrane protein complex | Ribosomal small subunit biogenesis (Cell cycle), Negative regulation of transmembrane transport, Troponin complex, Actin filament binding |
|  | Persistent | Phenylalanine metabolism (ATP-related), Translation at presynapse/postsynapse (Chromatin regulation), Cytosolic large ribosomal subunit (Cell cycle) | Ribosomal large subunit biogenesis (Cell cycle), Cytosolic large ribosomal subunit (Chromatin regulation) |
| **Cell Phosphoproteomics** | Early Transient | ATP binding (ATP-related), ATP-dependent chromatin remodeling (Chromatin regulation), Focal adhesion (Stress response), Sister chromatid segregation (Cell cycle), Insulin signaling pathway (Mitochondria) | Chromatin binding (Chromatin regulation), Transcription corepressor activity, Actin filament binding (Cytoskeleton), Apoptotic execution phase (Stress response) |
|  | Late | ATP binding (ATP-related), Protein kinase binding (Chromatin regulation), Ubiquitin-mediated proteolysis (Stress response), Regulation of cell cycle processes (Cell cycle), TP53 activity regulation (Mitochondria) | Negative regulation of transcription by RNA polymerase II (Chromatin regulation), Actin filament binding (Cytoskeleton), Signaling by Rho GTPases (Stress response), Apoptotic execution phase |
|  | Persistent | ATP binding (ATP-related), Chromatin DNA binding (Chromatin regulation), Positive regulation of cell migration (Stress response), Regulation of cell cycle (Cell cycle) | Negative regulation of transcription by RNA polymerase II (Chromatin regulation), Actin filament binding (Cytoskeleton), Signaling by Rho GTPases (Stress response), RNA metabolism |
|  | Pseudosilent | Regulation of actin cytoskeleton (Cytoskeleton), Positive regulation of molecular function (Chromatin regulation), Actin binding (Cytoskeleton), Regulation of cell communication (Stress response) | Negative regulation of cellular process (Cell cycle), Protein-macromolecule adaptor activity (Chromatin regulation), Cytoskeleton organization, RNA polymerase II complex binding |
| **Cell Acetylomics** | Early Transient | TCA cycle (ATP-related), Pyruvate metabolism (ATP-related), Chromatin remodeling (Chromatin regulation), Positive regulation of transcription by RNA polymerase II (Chromatin regulation), Aerobic respiration (Mitochondria) | Enzyme binding (Chromatin regulation), Protein-containing complex binding (Chromatin regulation), ADP binding, Transcription coregulator activity (Chromatin regulation) |
|  | Late | Carbon metabolism (ATP-related), Mitochondrial ATP transmembrane transport (Mitochondria), Positive regulation of DNA metabolic process (Chromatin regulation), Cytoplasmic translation (Cell cycle) | Protein binding (Chromatin regulation), RNA processing (RNA metabolism), Actin filament binding (Cytoskeleton), Protein-macromolecule adaptor activity |
|  | Persistent | RNA degradation (RNA metabolism), ATP-dependent activity (ATP-related), Positive regulation of gene expression (Chromatin regulation), Endocytosis (Cell cycle) | Actin filament binding (Cytoskeleton), Protein complex binding (Chromatin regulation), Phospholipase A2 inhibitor activity (Chromatin regulation), Cytoskeleton organization |
|  | Pseudosilent | Cytoplasmic translation (Cell cycle), Chromatin remodeling (Chromatin regulation), Cellular response to stress (Stress response), Positive regulation of macromolecule biosynthetic process (Chromatin regulation) | Protein dimerization activity (Chromatin regulation), rRNA binding (RNA metabolism), Nonsense-mediated decay (Protein synthesis), SRP-dependent cotranslational protein targeting to membrane |
| **Cell RNAseq** | Early Transient | Chromatin remodeling (Chromatin regulation), Regulation of transcription by RNA polymerase II (Chromatin regulation), Cellular response to stress (Stress response), ATP binding (ATP-related), mRNA binding (RNA metabolism) | Protein-containing complex binding (Chromatin regulation), Hydrolase activity (Chromatin regulation), Protein transmembrane transporter activity (Cell cycle), Endonuclease activity (RNA metabolism) |
|  | Late | Metabolic pathways (ATP-related), Oxidative phosphorylation (ATP-related), Positive regulation of protein localization (Chromatin regulation), mRNA processing and splicing (RNA metabolism) | Actin filament binding (Cytoskeleton), Cytoskeletal protein binding (Cytoskeleton), Phosphorylation (Cell cycle), Intracellular transport (Cell cycle) |
|  | Persistent | Metabolic pathways (ATP-related), DNA replication (Chromatin regulation), Positive regulation of gene expression (Chromatin regulation), Protein ubiquitination (Chromatin regulation) | Negative regulation of transcription by RNA polymerase II (Chromatin regulation), Membrane organization (Cytoskeleton), Extracellular matrix organization (Cytoskeleton), Collagen biosynthesis (Cytoskeleton) |
|  | Pseudosilent | Positive regulation of DNA-templated transcription (Chromatin regulation), Nuclear inner membrane organization (Chromatin regulation) | Negative regulation of asymmetric cell division (Cell cycle) |
| **hiPSC Proteomics** | Early Transient | Autophagy (Stress response), Ribosome function (Cell cycle), ATP binding (ATP-related), Protein serine kinase activity (Chromatin regulation), Protein phosphorylation (Stress response) | Negative regulation of cytoskeleton organization (Cytoskeleton), Endocytosis (Cell cycle), Dendrite development (Cell cycle), Spindle assembly (Cell cycle) |
|  | Late | Metabolic pathways (ATP-related), Glutathione metabolism (Stress response), ATP-dependent chromatin remodeling (Chromatin regulation), Organelle organization (Cell cycle) | Ribosomal large subunit biogenesis (Cell cycle), Catabolic processes (Cell cycle), rRNA processing (RNA metabolism), Apoptosis (Cell cycle) |
|  | Persistent | rRNA processing (RNA metabolism), Ribosomal biogenesis (Cell cycle), Cellular response to stress (Stress response), Intracellular protein transport (Cell cycle) | Cellular starvation response (Stress response), Viral mRNA translation (RNA metabolism) |
|  | Pseudosilent | Protein transporter activity (Cell cycle), Enzyme regulator activity (Chromatin regulation), Cardiac muscle cell differentiation (Cell cycle), Organelle organization (Cytoskeleton) | Antiviral mechanisms (Immune response), Signaling by VEGF (Stress response), VEGFA-VEGFR2 Pathway (Cell cycle), Downstream signal transduction (Cell cycle) |
| **hiPSC RNAseq** | Early Transient | Cytoplasmic translation (Cell cycle), Negative regulation of protein modification (Protein metabolic process), Ribosomal large subunit biogenesis (Cell cycle), Mitochondrial transport (Mitochondria) | Proteasome degradation (Protein turnover), Cholesterol metabolism (Lipid biosynthesis) |
|  | Late | Proteasome degradation (Protein turnover), Cholesterol biosynthesis pathway (Lipid metabolism), G1 to S phase transition (Cell cycle), Mitochondrial transport (Mitochondria) | Actin filament binding (Cytoskeleton), Cadherin binding (Cell adhesion) |
|  | Persistent | Positive regulation of transcription by RNA polymerase II (Chromatin regulation), Ribosome biogenesis (Protein synthesis), Mitochondrial transport (Mitochondria), Oxidoreductase activity (Mitochondria) | Proteasome degradation (Protein turnover), Structural constituent of cytoskeleton (Cytoskeleton) |
|  | Pseudosilent | ATP binding (ATP-related), Mitotic sister chromatid segregation (Cell cycle), Protein modification (post-translational modifications), Protein localization to organelle (Intracellular transport) | Mitochondrial protein-containing complex binding (Mitochondria), Oxidoreductase activity (Mitochondria), VEGFA-VEGFR2 signaling (Angiogenesis) |
