# Supplementary material for: Integrated Multiomics Analyses of the Molecular Landscape of Sarcopenia in Alcohol‐Related Liver Disease
Source: J Cachexia Sarcopenia Muscle. 2025 Apr 30;16(3):e13818. doi: 10.1002/jcsm.13818 (PMC12044136; doi:10.1002/jcsm.13818)
Supplement: Supplementary file 8 — Table S6 Shared processes (by cluster) with increased or decreased gene expression/modification [file JCSM-16-e13818-s011.docx]

**S.Table 6. Shared processes (by cluster) with increased or decreased gene expression/modification**

| **Cluster** | **Increased Processes** | **Decreased Processes** |
| --- | --- | --- |
| **Early Transient** | - ATP binding (hiPSC RNAseq, Cell Proteomics, Cell Acetylomics, hiPSC Proteomics) - Chromatin remodeling/structural molecule activity (Cell Acetylomics, hiPSC RNAseq, Cell Proteomics) - Mitochondrial function/transport (hiPSC RNAseq, hiPSC Proteomics, Cell Proteomics) | Protein degradation/turnover: (hiPSC RNAseq, Cell Proteomics) |
| **Late** | - Proteasome degradation (hiPSC RNAseq) - Mitochondrial function/transport (hiPSC RNAseq, hiPSC Proteomics, Cell Acetylomics) | Structural reorganization (Actin filament binding): (hiPSC RNAseq, Cell Proteomics, Cell Acetylomics) |
| **Persistent** | - Positive regulation of transcription by RNA polymerase II (hiPSC RNAseq, Cell Acetylomics) - Ribosome biogenesis/protein synthesis (hiPSC RNAseq, hiPSC Proteomics, Cell Acetylomics) - Mitochondrial function/transport (hiPSC RNAseq) | Protein degradation/turnover (Proteasome degradation): (hiPSC RNAseq, Cell Acetylomics) |
| **Pseudosilent** | - ATP binding (hiPSC RNAseq, Cell Acetylomics, hiPSC Proteomics) - Cytoplasmic translation (Cell Acetylomics, hiPSC RNAseq) - Protein modification/activity (hiPSC RNAseq, hiPSC Proteomics) | Mitochondrial interactions/metabolism (hiPSC RNAseq, Cell Acetylomics) |
