# Supplementary material for: Integrated Multiomics Analyses of the Molecular Landscape of Sarcopenia in Alcohol‐Related Liver Disease
Source: J Cachexia Sarcopenia Muscle. 2025 Apr 30;16(3):e13818. doi: 10.1002/jcsm.13818 (PMC12044136; doi:10.1002/jcsm.13818)
Supplement: Supplementary file 9 — Table S7 Enriched pathways (by cluster) in metabolites that are either increased or decreased in expression from untargeted metabolomics [file JCSM-16-e13818-s001.docx]

**S.Table 7.** Enriched pathways (by cluster) in metabolites that are either increased or decreased in expression from untargeted metabolomics

| **Category** | **Decrease** | **Increase** |
| --- | --- | --- |
| Early Transient | Steroid hormone biosynthesis, Taurine and hypotaurine metabolism, Drug metabolism - cytochrome P450, Primary bile acid biosynthesis, Valine, leucine and isoleucine biosynthesis | Steroid biosynthesis |
| Late | Histidine metabolism, beta-Alanine metabolism, Alanine, aspartate and glutamate metabolism, Glycine, serine and threonine metabolism, Arginine biosynthesis, Metabolism of xenobiotics by cytochrome P450 | Pentose phosphate pathway |
| Persistent | Phenylalanine, tyrosine and tryptophan biosynthesis, Arginine biosynthesis, Arginine and proline metabolism, Phenylalanine metabolism, Tyrosine metabolism, Glycerolipid metabolism, Tryptophan metabolism | Drug metabolism - cytochrome P450, Glyoxylate and dicarboxylate metabolism, Pyrimidine metabolism, Citrate cycle (TCA cycle) |
| Pseudosilent | NaN | Vitamin B6 metabolism |
