# Supplementary material for: Integrated Multiomics Analyses of the Molecular Landscape of Sarcopenia in Alcohol‐Related Liver Disease
Source: J Cachexia Sarcopenia Muscle. 2025 Apr 30;16(3):e13818. doi: 10.1002/jcsm.13818 (PMC12044136; doi:10.1002/jcsm.13818)
Supplement: Supplementary file 10 — Table S8 Summary of shared pathways among metabolomics clusters [file JCSM-16-e13818-s014.docx]

**S.Table 8**. Summary of Shared Pathways Among Metabolomics Clusters

| **Pathway** | **Early Transient** | **Late** | **Persistent** | **Pseudosilent** |
| --- | --- | --- | --- | --- |
| **Glycine, Serine, and Threonine Metabolism** | Increased | Increased | Increased |  |
| **Histidine Metabolism** | Increased | Increased |  |  |
| **Alanine, Aspartate, and Glutamate Metabolism** |  | Increased | Increased |  |
| **Arginine and Proline Metabolism** | Increased |  |  |  |
| **Cysteine and Methionine Metabolism** |  |  | Increased |  |
| **Vitamin B6 Metabolism** |  |  |  | Increased (related to amino acids) |
| **Energy Production** | TCA Cycle (Increased)  Pyruvate Metabolism (Increased) | - | Citrate Cycle (TCA Cycle) (Increased) |  |
| **Drug Metabolism and Detoxification** | - | Metabolism of Xenobiotics by Cytochrome P450 (Increased) | Drug Metabolism Cytochrome P450 (Increased & Decreased) | Drug Metabolism - Other Enzymes (Increased)  Metabolism of Xenobiotics by Cytochrome P450 (Increased) |
| **Steroid Biosynthesis and Hormone Metabolism** | Steroid Biosynthesis (Increased) | Steroid Biosynthesis (Decreased) | Steroid Hormone Biosynthesis (Decreased) | Steroid Hormone Biosynthesis (Increased) |
| **Nucleotide Metabolism** | Pyrimidine Metabolism (Increased)  Purine Metabolism (Increased) |  | Pyrimidine Metabolism (Increased) |  |
| **Fatty Acid and Lipid Metabolism** | Linoleic Acid Metabolism (Decreased) | Fatty Acid Degradation (Decreased) |  |  |
| **Glucose and Carbohydrate Metabolism** | Starch and Sucrose Metabolism (Decreased) | Glycolysis/Gluconeogenesis (Decreased) |  |  |
| **Sulfur-Containing Compound Metabolism** | Taurine and Hypotaurine Metabolism (Decreased) |  | Cysteine and Methionine Metabolism (Increased) |  |
