# Supplementary material for: Integrated Multiomics Analyses of the Molecular Landscape of Sarcopenia in Alcohol‐Related Liver Disease
Source: J Cachexia Sarcopenia Muscle. 2025 Apr 30;16(3):e13818. doi: 10.1002/jcsm.13818 (PMC12044136; doi:10.1002/jcsm.13818)
Supplement: Supplementary file 11 — Table S9 DAVID pathways (by cluster) related mitochondria [file JCSM-16-e13818-s012.docx]

**S.Table 9. DAVID pathways (by cluster) related mitochondria**

| **Cluster** | **Increased Processes** | **Decreased Processes** |
| --- | --- | --- |
| **Early Transient** | - Mitochondrial inner membrane (Cell Proteomics) - Mitochondrial protein-containing complex (Cell Acetylomics) - Mitochondrial function/transport (hiPSC RNAseq, hiPSC Proteomics, Cell Proteomics) |  |
| **Late** | - Mitochondrial function/transport (hiPSC RNAseq, hiPSC Proteomics, Cell Acetylomics) |  |
| **Persistent** | - Mitochondrial function/transport (hiPSC RNAseq) |  |
| **Pseudosilent** | - Mitochondrial function/transport (hiPSC RNAseq, Cell Acetylomics) |  |
