# Supplementary material for: Integrated Multiomics Analyses of the Molecular Landscape of Sarcopenia in Alcohol‐Related Liver Disease
Source: J Cachexia Sarcopenia Muscle. 2025 Apr 30;16(3):e13818. doi: 10.1002/jcsm.13818 (PMC12044136; doi:10.1002/jcsm.13818)
Supplement: Supplementary file 12 — Table S10 Comparative analysis of tri‐carboxylic acid (TCA) cycle processes in proteomics datasets. DEM: differentially expressed metabolites, DEP: differentially expressed proteins [file JCSM-16-e13818-s004.docx]

**S.Table 10**. Comparative analysis of tri-carboxylic acid (TCA) cycle processes in proteomics datasets

| **Cluster** | **hiPSC Proteomics** | **C2C12 Proteomics** | **Human Proteomics** | **Metabolomics** |
| --- | --- | --- | --- | --- |
| **Early Transient** | ATP binding, mitochondrial function (increased DEP) | Pyruvate metabolism, mitochondrial function (Increased DEP) | Citrate cycle, tricarboxylic acid cycle, proton motive force-driven mitochondrial ATP synthesis​​ (increased DEP) | TCA Cycle, Pyruvate Metabolism (increased DEM) |
| **Late** | Metabolic pathways (increased DEP) | Metabolic pathways (Increased DEP) |  |  |
| **Persistent** |  |  |  | Citrate Cycle (TCA Cycle) (increased DEM) |
| **Pseudosilent** |  |  |  |  |

DEM: Differentially expressed metabolites, DEP: Differentially expressed proteins
