# Supplementary material for: Integrated Multiomics Analyses of the Molecular Landscape of Sarcopenia in Alcohol‐Related Liver Disease
Source: J Cachexia Sarcopenia Muscle. 2025 Apr 30;16(3):e13818. doi: 10.1002/jcsm.13818 (PMC12044136; doi:10.1002/jcsm.13818)
Supplement: Supplementary file 13 — Table S11 DAVID pathways (by cluster) related to cell cycle, cell damage and senescence [file JCSM-16-e13818-s006.docx]

### S.Table 11. DAVID pathways (by cluster) related to Cell Cycle, Cell Damage, and Senescence

| **Cluster** | **Increased Processes** | **Decreased Processes** |
| --- | --- | --- |
| **Early Transient** | - Cell cycle (Cell Phosphoproteomics, Cell RNAseq)​​​​ - Response to stress (Cell RNAseq, hiPSC Proteomics)​​​​ | - Protein degradation/turnover (hiPSC RNAseq, Cell Proteomics)​​​​ - HIF-1 signaling pathway (Cell Acetylomics)​​ |
| **Late** | - Cell cycle (hiPSC RNAseq)​​​​ - Cellular response to hypoxia (hiPSC RNAseq)​​ | - Structural reorganization (Actin filament binding) (hiPSC RNAseq, Cell Proteomics, Cell Acetylomics)​​​​​​ |
| **Persistent** | - Positive regulation of transcription by RNA polymerase II (hiPSC RNAseq, Cell Acetylomics)​​​​ - Ribosome biogenesis/protein synthesis (hiPSC RNAseq, hiPSC Proteomics, Cell Acetylomics)​​​​​​ - Mitochondrial function/transport (hiPSC RNAseq)​​ - Regulation of cellular response to stress (Cell Phosphoproteomics)​​ | - Protein degradation/turnover (Proteasome degradation) (hiPSC RNAseq, Cell Acetylomics)​​​​ |
| **Pseudosilent** | - ATP binding (hiPSC RNAseq, Cell Acetylomics, hiPSC Proteomics)​​​​​​ - Cytoplasmic translation (Cell Acetylomics, hiPSC RNAseq)​​​​ - Protein modification/activity (hiPSC RNAseq, hiPSC Proteomics)​​​​ - Cell cycle processes (hiPSC RNAseq)​​ - Cellular response to stress (Cell Acetylomics)​​ | - Mitochondrial interactions/metabolism (hiPSC RNAseq, Cell Acetylomics)​​​​ |
