# Supplementary material for: Integrated Multiomics Analyses of the Molecular Landscape of Sarcopenia in Alcohol‐Related Liver Disease
Source: J Cachexia Sarcopenia Muscle. 2025 Apr 30;16(3):e13818. doi: 10.1002/jcsm.13818 (PMC12044136; doi:10.1002/jcsm.13818)
Supplement: Supplementary file 14 — Figure S1 C2C12 assay for transposase accessible chromatin (ATACseq): QC and overall heatmap. C2C12 myotubes were differentiated and either not treated (UnT) or treated with 100 mM ethanol (EtOH) for 6 h. ATACseq was performed, and differentially accessible areas of the chromatin (DAC) were analysed. (A) Heatmap of DAC. (B) Functional enrichment study showing pathways derived from Ingenuity Pathway Analysis (IPA, QIAGEN Inc). (C) Volcano plot highlighting 25 most changed DAC (orange). (D) Functional enrichment using Gene Ontology (GO): Biological Process (BP), GO: Molecular Function (MF) and Kyoto Encyclopaedia of Genes and Genomes (KEGG). Functional enrichment analysis is used to identify biological processes, pathways or molecular functions that are overrepresented in a set of genes or proteins compared to a background set. The most enriched pathways were shown. Significance for ATACseq taken at p < 0.005. Figure S2 C2C12 bulk RNA sequencing (RNAseq) cluster heatmaps, pathways and volcano plots. C2C12 myotubes were differentiated and either not treated (UnT) or treated with 100 mM ethanol (EtOH) for 6 or 24 h. RNAseq was performed. Differentially expressed molecules (DEMs) were clustered into early transient (changed at 6 h without change at 24 h EtOH), late (unchanged at 6 h but changed at 24 h), persistent (sustained increase or decrease in expression in the same direction at both 6 and 24 h) and pseudosilent (significant change in expression between 6 and 24 h of EtOH treatment, but not significantly different expression at either treatment timepoint from untreated myotubes) and analysed. (A) Cluster heatmaps. (B) Cluster functional enrichment using Ingenuity Pathway Analysis (IPA, QIAGEN Inc). (C) Scatter plot comparing differentially accessible areas of the chromatin using assay for transposase accessible chromatin (ATACseq) and DEM on RNAseq. (D) Cluster volcano plots highlighting the 25 most changed DEM (orange). (E–G) Cluster functional enrichment using g: [file JCSM-16-e13818-s013.pdf]

S. Fig 1

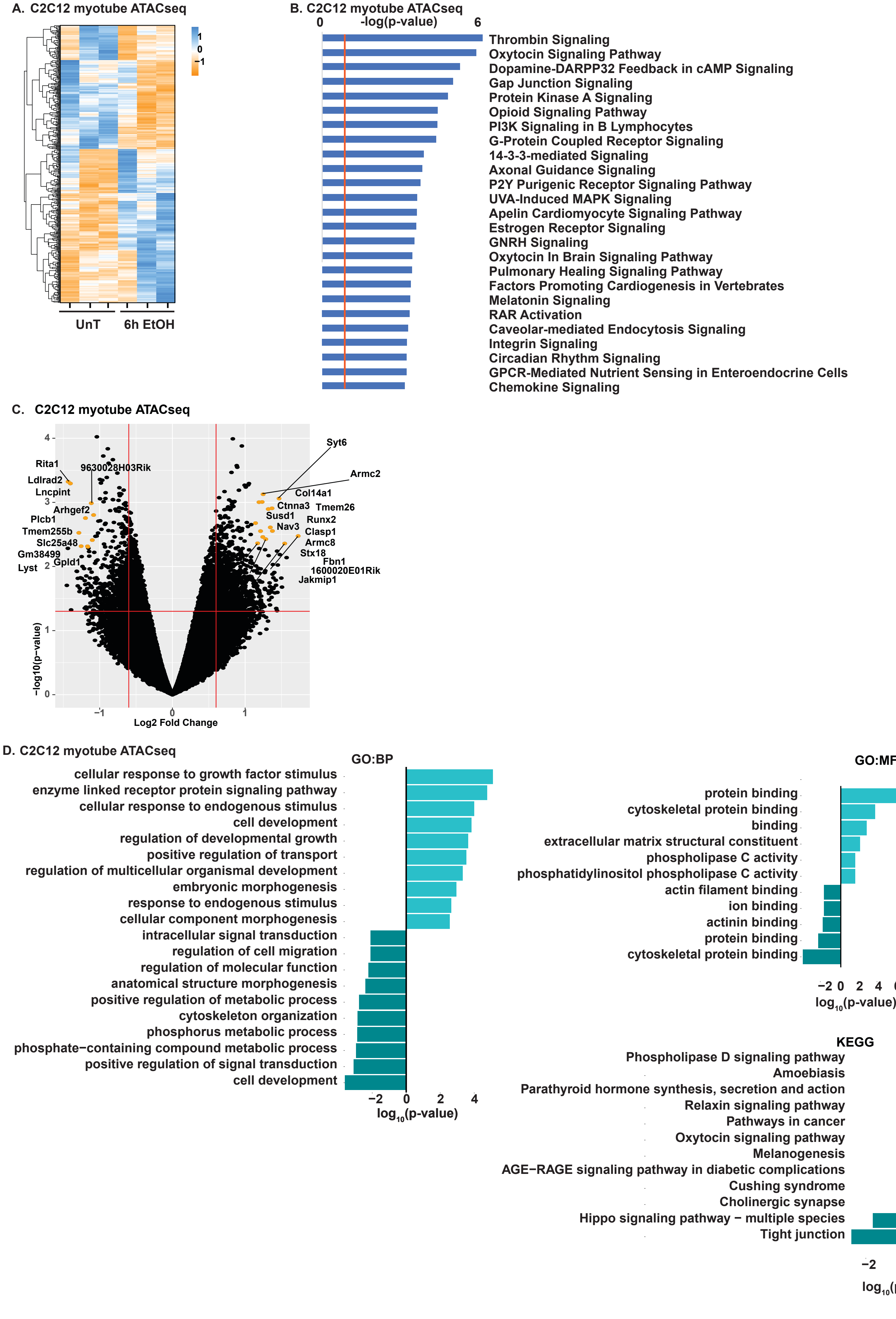
A. C2C12 myotube ATACseq
1
0
−1
UnT
6h EtOH
B. C2C12 myotube ATACseq
0
-log(p-value)
6
Thrombin Signaling
Oxytocin Signaling Pathway
Dopamine-DARPP32 Feedback in cAMP Signaling
Gap Junction Signaling
Protein Kinase A Signaling
Opioid Signaling Pathway
PI3K Signaling in B Lymphocytes
G-Protein Coupled Receptor Signaling
14-3-3-mediated Signaling
Axonal Guidance Signaling
P2Y Purigenic Receptor Signaling Pathway
UVA-Induced MAPK Signaling
Apelin Cardiomyocyte Signaling Pathway
Estrogen Receptor Signaling
GNRH Signaling
Oxytocin In Brain Signaling Pathway
Pulmonary Healing Signaling Pathway
Factors Promoting Cardiogenesis in Vertebrates
Melatonin Signaling
RAR Activation
Caveolar-mediated Endocytosis Signaling
Integrin Signaling
Circadian Rhythm Signaling
GPCR-Mediated Nutrient Sensing in Enteroendocrine Cells
Chemokine Signaling
C. C2C12 myotube ATACseq
Rita1
9630028H03Rik
Ldlrad2
Lncpint
Arhgef2
Plcb1
Tmem255b
Slc25a48
Gm38499
Gpld1
Lyst
Syt6
Armc2
Col14a1
Ctnna3
Tmem26
Susd1
Runx2
Nav3
Clasp1
Armc8
Stx18
Fbn1
1600020E01Rik
Jakmip1
−log10(p−value)
Log2 Fold Change
D. C2C12 myotube ATACseq
GO:BP
cellular response to growth factor stimulus
enzyme linked receptor protein signaling pathway
cellular response to endogenous stimulus
cell development
regulation of developmental growth
positive regulation of transport
regulation of multicellular organismal development
embryonic morphogenesis
response to endogenous stimulus
cellular component morphogenesis
intracellular signal transduction
regulation of cell migration
regulation of molecular function
anatomical structure morphogenesis
positive regulation of metabolic process
cytoskeleton organization
phosphorus metabolic process
phosphate−containing compound metabolic process
positive regulation of signal transduction
cell development
log10(p-value)
GO:MF
protein binding
cytoskeletal protein binding
binding
extracellular matrix structural constituent
phospholipase C activity
phosphatidylinositol phospholipase C activity
actin filament binding
ion binding
actinin binding
protein binding
cytoskeletal protein binding
log10(p-value)
KEGG
Phospholipase D signaling pathway
Amoebiasis
Parathyroid hormone synthesis, secretion and action
Relaxin signaling pathway
Pathways in cancer
Oxytocin signaling pathway
Melanogenesis
AGE−RAGE signaling pathway in diabetic complications
Cushing syndrome
Cholinergic synapse
Hippo signaling pathway − multiple species
Tight junction
log10(p-value)

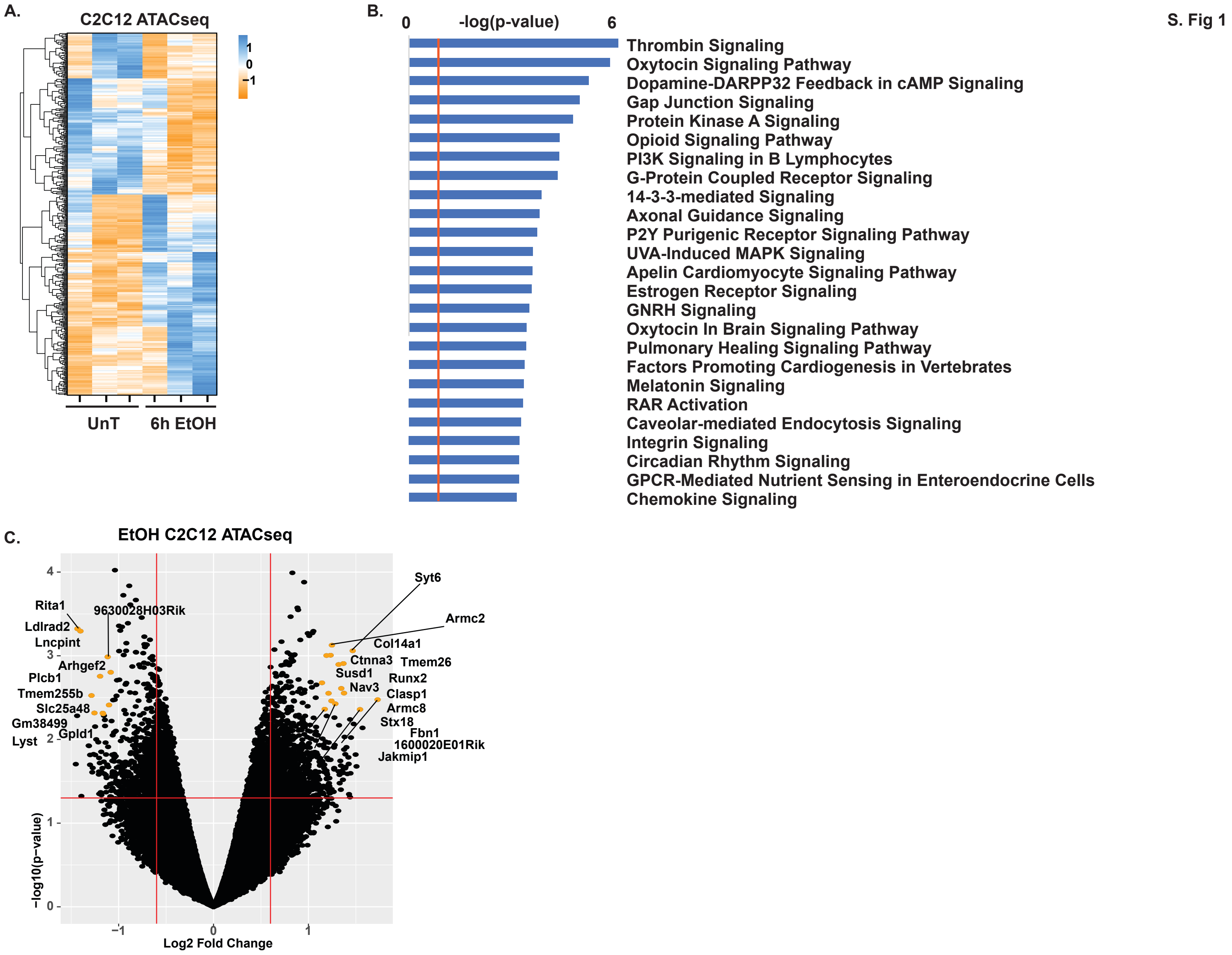
S. Fig 1
A.
C2C12 ATACseq
1
0
−1
UnT
6h EtOH
B.
0
-log(p-value)
6
Thrombin Signaling
Oxytocin Signaling Pathway
Dopamine-DARPP32 Feedback in cAMP Signaling
Gap Junction Signaling
Protein Kinase A Signaling
Opioid Signaling Pathway
PI3K Signaling in B Lymphocytes
G-Protein Coupled Receptor Signaling
14-3-3-mediated Signaling
Axonal Guidance Signaling
P2Y Purigenic Receptor Signaling Pathway
UVA-Induced MAPK Signaling
Apelin Cardiomyocyte Signaling Pathway
Estrogen Receptor Signaling
GNRH Signaling
Oxytocin In Brain Signaling Pathway
Pulmonary Healing Signaling Pathway
Factors Promoting Cardiogenesis in Vertebrates
Melatonin Signaling
RAR Activation
Caveolar-mediated Endocytosis Signaling
Integrin Signaling
Circadian Rhythm Signaling
GPCR-Mediated Nutrient Sensing in Enteroendocrine Cells
Chemokine Signaling
C.
EtOH C2C12 ATACseq
Rita1
9630028H03Rik
Ldlrad2
Lncpint
Arhgef2
Plcb1
Tmem255b
Slc25a48
Gm38499
Gpld1
Lyst
Syt6
Armc2
Col14a1
Ctnna3
Tmem26
Susd1
Runx2
Nav3
Clasp1
Armc8
Stx18
Fbn1
1600020E01Rik
Jakmip1
−log10(p−value)
Log2 Fold Change

D. C2C12 myotube ATACseq

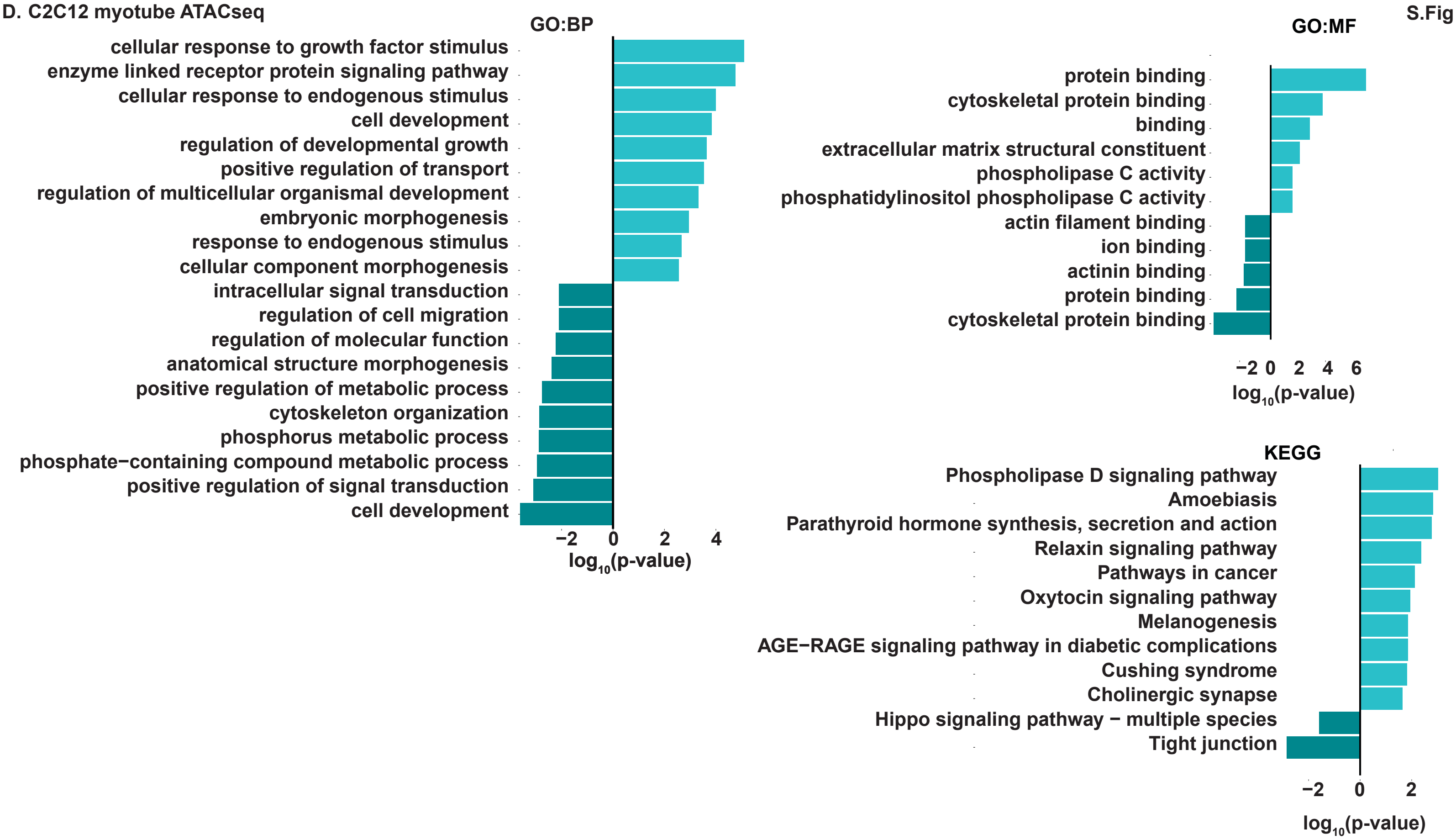

A. C2C12 myotube RNAseq

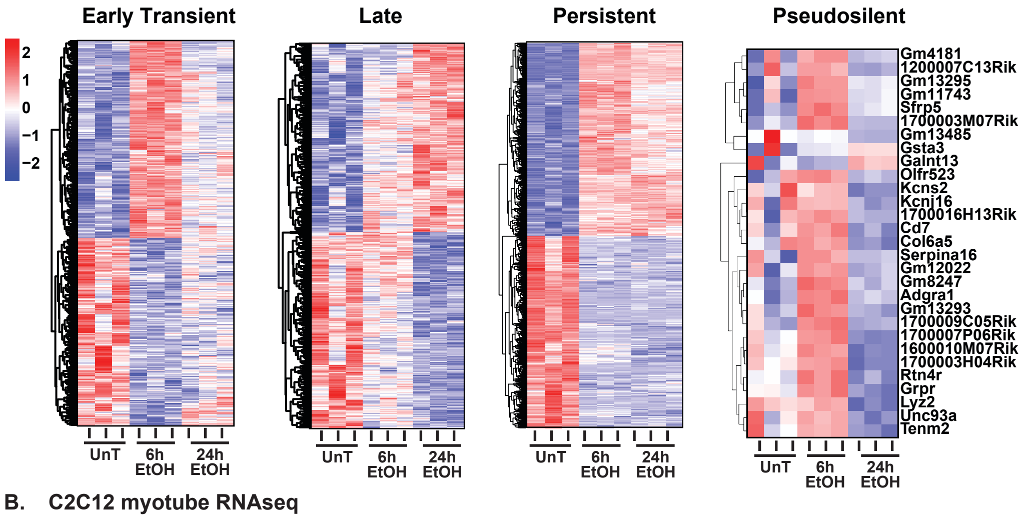

B. C2C12 myotube RNAseq

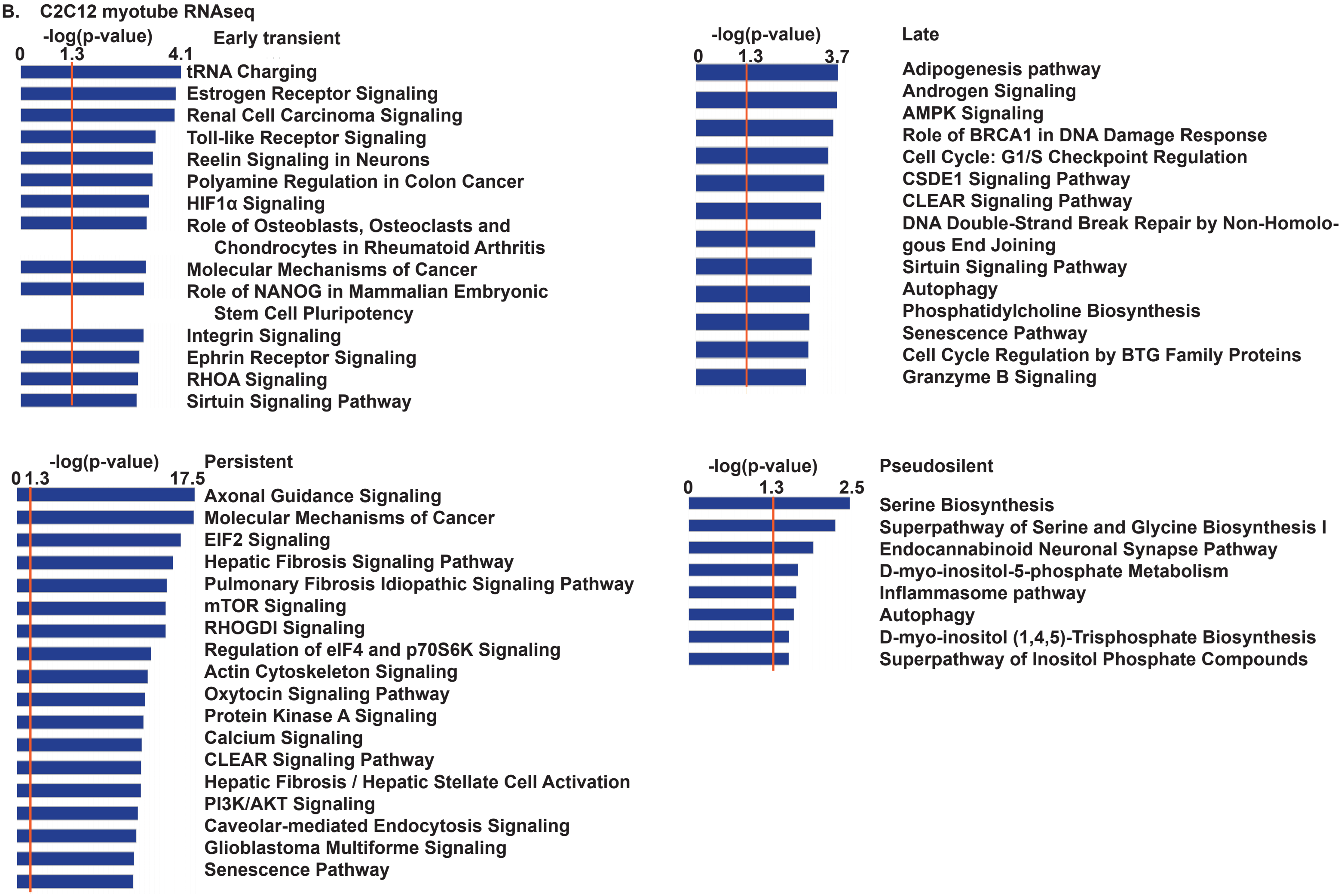

C.

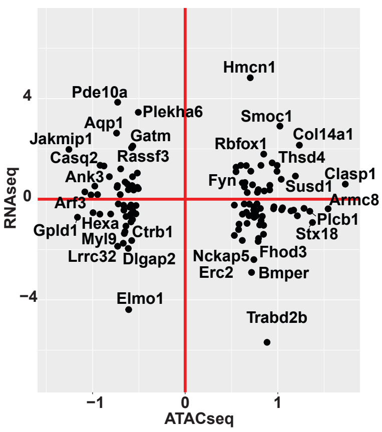

D. C2C12 myotube RNAseq

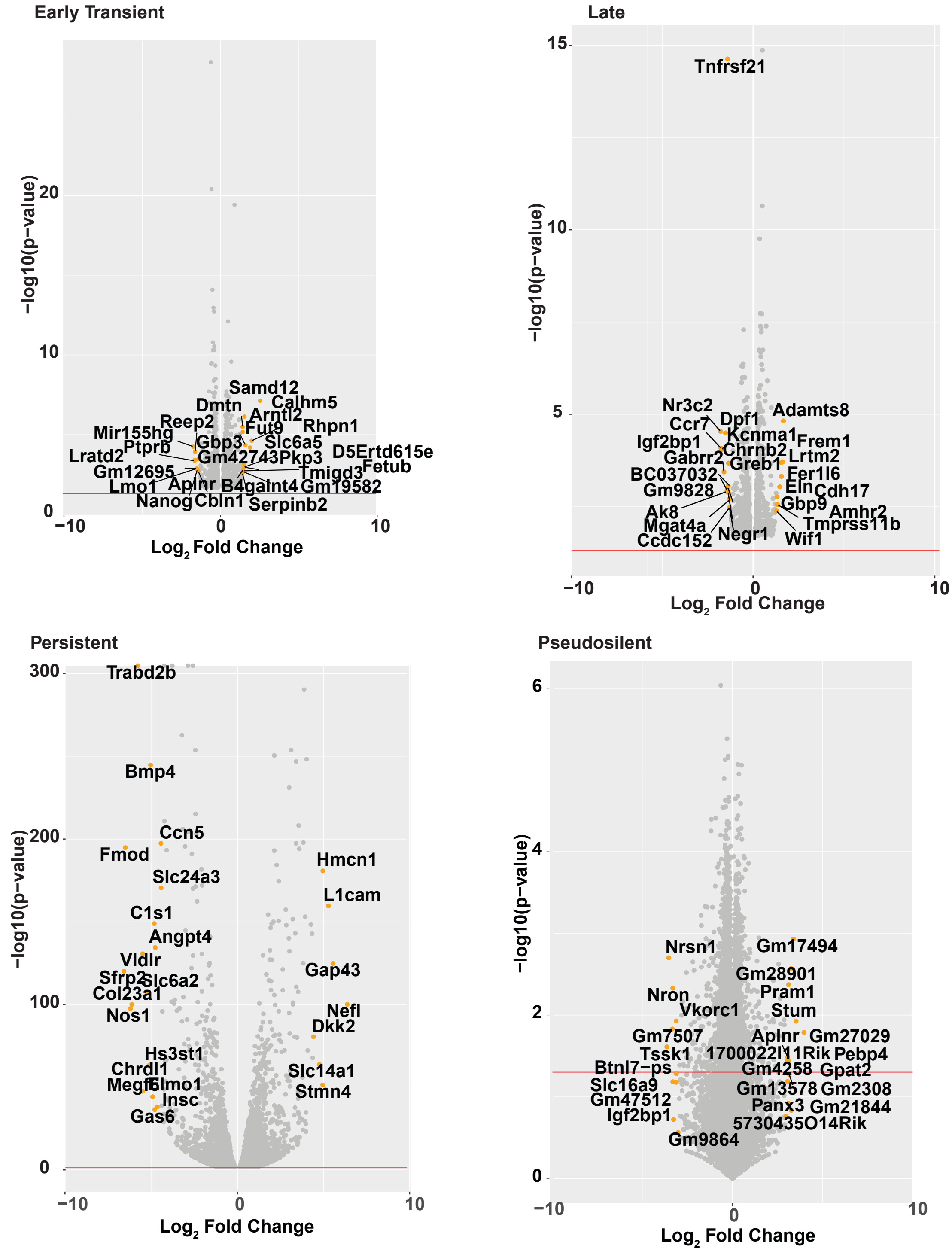

E. C2C12 myotube RNAseq Early Transient

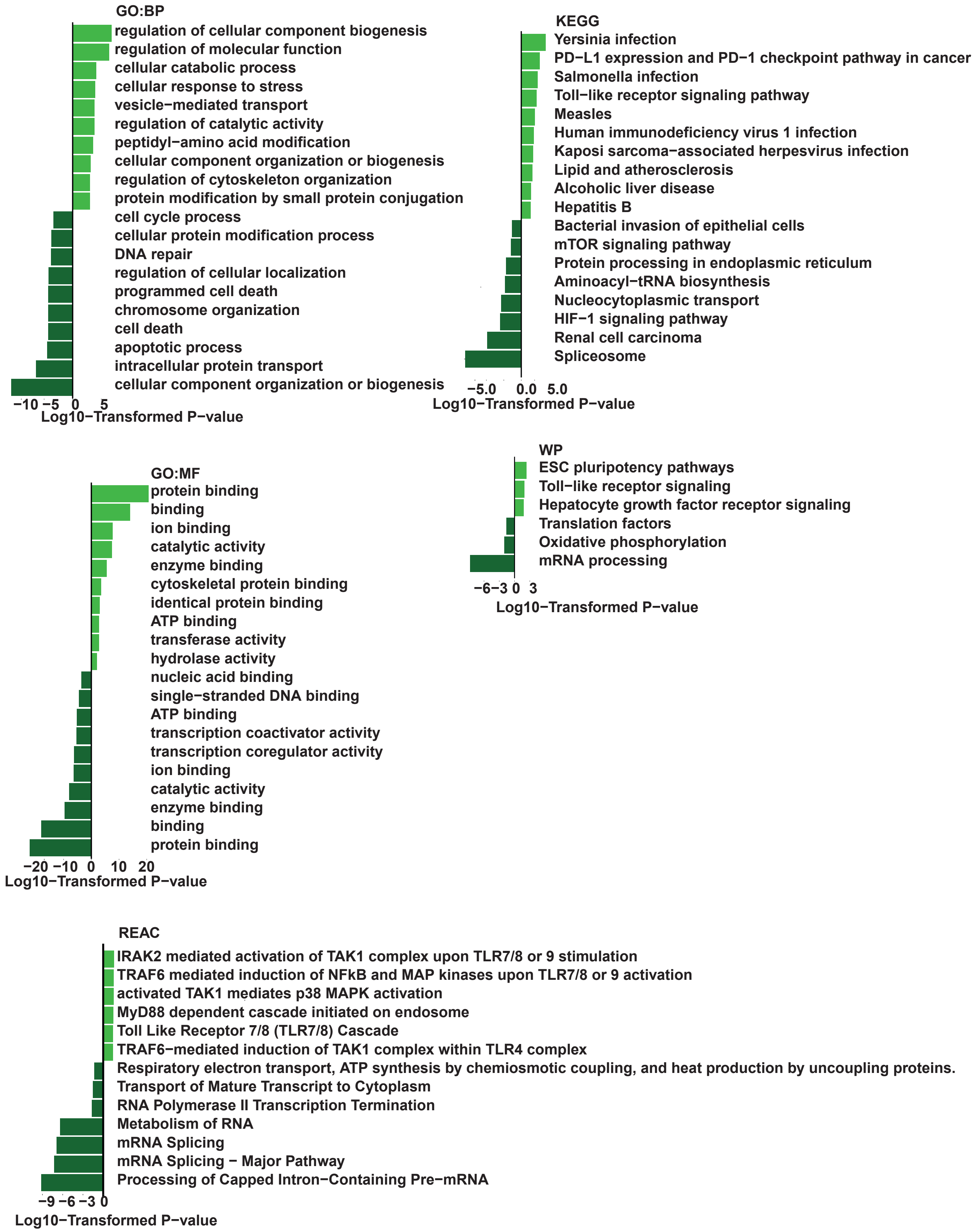

F. C2C12 myotube RNAseq Late

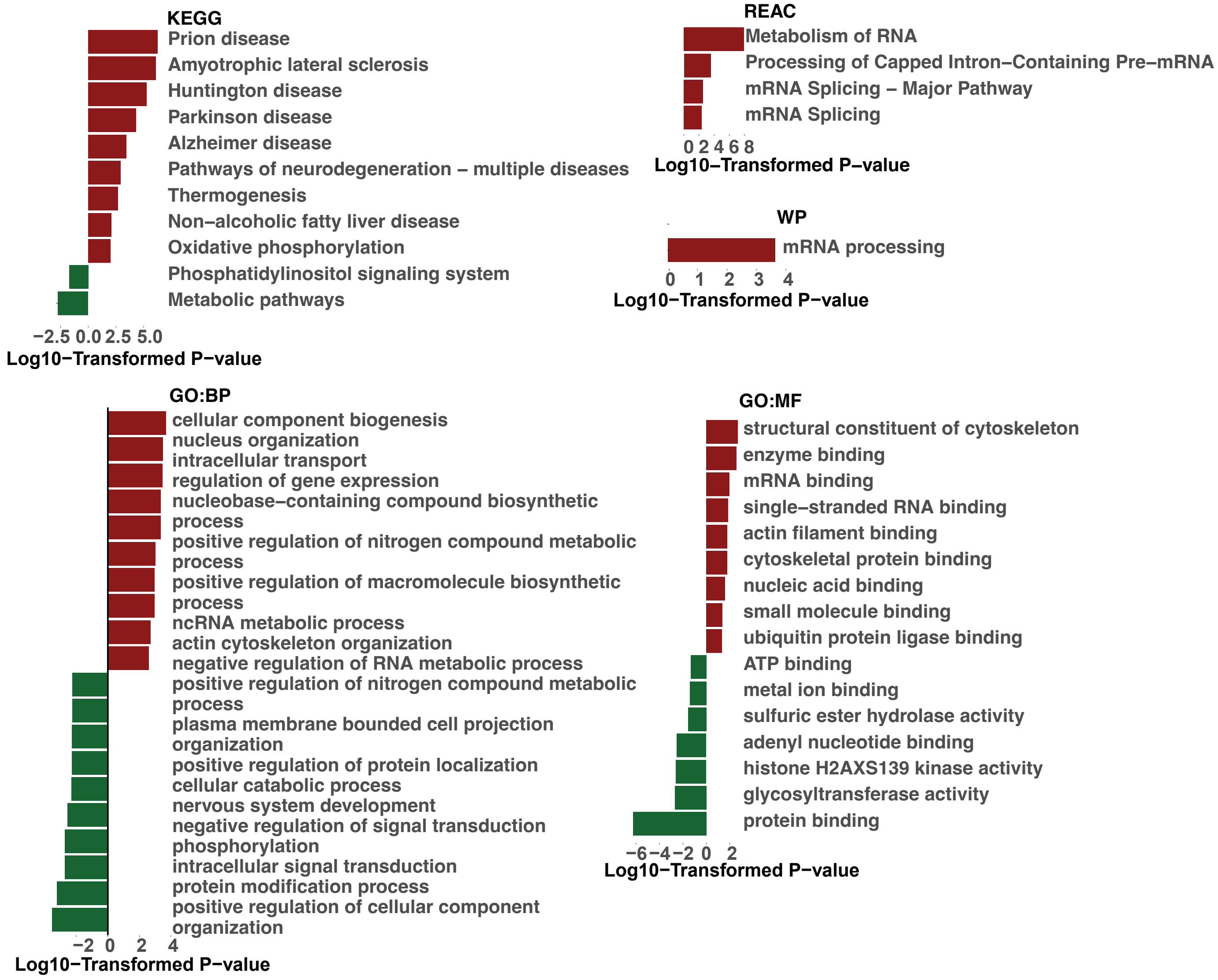

G. C2C12 myotube RNAseq Persistent

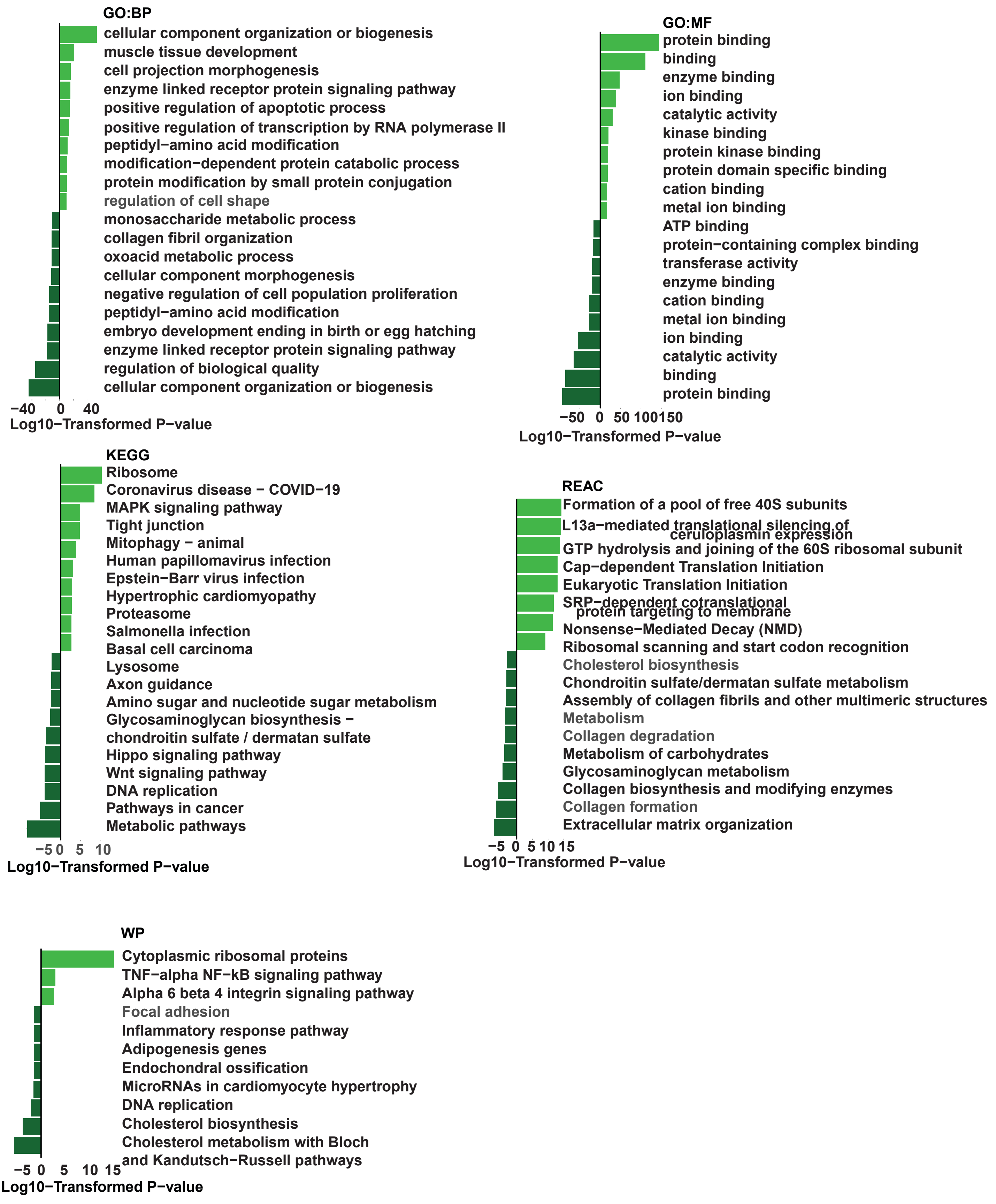

H. C2C12 myotube RNAseq Pseudosilent

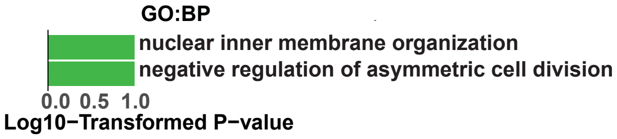

S.Fig 3

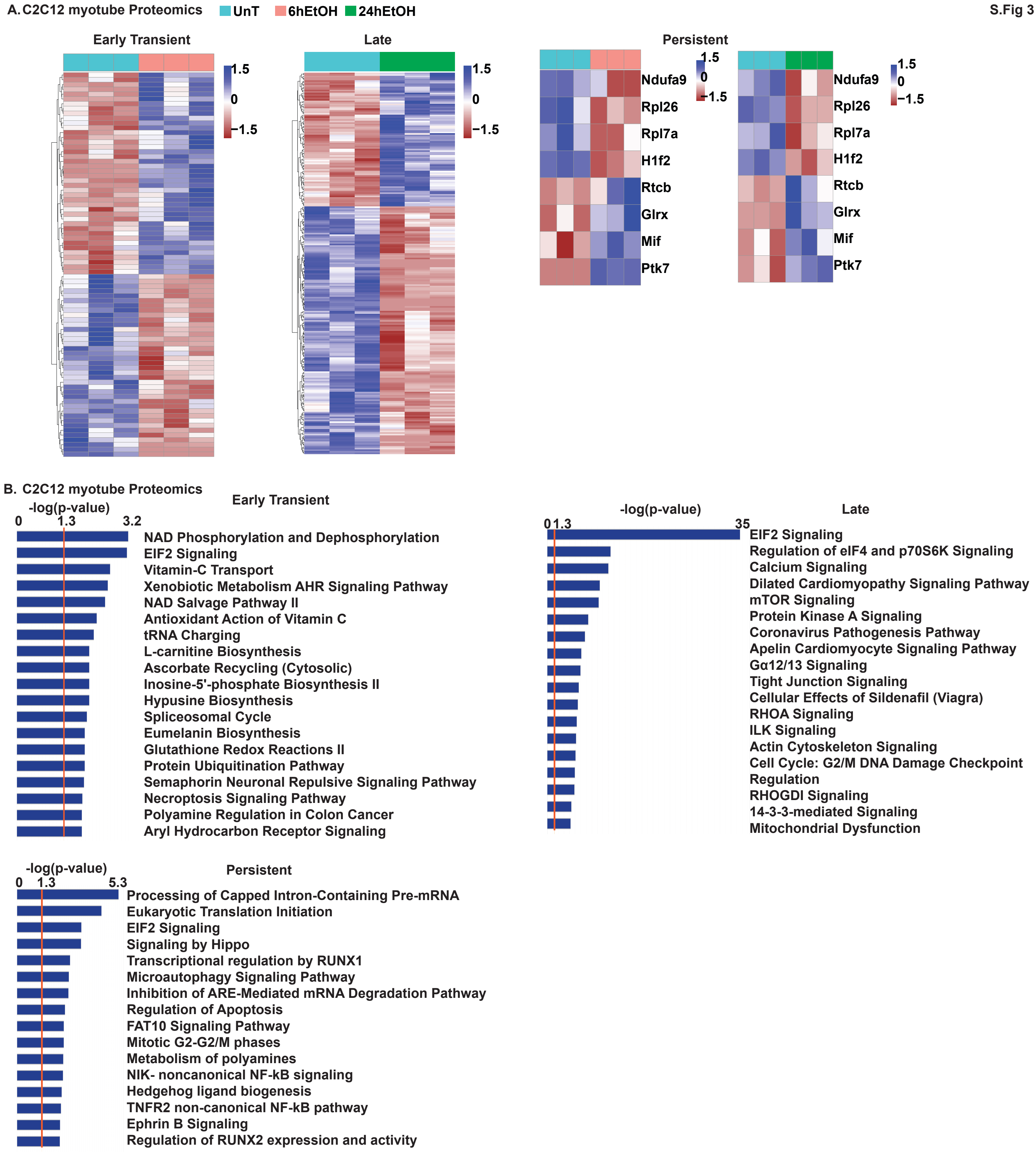

A. C2C12 myotube Proteomics
UnT
6hEtOH
24hEtOH
Early Transient
1.5
0
−1.5
Late
1.5
0
−1.5
Persistent
Ndufa9
Rpl26
Rpl7a
H1f2
Rtcb
Glrx
Mif
Ptk7
1.5
0
−1.5
B. C2C12 myotube Proteomics
Early Transient
-log(p-value)
0
1.3
3.2
NAD Phosphorylation and Dephosphorylation
EIF2 Signaling
Vitamin-C Transport
Xenobiotic Metabolism AHR Signaling Pathway
NAD Salvage Pathway II
Antioxidant Action of Vitamin C
tRNA Charging
L-carnitine Biosynthesis
Ascorbate Recycling (Cytosolic)
Inosine-5'-phosphate Biosynthesis II
Hypusine Biosynthesis
Spliceosomal Cycle
Eumelanin Biosynthesis
Glutathione Redox Reactions II
Protein Ubiquitination Pathway
Semaphorin Neuronal Repulsive Signaling Pathway
Necroptosis Signaling Pathway
Polyamine Regulation in Colon Cancer
Aryl Hydrocarbon Receptor Signaling
Late
-log(p-value)
0
1.3
35
EIF2 Signaling
Regulation of eIF4 and p70S6K Signaling
Calcium Signaling
Dilated Cardiomyopathy Signaling Pathway
mTOR Signaling
Protein Kinase A Signaling
Coronavirus Pathogenesis Pathway
Apelin Cardiomyocyte Signaling Pathway
Gα12/13 Signaling
Tight Junction Signaling
Cellular Effects of Sildenafil (Viagra)
RHOA Signaling
ILK Signaling
Actin Cytoskeleton Signaling
Cell Cycle: G2/M DNA Damage Checkpoint Regulation
RHOGDI Signaling
14-3-3-mediated Signaling
Mitochondrial Dysfunction
Persistent
-log(p-value)
0
1.3
5.3
Processing of Capped Intron-Containing Pre-mRNA
Eukaryotic Translation Initiation
EIF2 Signaling
Signaling by Hippo
Transcriptional regulation by RUNX1
Microautophagy Signaling Pathway
Inhibition of ARE-Mediated mRNA Degradation Pathway
Regulation of Apoptosis
FAT10 Signaling Pathway
Mitotic G2-G2/M phases
Metabolism of polyamines
NIK- noncanonical NF-kB signaling
Hedgehog ligand biogenesis
TNFR2 non-canonical NF-kB pathway
Ephrin B Signaling
Regulation of RUNX2 expression and activity

C. C2C12 myotube Proteomics

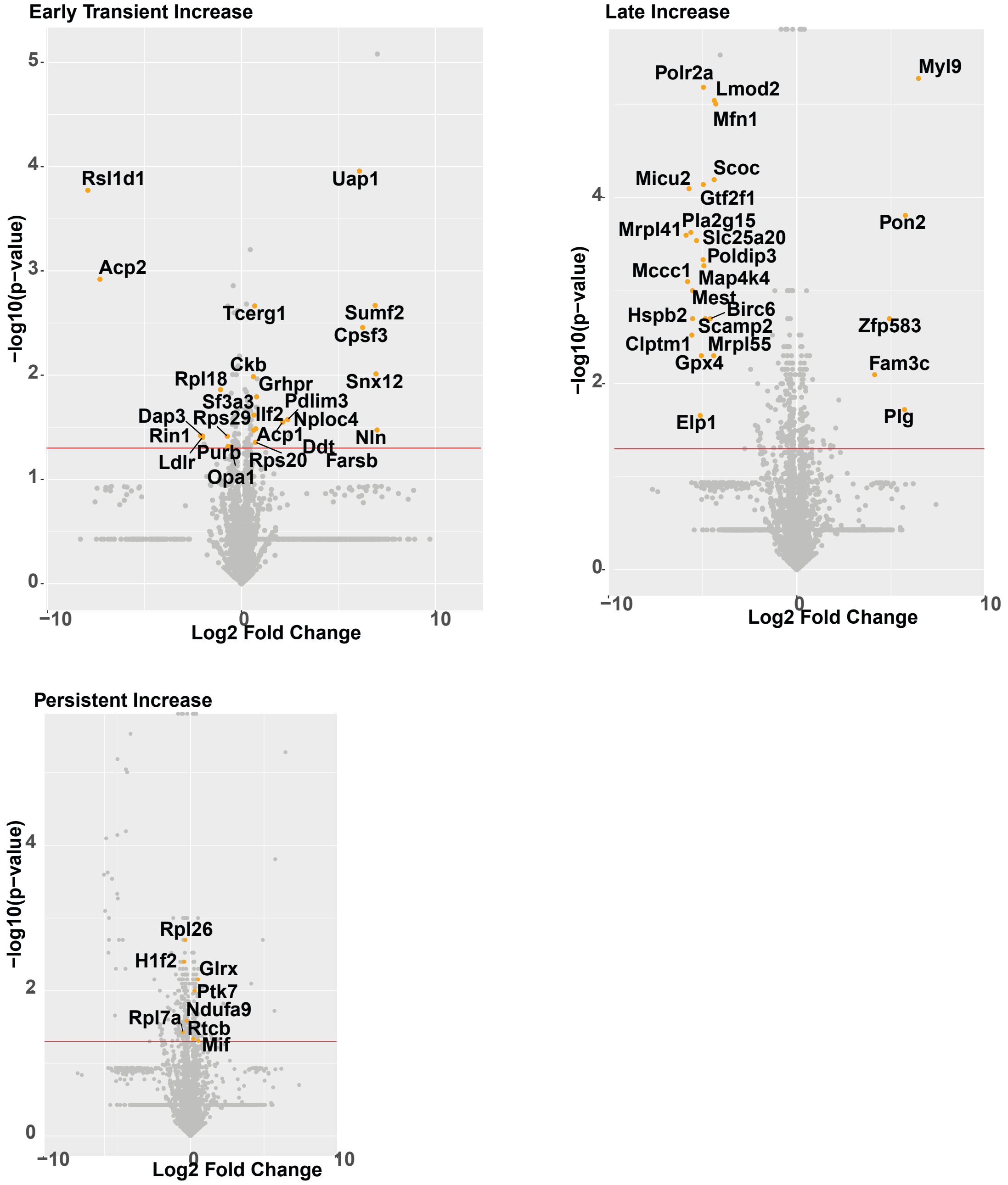

S.Fig 3

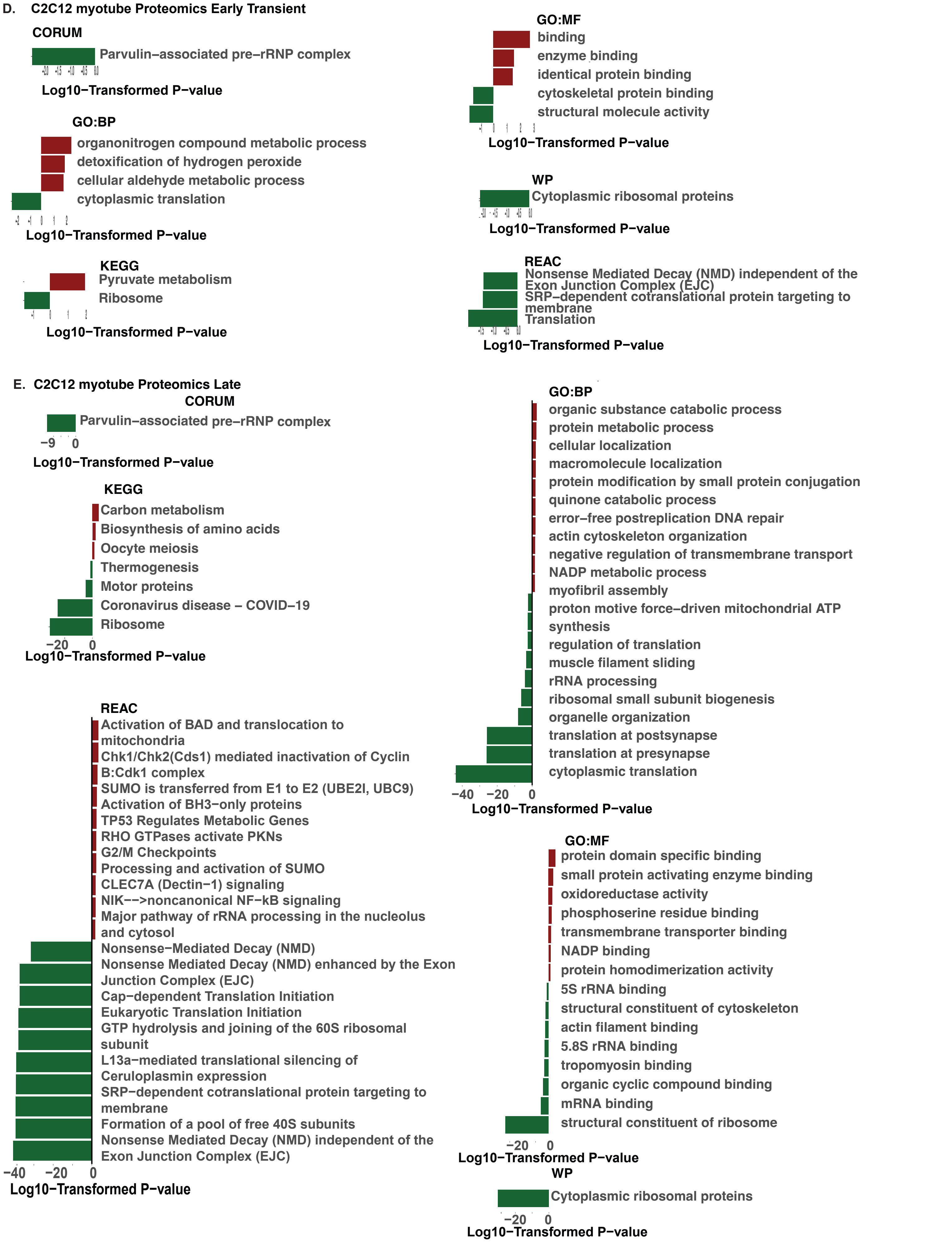

D. C2C12 myotube Proteomics Early Transient
CORUM
Parvulin−associated pre−rRNP complex
Log10−Transformed P−value
GO:BP
organonitrogen compound metabolic process
detoxification of hydrogen peroxide
cellular aldehyde metabolic process
cytoplasmic translation
Log10−Transformed P−value
KEGG
Pyruvate metabolism
Ribosome
Log10−Transformed P−value
GO:MF
binding
enzyme binding
identical protein binding
cytoskeletal protein binding
structural molecule activity
Log10−Transformed P−value
WP
Cytoplasmic ribosomal proteins
Log10−Transformed P−value
REAC
Nonsense Mediated Decay (NMD) independent of the Exon Junction Complex (EJC)
SRP−dependent cotranslational protein targeting to membrane
Translation
Log10−Transformed P−value
E. C2C12 myotube Proteomics Late
CORUM
Parvulin−associated pre−rRNP complex
−9
0
Log10−Transformed P−value
KEGG
Carbon metabolism
Biosynthesis of amino acids
Oocyte meiosis
Thermogenesis
Motor proteins
Coronavirus disease − COVID−19
Ribosome
−20
0
Log10−Transformed P−value
REAC
Activation of BAD and translocation to mitochondria
Chk1/Chk2(Cds1) mediated inactivation of Cyclin B:Cdk1 complex
SUMO is transferred from E1 to E2 (UBE2I, UBC9)
Activation of BH3−only proteins
TP53 Regulates Metabolic Genes
RHO GTPases activate PKNs
G2/M Checkpoints
Processing and activation of SUMO
CLEC7A (Dectin−1) signaling
NIK−−>noncanonical NF−kB signaling
Major pathway of rRNA processing in the nucleolus and cytosol
Nonsense−Mediated Decay (NMD)
Nonsense Mediated Decay (NMD) enhanced by the Exon Junction Complex (EJC)
Cap−dependent Translation Initiation
Eukaryotic Translation Initiation
GTP hydrolysis and joining of the 60S ribosomal subunit
L13a−mediated translational silencing of Ceruloplasmin expression
SRP−dependent cotranslational protein targeting to membrane
Formation of a pool of free 40S subunits
Nonsense Mediated Decay (NMD) independent of the Exon Junction Complex (EJC)
−40
−20
0
Log10−Transformed P−value
GO:BP
organic substance catabolic process
protein metabolic process
cellular localization
macromolecule localization
protein modification by small protein conjugation
quinone catabolic process
error−free postreplication DNA repair
actin cytoskeleton organization
negative regulation of transmembrane transport
NADP metabolic process
myofibril assembly
proton motive force−driven mitochondrial ATP synthesis
regulation of translation
muscle filament sliding
rRNA processing
ribosomal small subunit biogenesis
organelle organization
translation at postsynapse
translation at presynapse
cytoplasmic translation
−40
−20
0
Log10−Transformed P−value
GO:MF
protein domain specific binding
small protein activating enzyme binding
oxidoreductase activity
phosphoserine residue binding
transmembrane transporter binding
NADP binding
protein homodimerization activity
5S rRNA binding
structural constituent of cytoskeleton
actin filament binding
5.8S rRNA binding
tropomyosin binding
organic cyclic compound binding
mRNA binding
structural constituent of ribosome
−20
0
Log10−Transformed P−value
WP
Cytoplasmic ribosomal proteins
−20
0
Log10−Transformed P−value

F. C2C12 myotube Proteomics Persistent

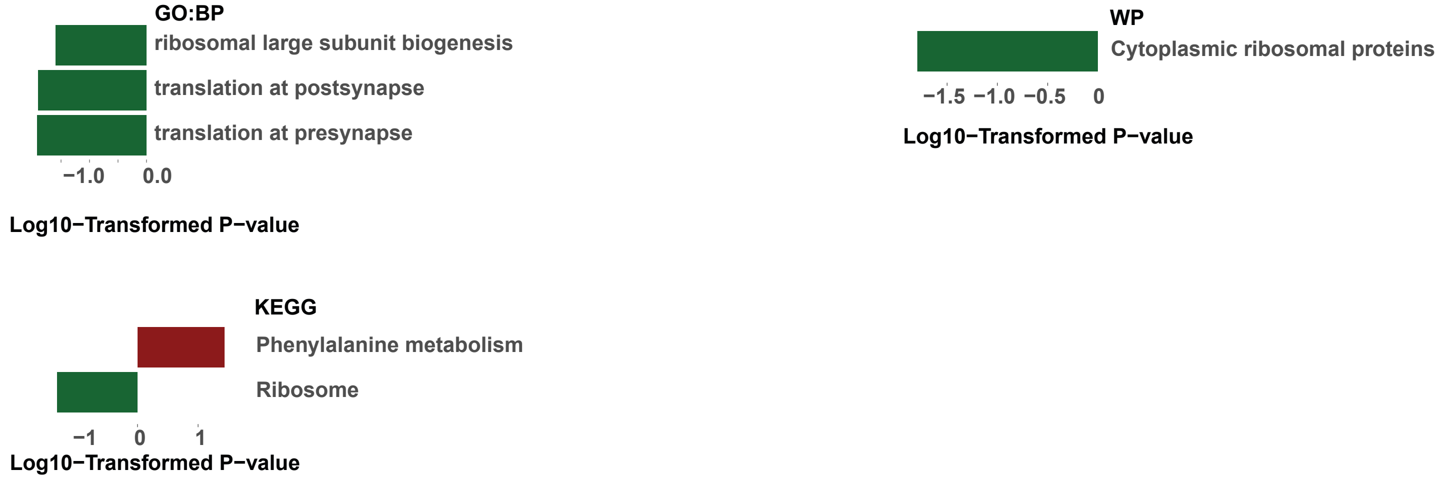

S.Fig 4

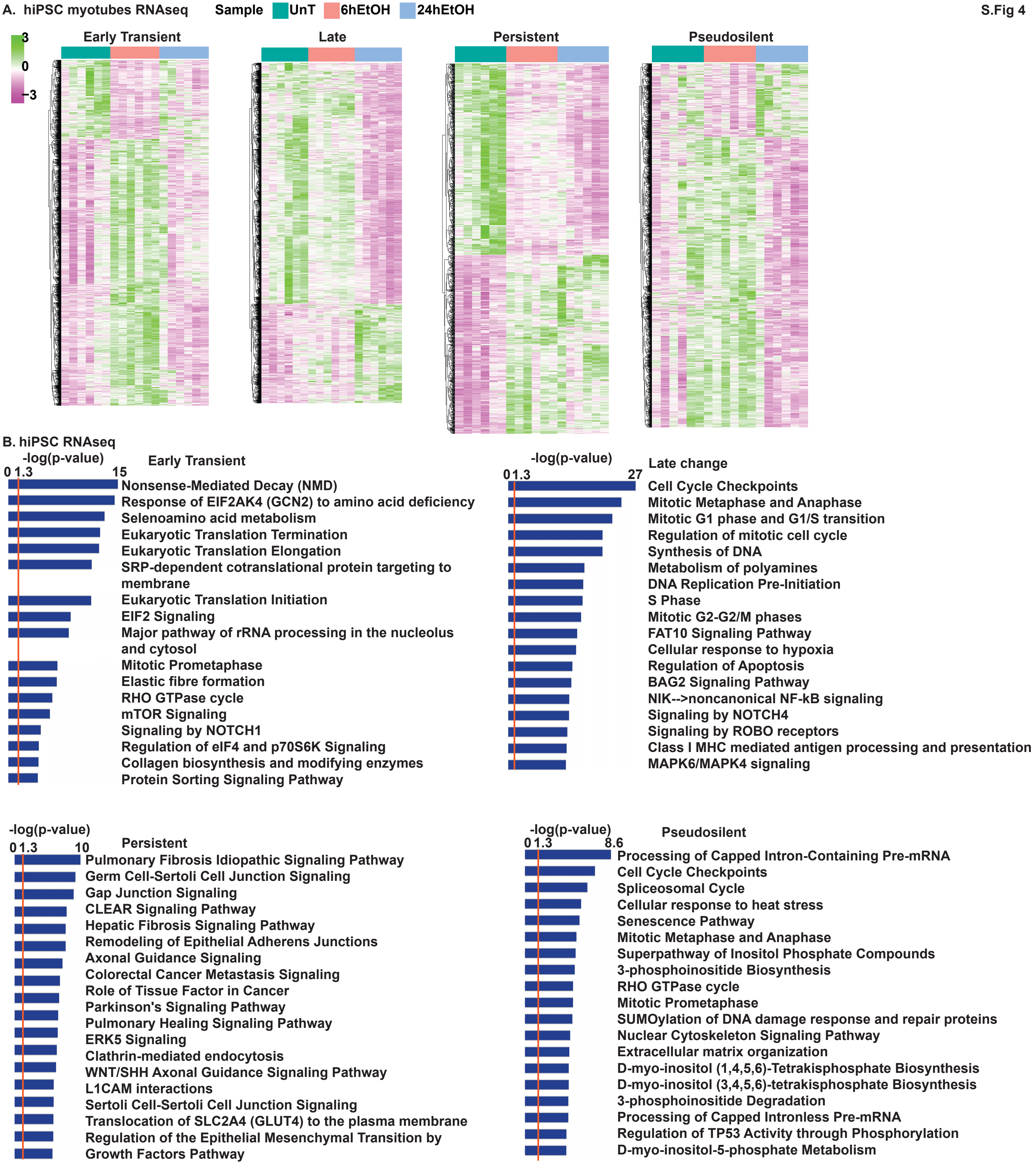
A. hiPSC myotubes RNAseq
Sample
UnT
6hEtOH
24hEtOH
3
0
-3
Early Transient
Late
Persistent
Pseudosilent
B. hiPSC RNAseq
-log(p-value)
0
1.3
15
Early Transient
Nonsense-Mediated Decay (NMD)
Response of EIF2AK4 (GCN2) to amino acid deficiency
Selenoamino acid metabolism
Eukaryotic Translation Termination
Eukaryotic Translation Elongation
SRP-dependent cotranslational protein targeting to membrane
Eukaryotic Translation Initiation
EIF2 Signaling
Major pathway of rRNA processing in the nucleolus and cytosol
Mitotic Prometaphase
Elastic fibre formation
RHO GTPase cycle
mTOR Signaling
Signaling by NOTCH1
Regulation of eIF4 and p70S6K Signaling
Collagen biosynthesis and modifying enzymes
Protein Sorting Signaling Pathway
-log(p-value)
0
1.3
27
Late change
Cell Cycle Checkpoints
Mitotic Metaphase and Anaphase
Mitotic G1 phase and G1/S transition
Regulation of mitotic cell cycle
Synthesis of DNA
Metabolism of polyamines
DNA Replication Pre-Initiation
S Phase
Mitotic G2-G2/M phases
FAT10 Signaling Pathway
Cellular response to hypoxia
Regulation of Apoptosis
BAG2 Signaling Pathway
NIK-->noncanonical NF-kB signaling
Signaling by NOTCH4
Signaling by ROBO receptors
Class I MHC mediated antigen processing and presentation
MAPK6/MAPK4 signaling
-log(p-value)
0
1.3
10
Persistent
Pulmonary Fibrosis Idiopathic Signaling Pathway
Germ Cell-Sertoli Cell Junction Signaling
Gap Junction Signaling
CLEAR Signaling Pathway
Hepatic Fibrosis Signaling Pathway
Remodeling of Epithelial Adherens Junctions
Axonal Guidance Signaling
Colorectal Cancer Metastasis Signaling
Role of Tissue Factor in Cancer
Parkinson's Signaling Pathway
Pulmonary Healing Signaling Pathway
ERK5 Signaling
Clathrin-mediated endocytosis
WNT/SHH Axonal Guidance Signaling Pathway
L1CAM interactions
Sertoli Cell-Sertoli Cell Junction Signaling
Translocation of SLC2A4 (GLUT4) to the plasma membrane
Regulation of the Epithelial Mesenchymal Transition by Growth Factors Pathway
-log(p-value)
0
1.3
8.6
Pseudosilent
Processing of Capped Intron-Containing Pre-mRNA
Cell Cycle Checkpoints
Spliceosomal Cycle
Cellular response to heat stress
Senescence Pathway
Mitotic Metaphase and Anaphase
Superpathway of Inositol Phosphate Compounds
3-phosphoinositide Biosynthesis
RHO GTPase cycle
Mitotic Prometaphase
SUMOylation of DNA damage response and repair proteins
Nuclear Cytoskeleton Signaling Pathway
Extracellular matrix organization
D-myo-inositol (1,4,5,6)-Tetrakisphosphate Biosynthesis
D-myo-inositol (3,4,5,6)-tetrakisphosphate Biosynthesis
3-phosphoinositide Degradation
Processing of Capped Intronless Pre-mRNA
Regulation of TP53 Activity through Phosphorylation
D-myo-inositol-5-phosphate Metabolism

C. hiPSC myotube RNAseq

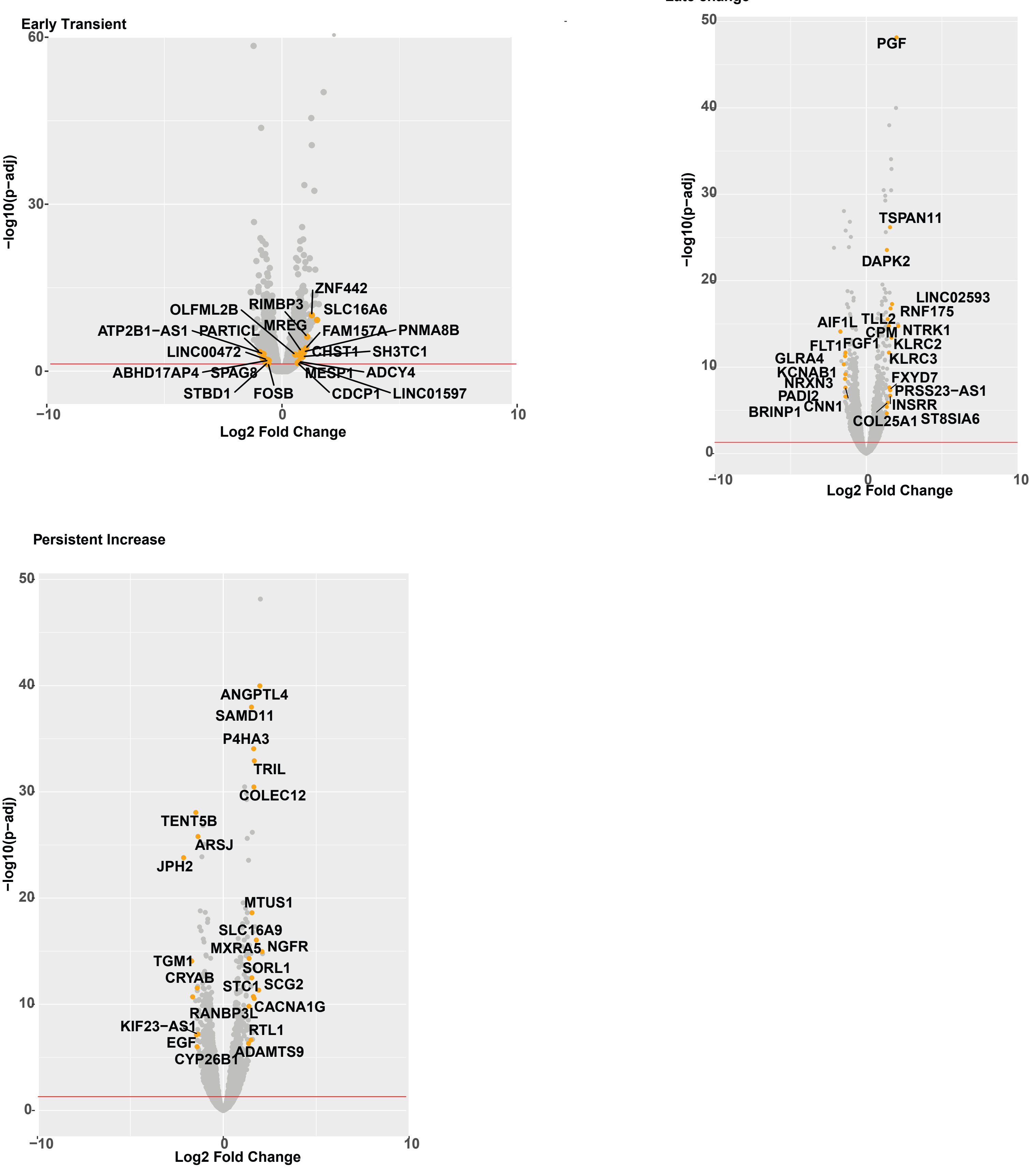

D. hiPSC RNA Early Transient

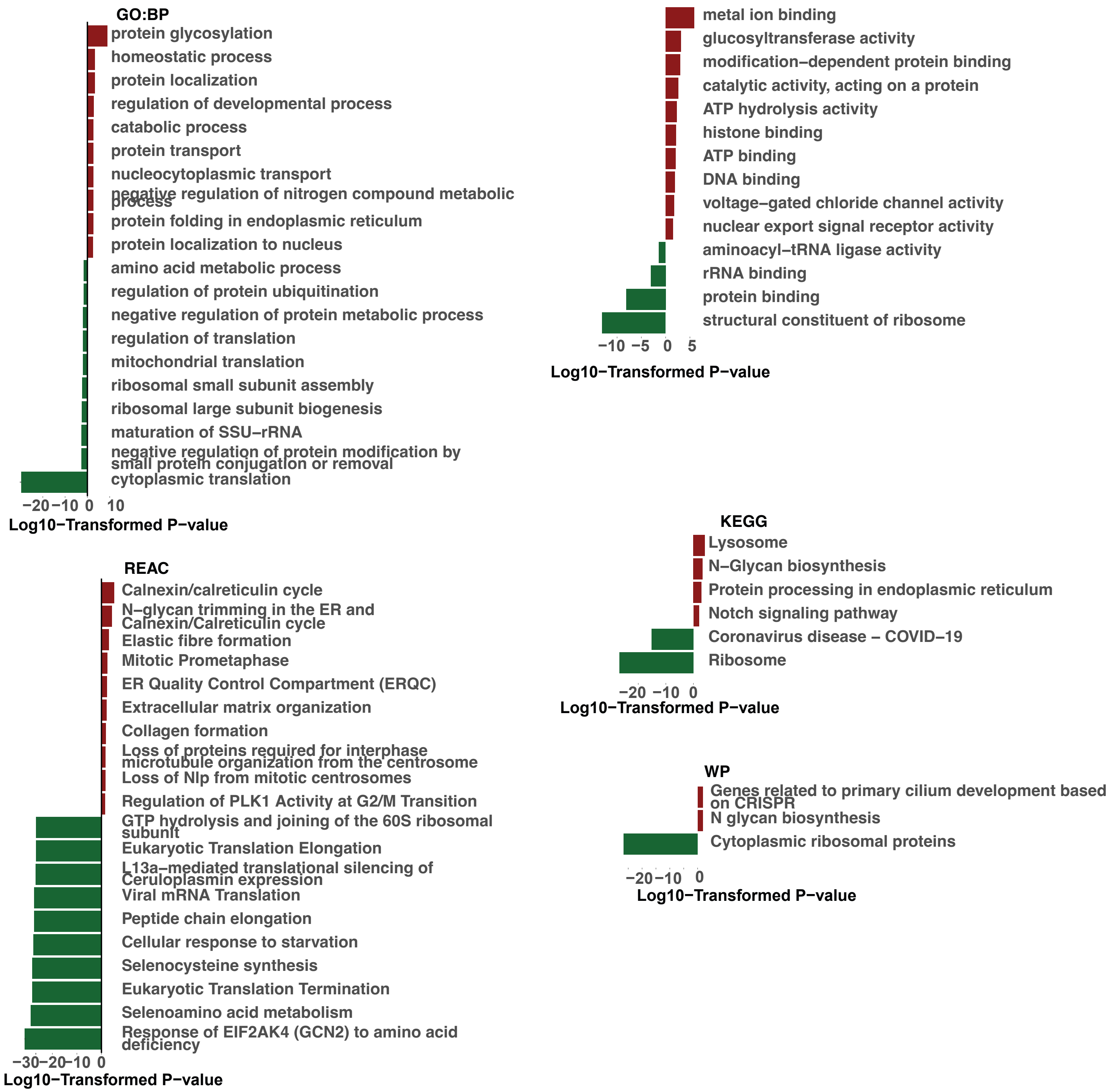

E. hiPSC myotubes RNA Late

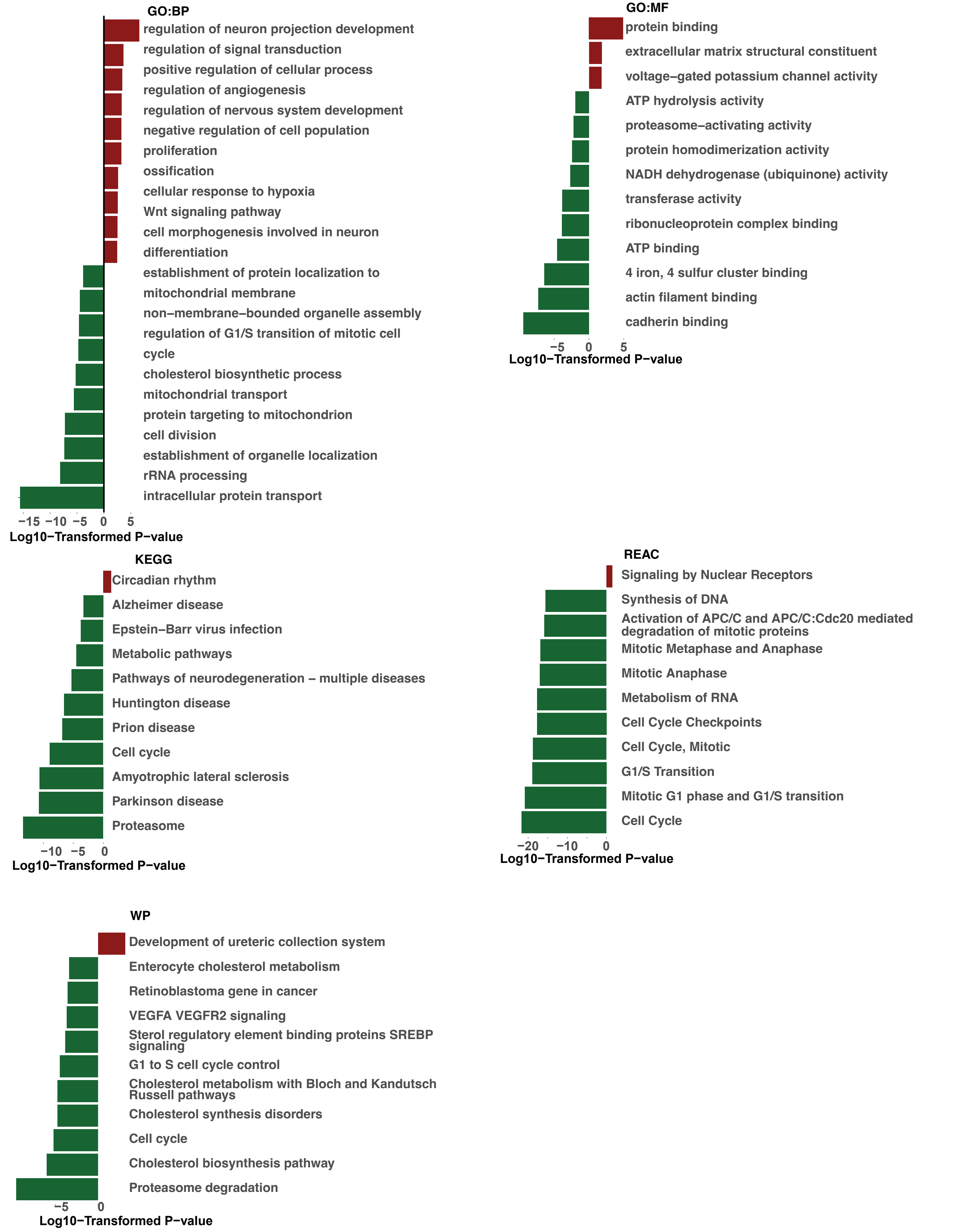

S.Fig 4

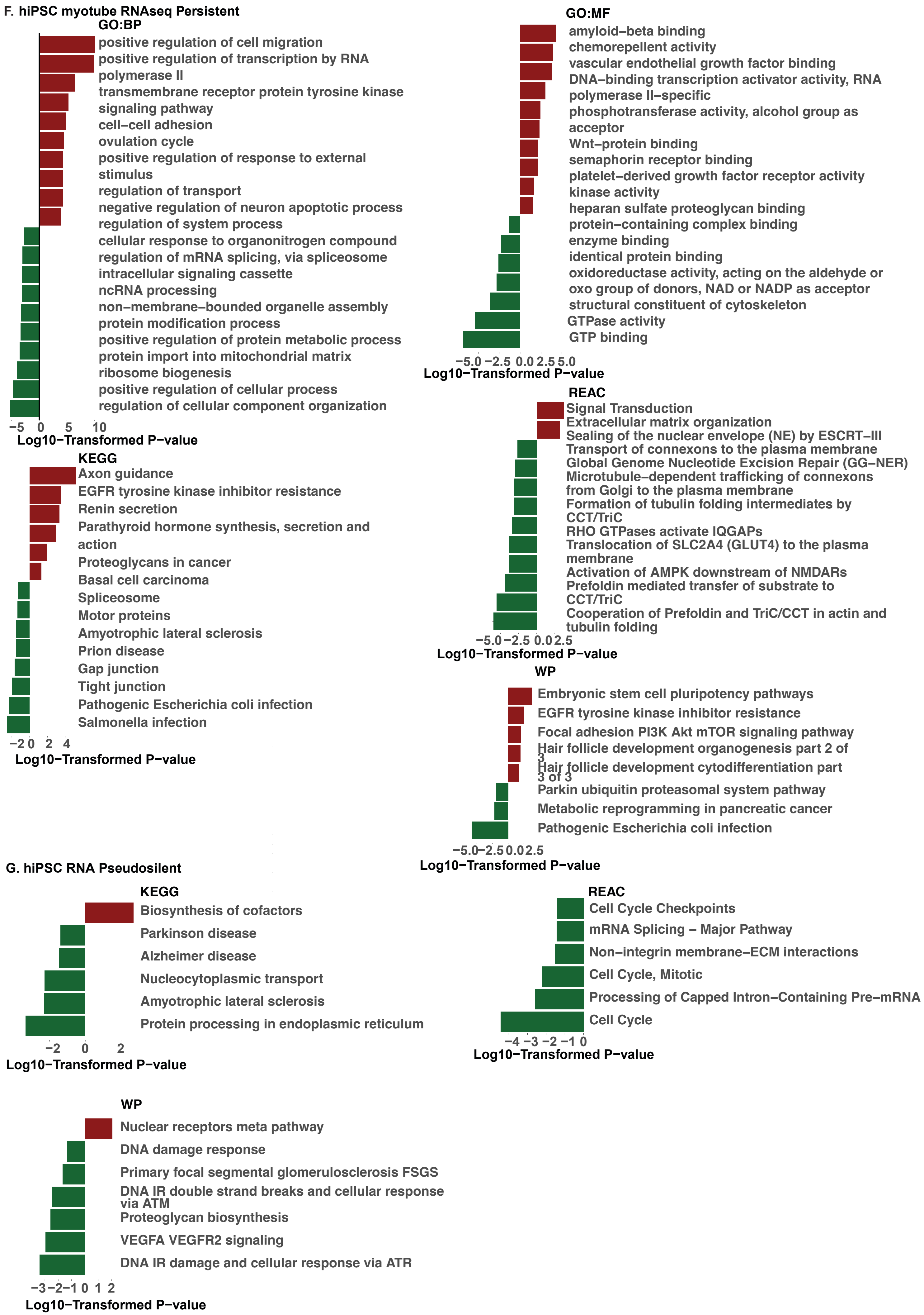
F. hiPSC myotube RNAseq Persistent
GO:BP
positive regulation of cell migration
positive regulation of transcription by RNA
polymerase II
transmembrane receptor protein tyrosine kinase
signaling pathway
cell−cell adhesion
ovulation cycle
positive regulation of response to external
stimulus
regulation of transport
negative regulation of neuron apoptotic process
regulation of system process
cellular response to organonitrogen compound
regulation of mRNA splicing, via spliceosome
intracellular signaling cassette
ncRNA processing
non−membrane−bounded organelle assembly
protein modification process
positive regulation of protein metabolic process
protein import into mitochondrial matrix
ribosome biogenesis
positive regulation of cellular process
regulation of cellular component organization
−5 0 5 10
Log10−Transformed P−value
GO:MF
amyloid−beta binding
chemorepellent activity
vascular endothelial growth factor binding
DNA−binding transcription activator activity, RNA
polymerase II−specific
phosphotransferase activity, alcohol group as
acceptor
Wnt−protein binding
semaphorin receptor binding
platelet−derived growth factor receptor activity
kinase activity
heparan sulfate proteoglycan binding
protein−containing complex binding
enzyme binding
identical protein binding
oxidoreductase activity, acting on the aldehyde or
oxo group of donors, NAD or NADP as acceptor
structural constituent of cytoskeleton
GTPase activity
GTP binding
−5.0 −2.5 0.0 2.5 5.0
Log10−Transformed P−value
KEGG
Axon guidance
EGFR tyrosine kinase inhibitor resistance
Renin secretion
Parathyroid hormone synthesis, secretion and
action
Proteoglycans in cancer
Basal cell carcinoma
Spliceosome
Motor proteins
Amyotrophic lateral sclerosis
Prion disease
Gap junction
Tight junction
Pathogenic Escherichia coli infection
Salmonella infection
−2 0 2 4
Log10−Transformed P−value
REAC
Signal Transduction
Extracellular matrix organization
Sealing of the nuclear envelope (NE) by ESCRT−III
Transport of connexons to the plasma membrane
Global Genome Nucleotide Excision Repair (GG−NER)
Microtubule−dependent trafficking of connexons
from Golgi to the plasma membrane
Formation of tubulin folding intermediates by
CCT/TriC
RHO GTPases activate IQGAPs
Translocation of SLC2A4 (GLUT4) to the plasma
membrane
Activation of AMPK downstream of NMDARs
Prefoldin mediated transfer of substrate to
CCT/TriC
Cooperation of Prefoldin and TriC/CCT in actin and
tubulin folding
−5.0 −2.5 0.0 2.5
Log10−Transformed P−value
WP
Embryonic stem cell pluripotency pathways
EGFR tyrosine kinase inhibitor resistance
Focal adhesion PI3K Akt mTOR signaling pathway
Hair follicle development organogenesis part 2 of
3
Hair follicle development cytodifferentiation part
3 of 3
Parkin ubiquitin proteasomal system pathway
Metabolic reprogramming in pancreatic cancer
Pathogenic Escherichia coli infection
−5.0 −2.5 0.0 2.5
Log10−Transformed P−value
G. hiPSC RNA Pseudosilent
KEGG
Biosynthesis of cofactors
Parkinson disease
Alzheimer disease
Nucleocytoplasmic transport
Amyotrophic lateral sclerosis
Protein processing in endoplasmic reticulum
−2 0 2
Log10−Transformed P−value
REAC
Cell Cycle Checkpoints
mRNA Splicing − Major Pathway
Non−integrin membrane−ECM interactions
Cell Cycle, Mitotic
Processing of Capped Intron−Containing Pre−mRNA
Cell Cycle
−4 −3 −2 −1 0
Log10−Transformed P−value
WP
Nuclear receptors meta pathway
DNA damage response
Primary focal segmental glomerulosclerosis FSGS
DNA IR double strand breaks and cellular response
via ATM
Proteoglycan biosynthesis
VEGFA VEGFR2 signaling
DNA IR damage and cellular response via ATR
−3 −2 −1 0 1 2
Log10−Transformed P−value

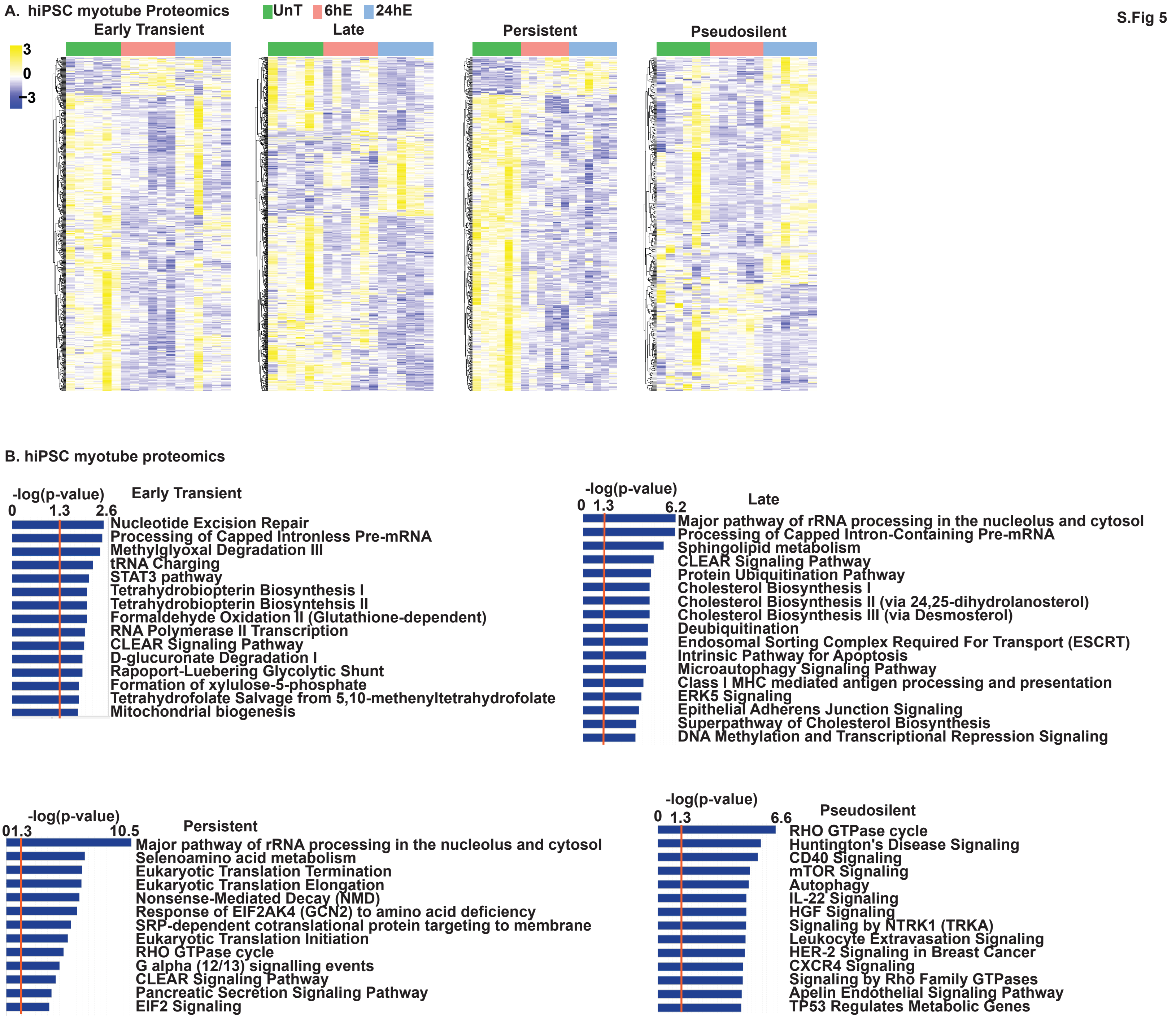
S.Fig 5
A. hiPSC myotube Proteomics
UnT
6hE
24hE
Early Transient
Late
Persistent
Pseudosilent
3
0
-3
B. hiPSC myotube proteomics
-log(p-value)
Early Transient
0
1.3
2.6
Nucleotide Excision Repair
Processing of Capped Intronless Pre-mRNA
Methylglyoxal Degradation III
tRNA Charging
STAT3 pathway
Tetrahydrobiopterin Biosynthesis I
Tetrahydrobiopterin Biosyntehsis II
Formaldehyde Oxidation II (Glutathione-dependent)
RNA Polymerase II Transcription
CLEAR Signaling Pathway
D-glucuronate Degradation I
Rapoport-Luebering Glycolytic Shunt
Formation of xylulose-5-phosphate
Tetrahydrofolate Salvage from 5,10-methenyltetrahydrofolate
Mitochondrial biogenesis
-log(p-value)
Late
0
1.3
6.2
Major pathway of rRNA processing in the nucleolus and cytosol
Processing of Capped Intron-Containing Pre-mRNA
Sphingolipid metabolism
CLEAR Signaling Pathway
Protein Ubiquitination Pathway
Cholesterol Biosynthesis I
Cholesterol Biosynthesis II (via 24,25-dihydrolanosterol)
Cholesterol Biosynthesis III (via Desmosterol)
Deubiquitination
Endosomal Sorting Complex Required For Transport (ESCRT)
Intrinsic Pathway for Apoptosis
Microautophagy Signaling Pathway
Class I MHC mediated antigen processing and presentation
ERK5 Signaling
Epithelial Adherens Junction Signaling
Superpathway of Cholesterol Biosynthesis
DNA Methylation and Transcriptional Repression Signaling
-log(p-value)
Persistent
01.3
10.5
Major pathway of rRNA processing in the nucleolus and cytosol
Selenoamino acid metabolism
Eukaryotic Translation Termination
Eukaryotic Translation Elongation
Nonsense-Mediated Decay (NMD)
Response of EIF2AK4 (GCN2) to amino acid deficiency
SRP-dependent cotranslational protein targeting to membrane
Eukaryotic Translation Initiation
RHO GTPase cycle
G alpha (12/13) signalling events
CLEAR Signaling Pathway
Pancreatic Secretion Signaling Pathway
EIF2 Signaling
-log(p-value)
Pseudosilent
0
1.3
6.6
RHO GTPase cycle
Huntington's Disease Signaling
CD40 Signaling
mTOR Signaling
Autophagy
IL-22 Signaling
HGF Signaling
Signaling by NTRK1 (TRKA)
Leukocyte Extravasation Signaling
HER-2 Signaling in Breast Cancer
CXCR4 Signaling
Signaling by Rho Family GTPases
Apelin Endothelial Signaling Pathway
TP53 Regulates Metabolic Genes

C. hiPSC myotube proteomics

S.Fig 5

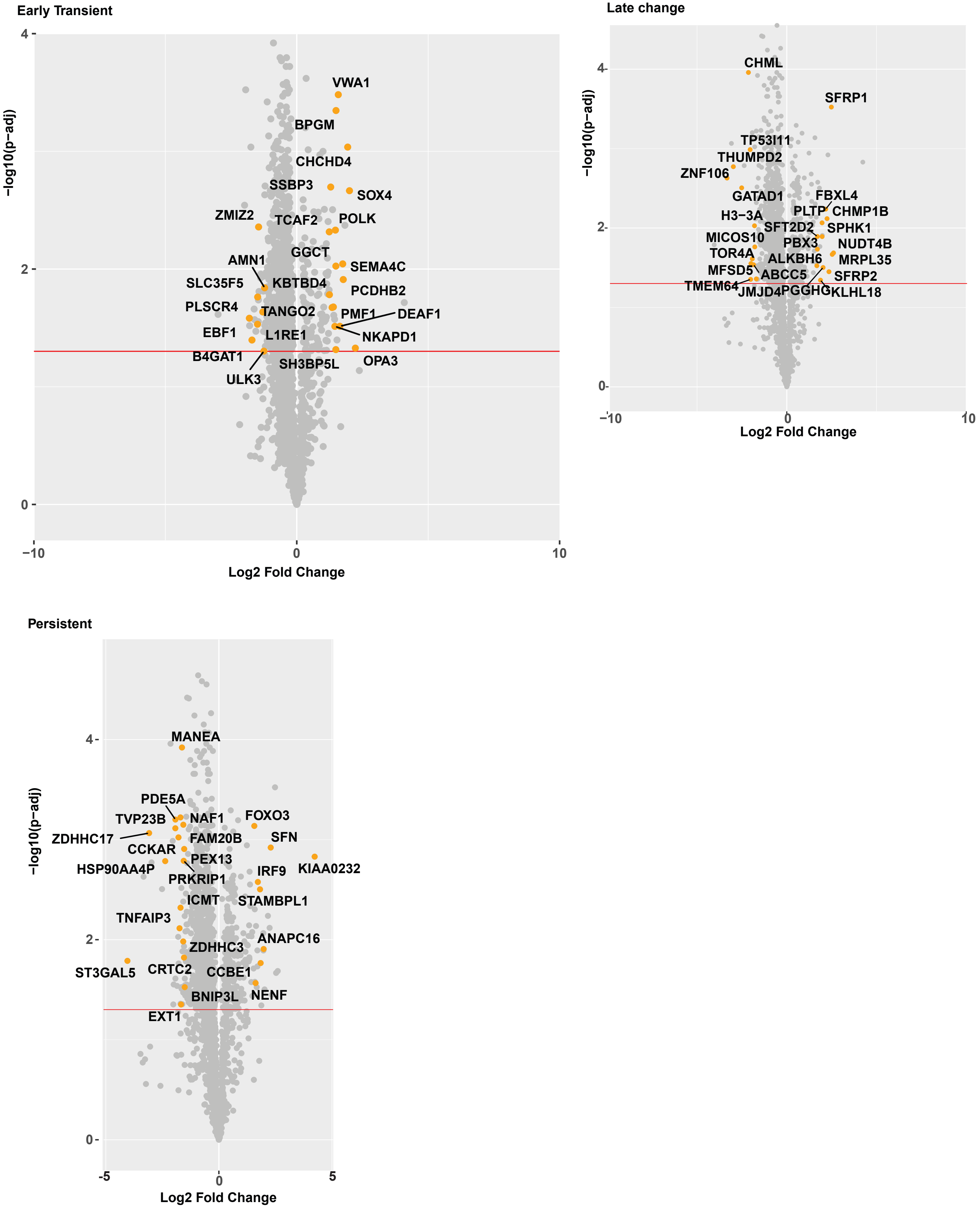

D. hiPSC myotube Proteomics Early Transient

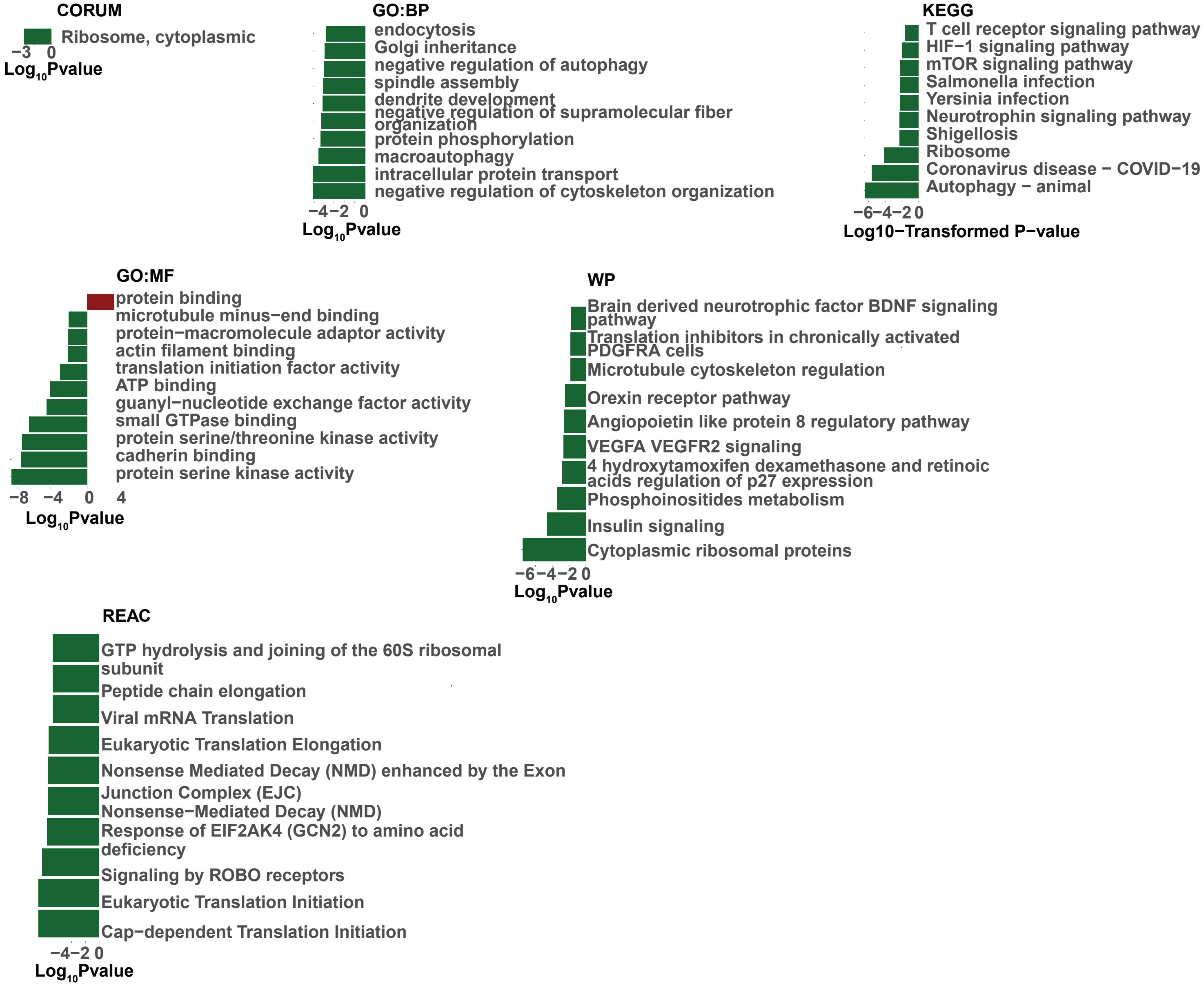

S.Fig 5

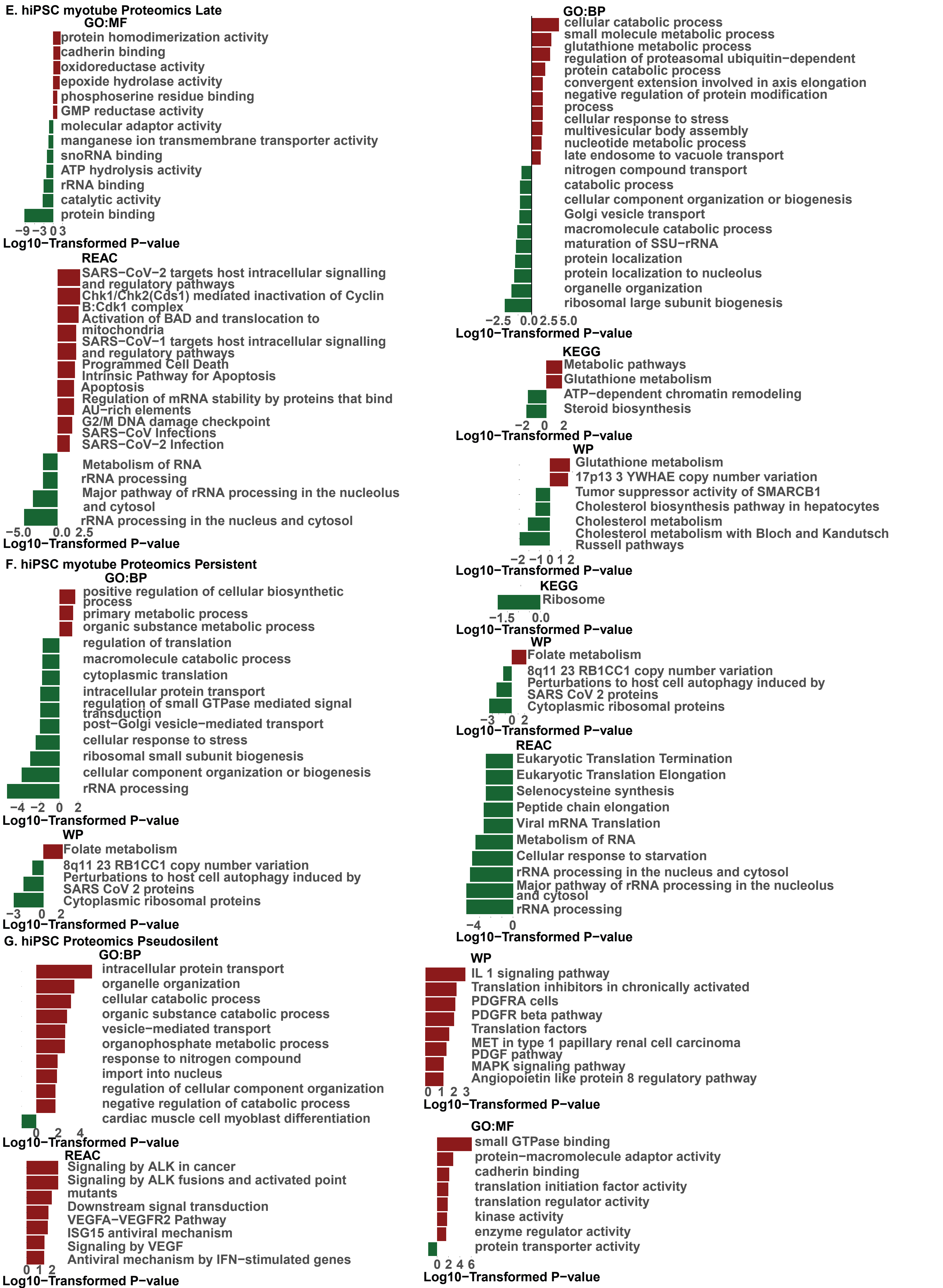
E. hiPSC myotube Proteomics Late
GO:MF
protein homodimerization activity
cadherin binding
oxidoreductase activity
epoxide hydrolase activity
phosphoserine residue binding
GMP reductase activity
molecular adaptor activity
manganese ion transmembrane transporter activity
snoRNA binding
ATP hydrolysis activity
rRNA binding
catalytic activity
protein binding
−9 −3 0 3
Log10−Transformed P−value
REAC
SARS−CoV−2 targets host intracellular signalling and regulatory pathways
Chk1/Chk2(Cds1) mediated inactivation of Cyclin B:Cdk1 complex
Activation of BAD and translocation to mitochondria
SARS−CoV−1 targets host intracellular signalling and regulatory pathways
Programmed Cell Death
Intrinsic Pathway for Apoptosis
Apoptosis
Regulation of mRNA stability by proteins that bind AU−rich elements
G2/M DNA damage checkpoint
SARS−CoV Infections
SARS−CoV−2 Infection
Metabolism of RNA
rRNA processing
Major pathway of rRNA processing in the nucleolus and cytosol
rRNA processing in the nucleus and cytosol
−5.0 0.0 2.5
Log10−Transformed P−value
GO:BP
cellular catabolic process
small molecule metabolic process
glutathione metabolic process
regulation of proteasomal ubiquitin−dependent
protein catabolic process
convergent extension involved in axis elongation
negative regulation of protein modification process
cellular response to stress
multivesicular body assembly
nucleotide metabolic process
late endosome to vacuole transport
nitrogen compound transport
catabolic process
cellular component organization or biogenesis
Golgi vesicle transport
macromolecule catabolic process
maturation of SSU−rRNA
protein localization
protein localization to nucleolus
organelle organization
ribosomal large subunit biogenesis
−2.5 0.0 2.5 5.0
Log10−Transformed P−value
KEGG
Metabolic pathways
Glutathione metabolism
ATP−dependent chromatin remodeling
Steroid biosynthesis
−2 0 2
Log10−Transformed P−value
WP
Glutathione metabolism
17p13 3 YWHAE copy number variation
Tumor suppressor activity of SMARCB1
Cholesterol biosynthesis pathway in hepatocytes
Cholesterol metabolism
Cholesterol metabolism with Bloch and Kandutsch Russell pathways
−2 −1 0 1 2
Log10−Transformed P−value
F. hiPSC myotube Proteomics Persistent
GO:BP
positive regulation of cellular biosynthetic process
primary metabolic process
organic substance metabolic process
regulation of translation
macromolecule catabolic process
cytoplasmic translation
intracellular protein transport
regulation of small GTPase mediated signal transduction
post−Golgi vesicle−mediated transport
cellular response to stress
ribosomal small subunit biogenesis
cellular component organization or biogenesis
rRNA processing
−4 −2 0 2
Log10−Transformed P−value
WP
Folate metabolism
8q11 23 RB1CC1 copy number variation
Perturbations to host cell autophagy induced by SARS CoV 2 proteins
Cytoplasmic ribosomal proteins
−3 0 2
Log10−Transformed P−value
KEGG
Ribosome
−1.5 0.0
Log10−Transformed P−value
WP
Folate metabolism
8q11 23 RB1CC1 copy number variation
Perturbations to host cell autophagy induced by SARS CoV 2 proteins
Cytoplasmic ribosomal proteins
−3 0 2
Log10−Transformed P−value
REAC
Eukaryotic Translation Termination
Eukaryotic Translation Elongation
Selenocysteine synthesis
Peptide chain elongation
Viral mRNA Translation
Metabolism of RNA
Cellular response to starvation
rRNA processing in the nucleus and cytosol
Major pathway of rRNA processing in the nucleolus and cytosol
rRNA processing
−4 0
Log10−Transformed P−value
G. hiPSC Proteomics Pseudosilent
GO:BP
intracellular protein transport
organelle organization
cellular catabolic process
organic substance catabolic process
vesicle−mediated transport
organophosphate metabolic process
response to nitrogen compound
import into nucleus
regulation of cellular component organization
negative regulation of catabolic process
cardiac muscle cell myoblast differentiation
0 2 4
Log10−Transformed P−value
REAC
Signaling by ALK in cancer
Signaling by ALK fusions and activated point mutants
Downstream signal transduction
VEGFA−VEGFR2 Pathway
ISG15 antiviral mechanism
Signaling by VEGF
Antiviral mechanism by IFN−stimulated genes
0 1 2
Log10−Transformed P−value
WP
IL 1 signaling pathway
Translation inhibitors in chronically activated PDGFRA cells
PDGFR beta pathway
Translation factors
MET in type 1 papillary renal cell carcinoma
PDGF pathway
MAPK signaling pathway
Angiopoietin like protein 8 regulatory pathway
0 1 2 3
Log10−Transformed P−value
GO:MF
small GTPase binding
protein−macromolecule adaptor activity
cadherin binding
translation initiation factor activity
translation regulator activity
kinase activity
enzyme regulator activity
protein transporter activity
0 2 4 6
Log10−Transformed P−value

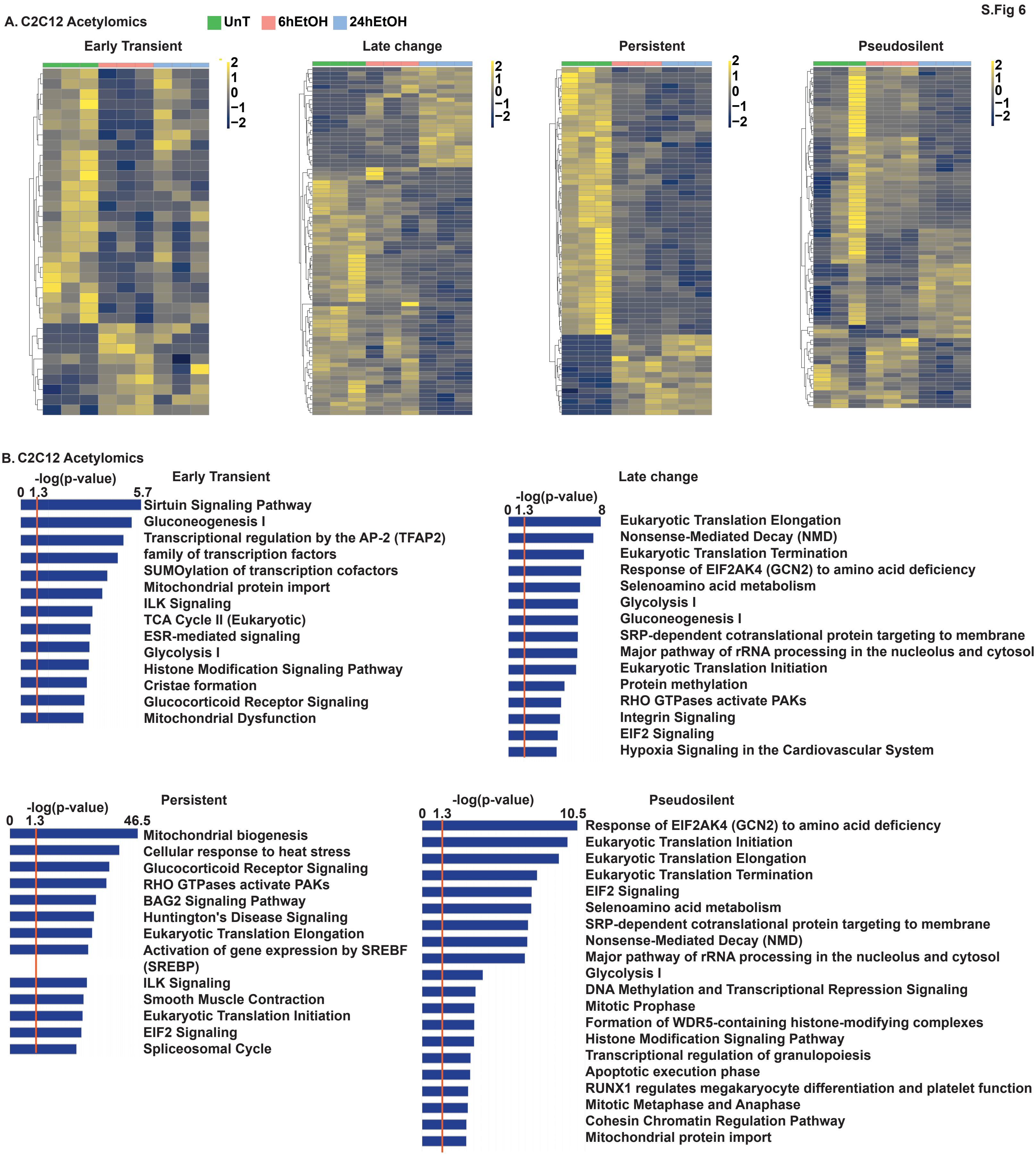
S.Fig 6
A. C2C12 Acetylomics
UnT
6hEtOH
24hEtOH
Early Transient
Late change
Persistent
Pseudosilent
2
1
0
−1
−2
B. C2C12 Acetylomics
Early Transient
-log(p-value)
0 1.3 5.7
Sirtuin Signaling Pathway
Gluconeogenesis I
Transcriptional regulation by the AP-2 (TFAP2)
family of transcription factors
SUMOylation of transcription cofactors
Mitochondrial protein import
ILK Signaling
TCA Cycle II (Eukaryotic)
ESR-mediated signaling
Glycolysis I
Histone Modification Signaling Pathway
Cristae formation
Glucocorticoid Receptor Signaling
Mitochondrial Dysfunction
Late change
-log(p-value)
0 1.3 8
Eukaryotic Translation Elongation
Nonsense-Mediated Decay (NMD)
Eukaryotic Translation Termination
Response of EIF2AK4 (GCN2) to amino acid deficiency
Selenoamino acid metabolism
Glycolysis I
Gluconeogenesis I
SRP-dependent cotranslational protein targeting to membrane
Major pathway of rRNA processing in the nucleolus and cytosol
Eukaryotic Translation Initiation
Protein methylation
RHO GTPases activate PAKs
Integrin Signaling
EIF2 Signaling
Hypoxia Signaling in the Cardiovascular System
Persistent
-log(p-value)
0 1.3 46.5
Mitochondrial biogenesis
Cellular response to heat stress
Glucocorticoid Receptor Signaling
RHO GTPases activate PAKs
BAG2 Signaling Pathway
Huntington's Disease Signaling
Eukaryotic Translation Elongation
Activation of gene expression by SREBF
(SREBP)
ILK Signaling
Smooth Muscle Contraction
Eukaryotic Translation Initiation
EIF2 Signaling
Spliceosomal Cycle
Pseudosilent
-log(p-value)
0 1.3 10.5
Response of EIF2AK4 (GCN2) to amino acid deficiency
Eukaryotic Translation Initiation
Eukaryotic Translation Elongation
Eukaryotic Translation Termination
EIF2 Signaling
Selenoamino acid metabolism
SRP-dependent cotranslational protein targeting to membrane
Nonsense-Mediated Decay (NMD)
Major pathway of rRNA processing in the nucleolus and cytosol
Glycolysis I
DNA Methylation and Transcriptional Repression Signaling
Mitotic Prophase
Formation of WDR5-containing histone-modifying complexes
Histone Modification Signaling Pathway
Transcriptional regulation of granulopoiesis
Apoptotic execution phase
RUNX1 regulates megakaryocyte differentiation and platelet function
Mitotic Metaphase and Anaphase
Cohesin Chromatin Regulation Pathway
Mitochondrial protein import

C. C2C12 Acetylome

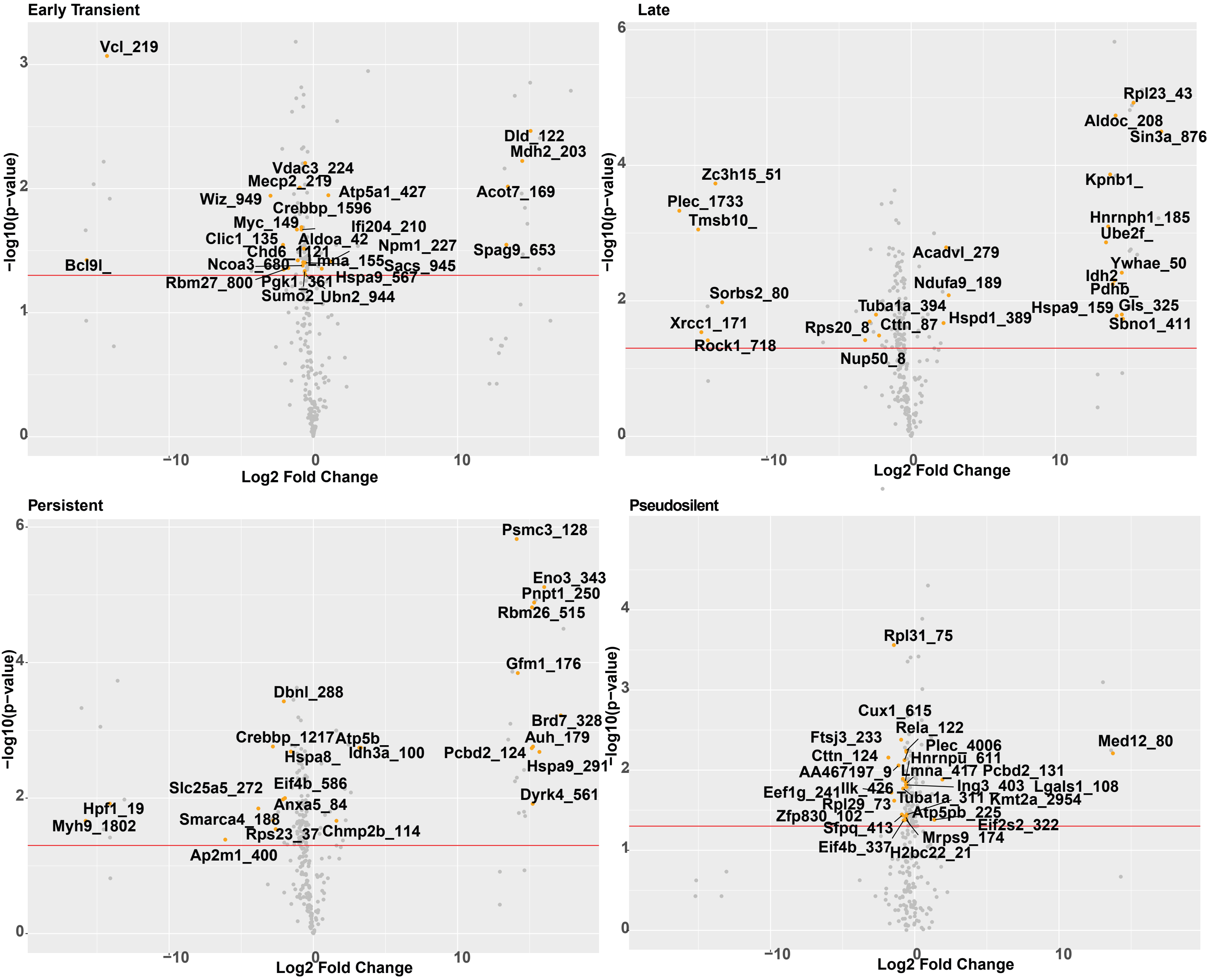

D. C2C12 myotube Acetylomics Early Transient

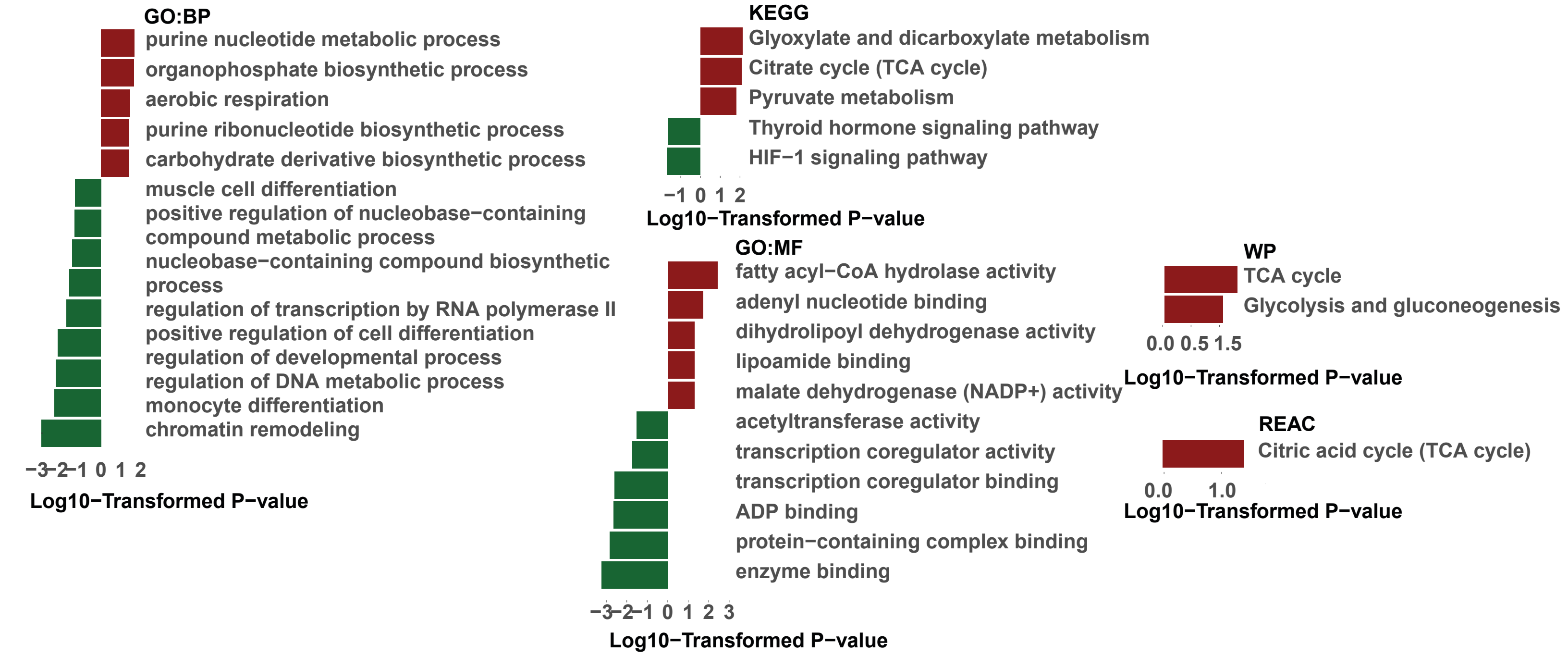

E. C2C12 myotube Acetylomics Late

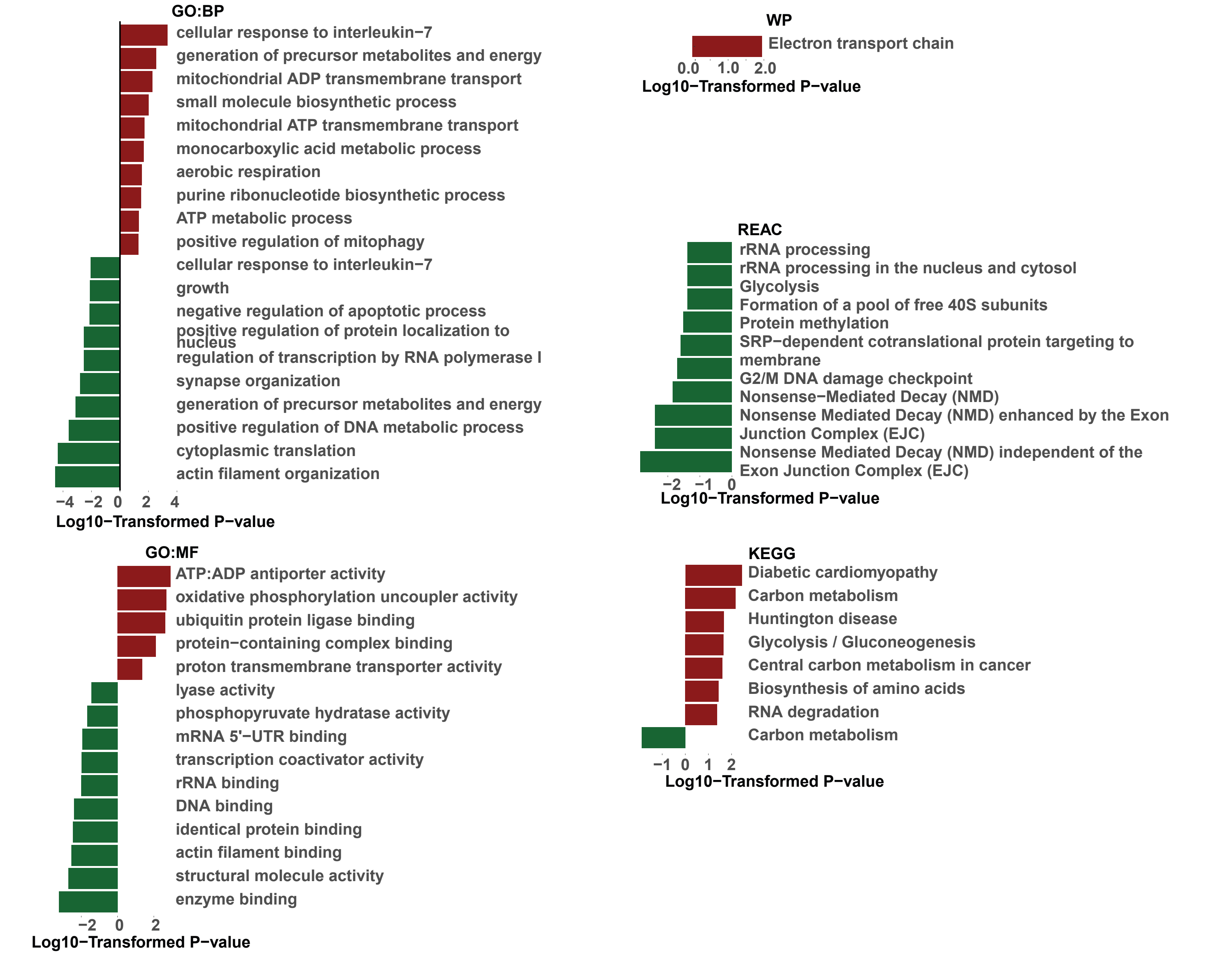

S.Fig 6

F. C2C12 myotube Acetylomics Persistent

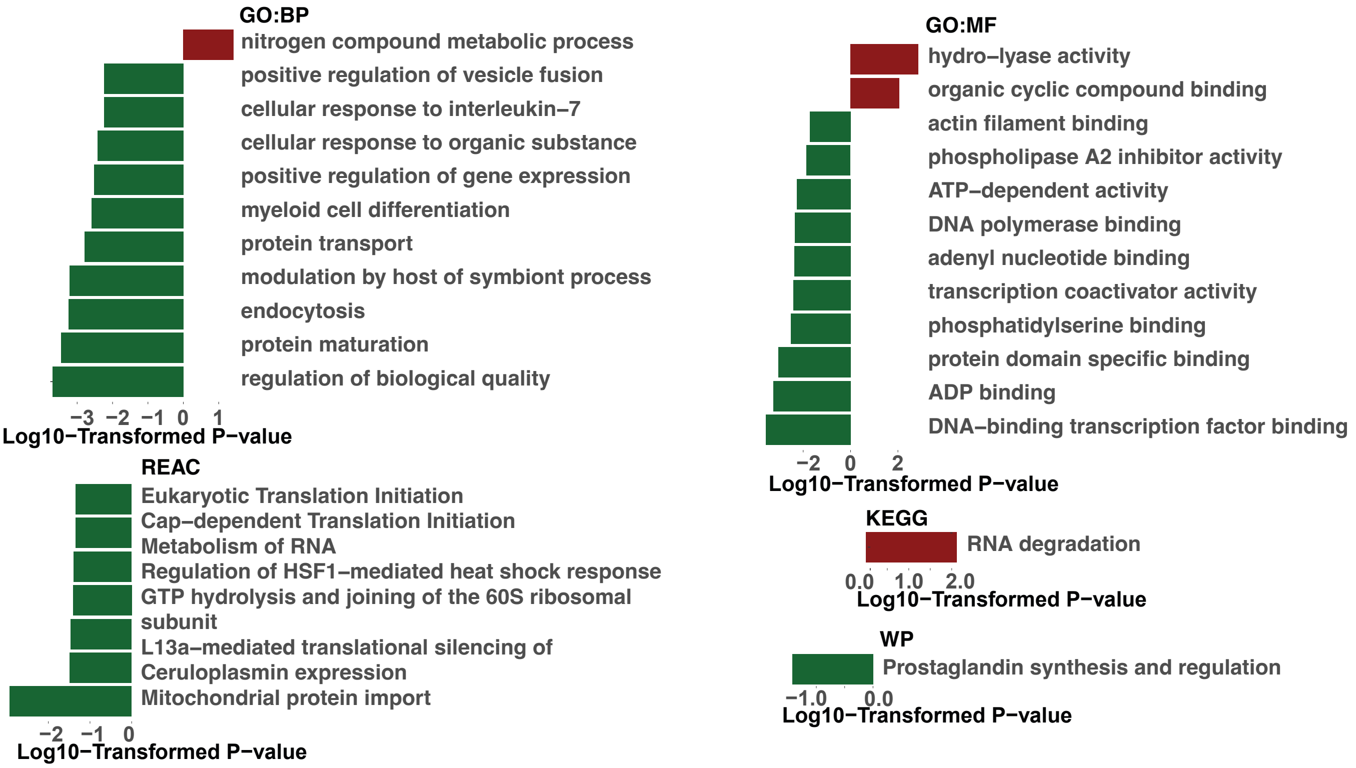

G. C2C12 myotube Acetylomics Pseudosilent

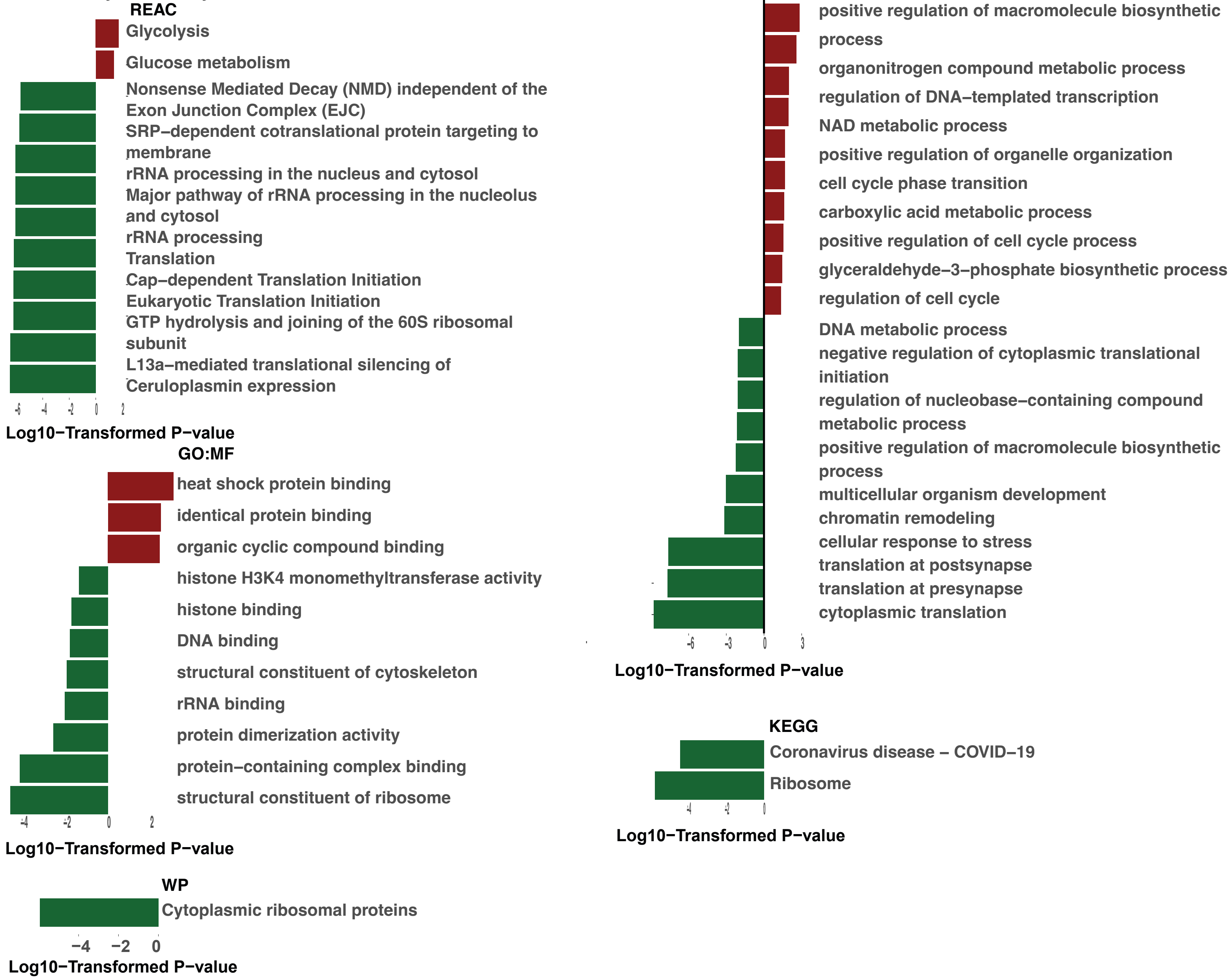

A. C2C12 myotube Phosphoproteomics

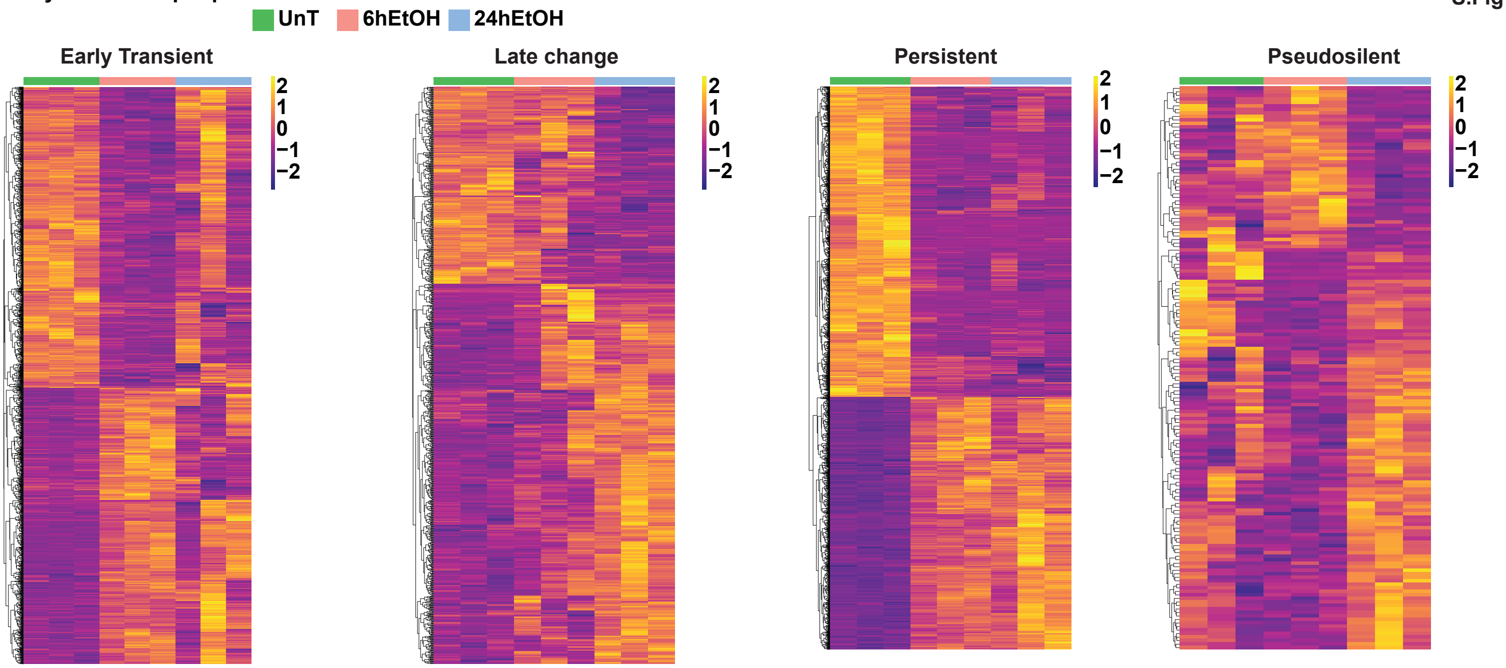

B. C2C12 myotube Phosphoproteomics

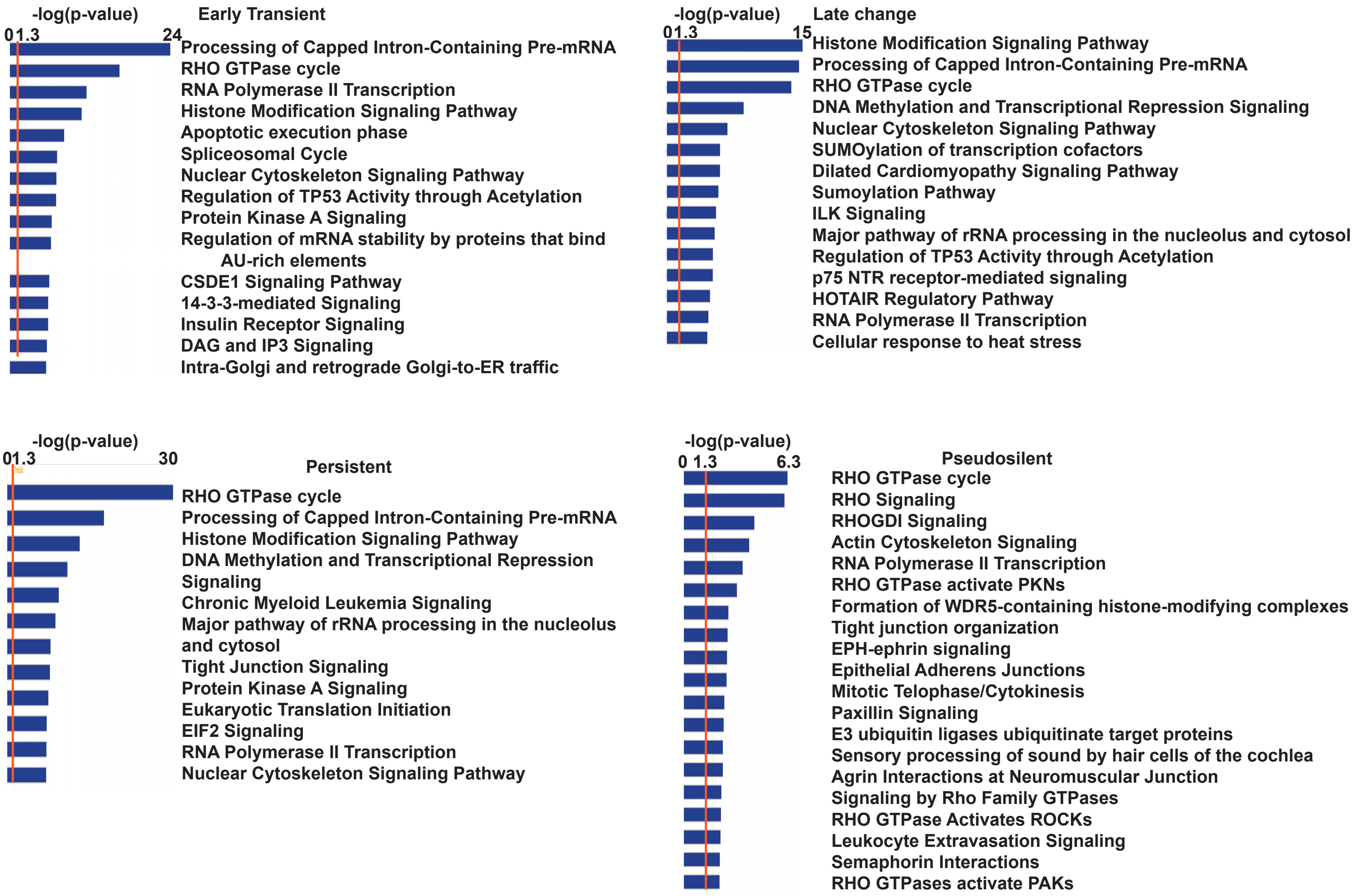

C. C2C12 myotube Phosphoproteomics

S.Fig 7

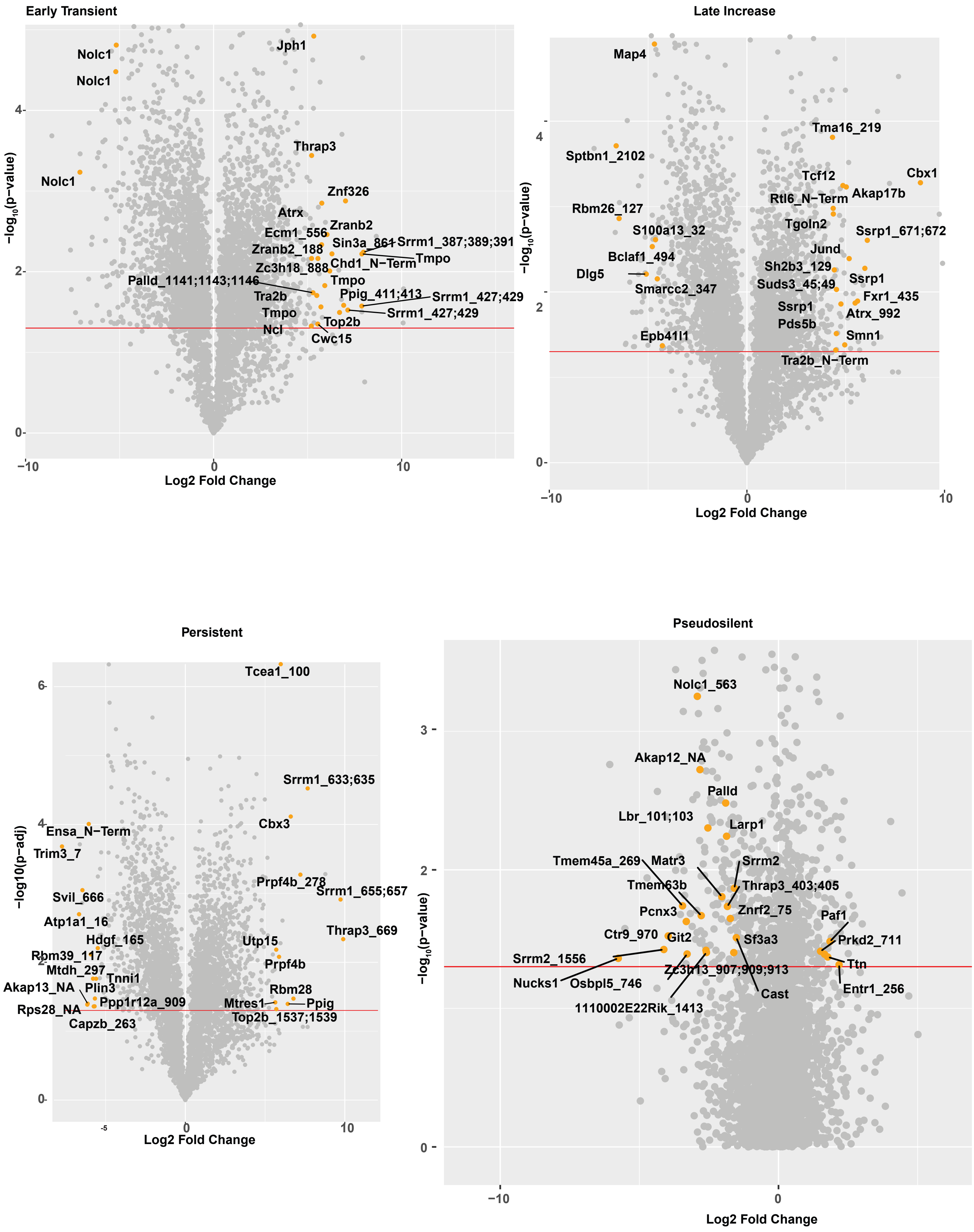

D. C2C12 myotube Phosphoproteomics Early Transient

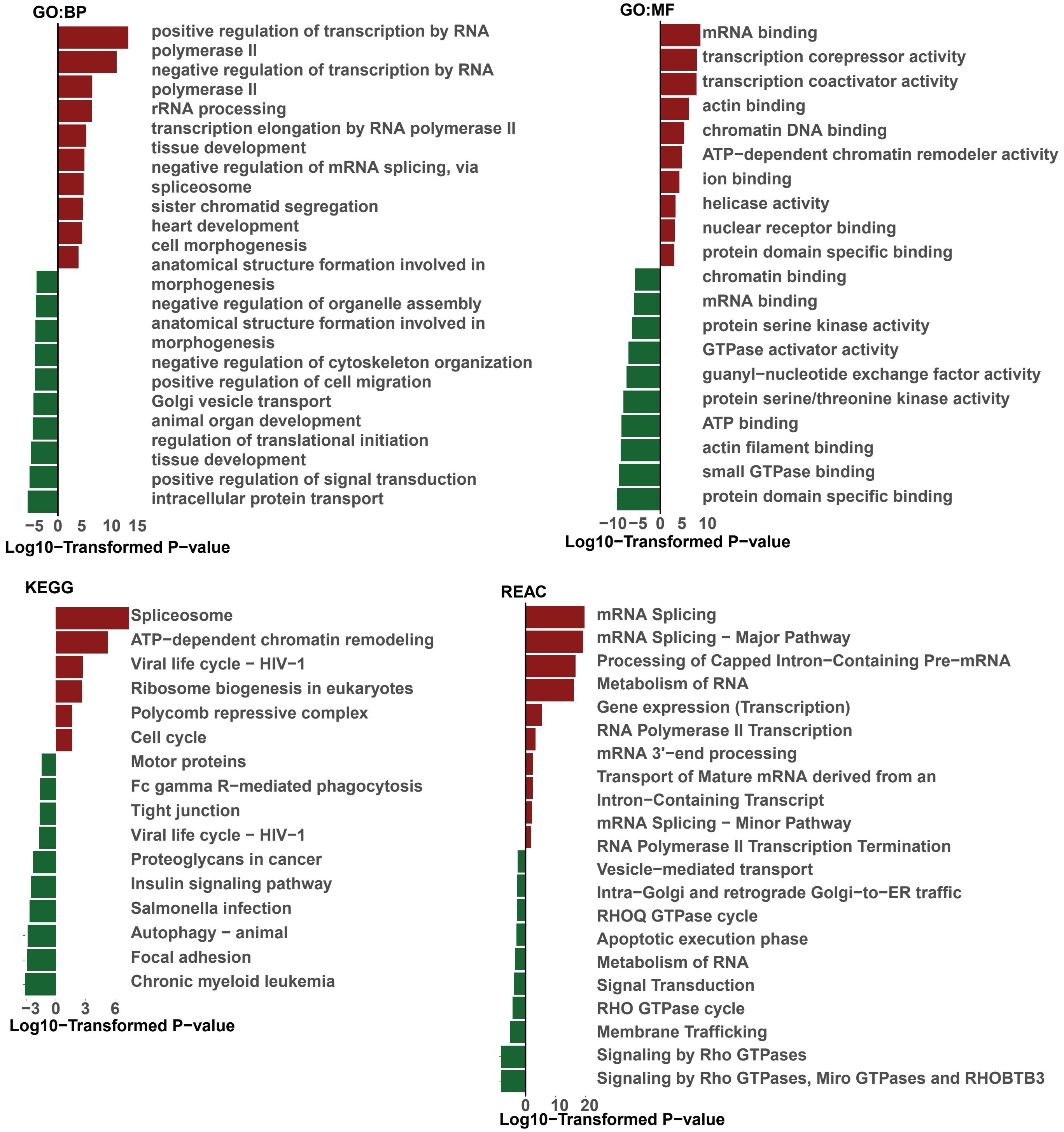

E. C2C12 myotube Phosphoproteomics Late

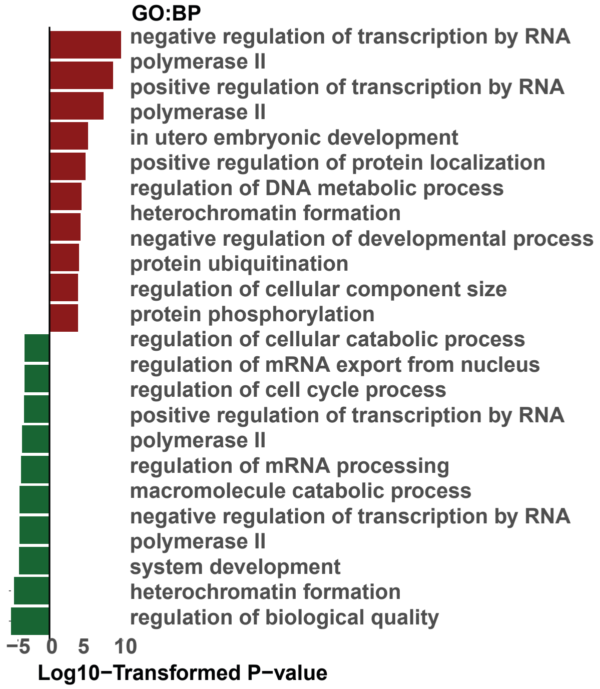

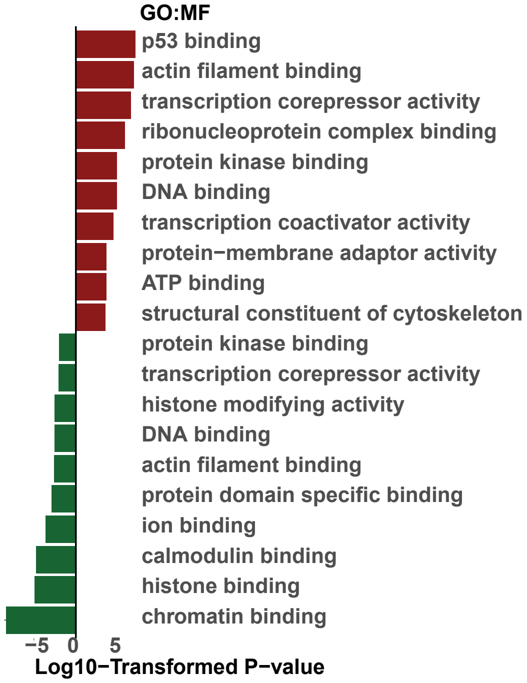

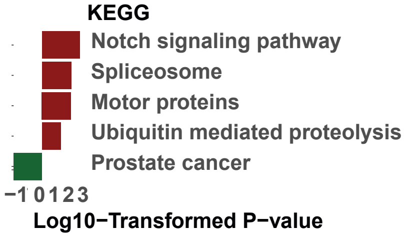

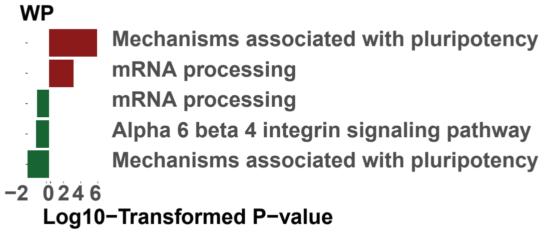

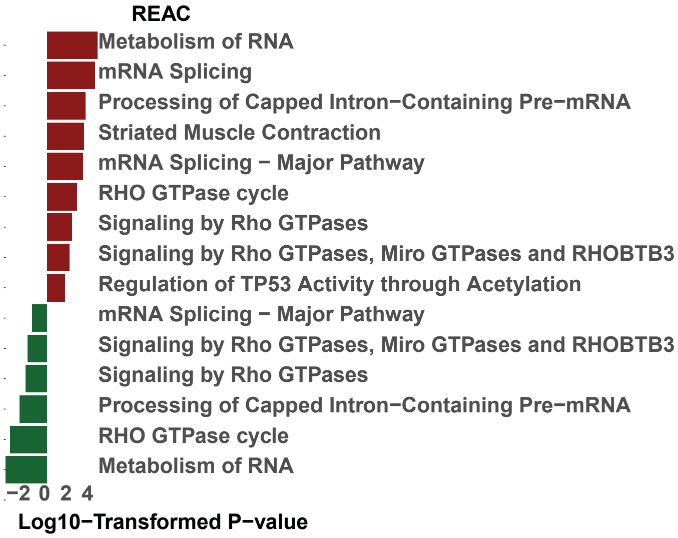

F. C2C12 myotube Phosphoproteomics Persistent

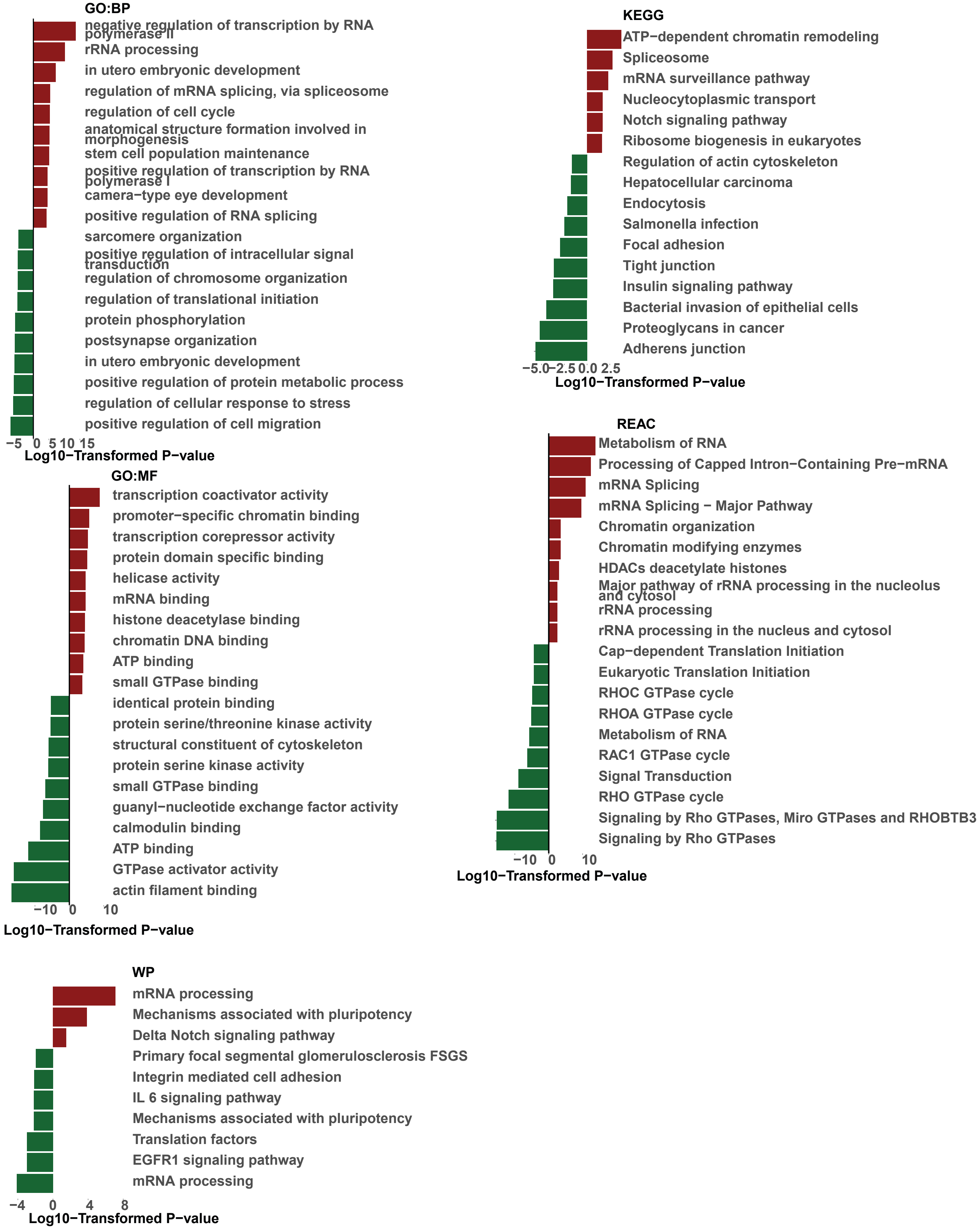

G. C2C12 myotube Phosphoproteomics Pseudosilent

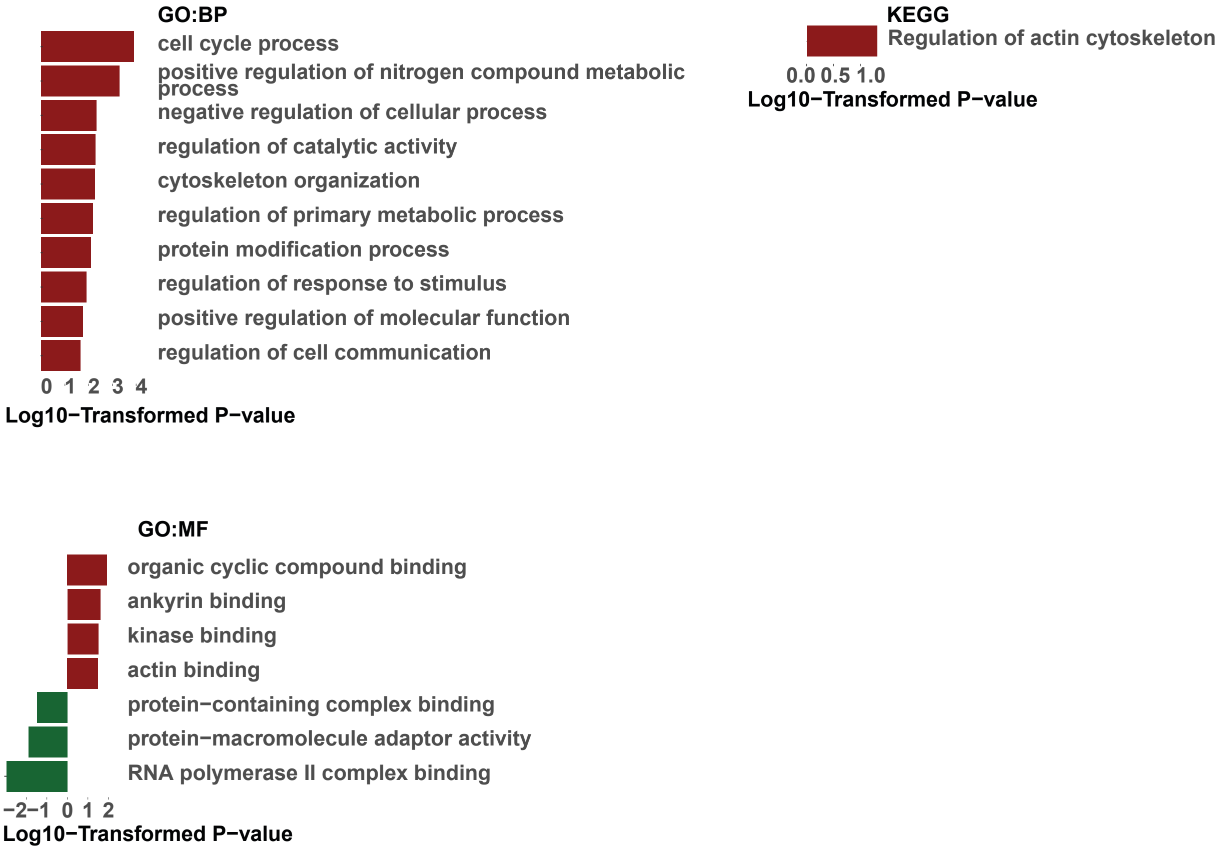

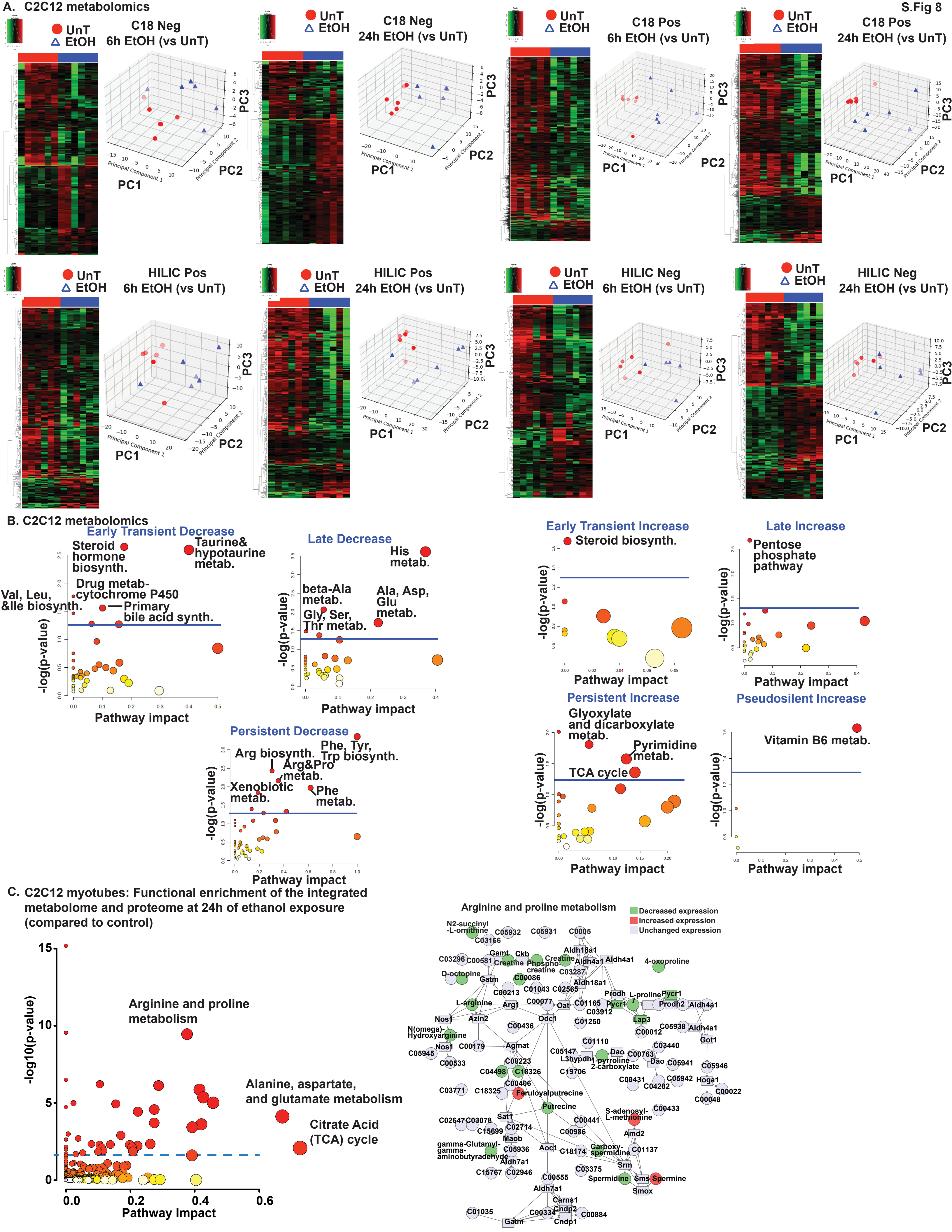

S.Fig 8
A. C2C12 metabolomics
UnT
EtOH
C18 Neg
6h EtOH (vs UnT)
24h EtOH (vs UnT)
C18 Pos
6h EtOH (vs UnT)
24h EtOH (vs UnT)
HILIC Pos
6h EtOH (vs UnT)
24h EtOH (vs UnT)
HILIC Neg
6h EtOH (vs UnT)
24h EtOH (vs UnT)
PC1
PC2
PC3
Principal Component 1
Principal Component 2
B. C2C12 metabolomics
Early Transient Decrease
Steroid hormone biosynth.
Taurine& hypotaurine metab.
Drug metab- cytochrome P450
Val, Leu, &Ile biosynth.
Primary bile acid synth.
Late Decrease
His metab.
beta-Ala metab.
Gly, Ser, Thr metab.
Ala, Asp, Glu metab.
Persistent Decrease
Arg biosynth.
Phe, Tyr, Trp biosynth.
Arg&Pro metab.
Xenobiotic metab.
Phe metab.
Early Transient Increase
Steroid biosynth.
Late Increase
Pentose phosphate pathway
Persistent Increase
Glyoxylate and dicarboxylate metab.
Pyrimidine metab.
TCA cycle
Pseudosilent Increase
Vitamin B6 metab.
-log(p-value)
Pathway impact
C. C2C12 myotubes: Functional enrichment of the integrated metabolome and proteome at 24h of ethanol exposure (compared to control)
Arginine and proline metabolism
Alanine, aspartate, and glutamate metabolism
Citrate Acid (TCA) cycle
-log10(p-value)
Pathway Impact
Arginine and proline metabolism
Decreased expression
Increased expression
Unchanged expression

S.Fig 8

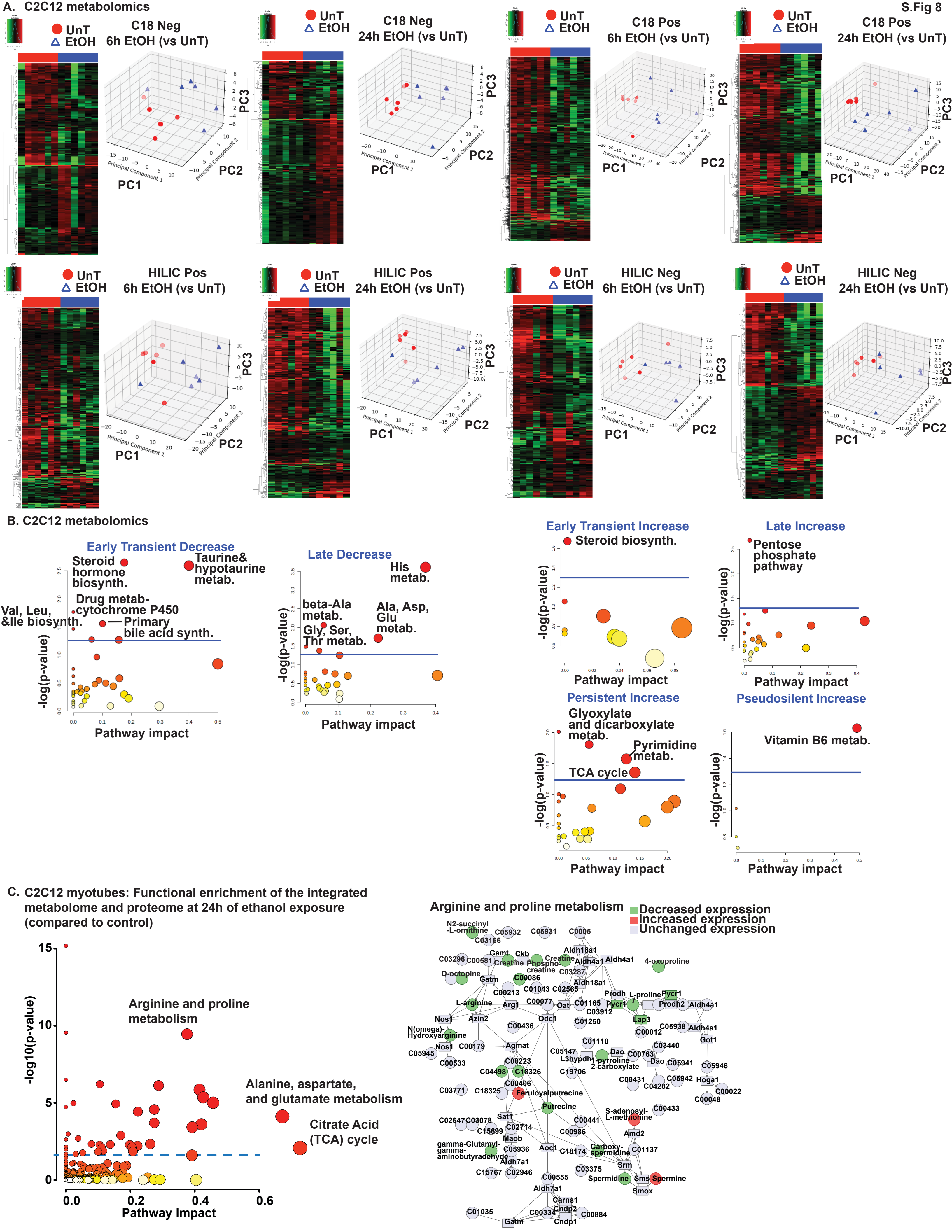

A. C2C12 metabolomics
UnT
EtOH
C18 Neg
6h EtOH (vs UnT)
24h EtOH (vs UnT)
C18 Pos
6h EtOH (vs UnT)
24h EtOH (vs UnT)
HILIC Pos
6h EtOH (vs UnT)
24h EtOH (vs UnT)
HILIC Neg
6h EtOH (vs UnT)
24h EtOH (vs UnT)
PC1
PC2
PC3
Principal Component 1
Principal Component 2
B. C2C12 metabolomics
Early Transient Decrease
Steroid hormone biosynth.
Taurine& hypotaurine metab.
Drug metab-cytochrome P450
Val, Leu, &Ile biosynth.
Primary bile acid synth.
Late Decrease
His metab.
beta-Ala metab.
Gly, Ser, Thr metab.
Ala, Asp, Glu metab.
Early Transient Increase
Steroid biosynth.
Late Increase
Pentose phosphate pathway
Persistent Increase
Glyoxylate and dicarboxylate metab.
Pyrimidine metab.
TCA cycle
Pseudosilent Increase
Vitamin B6 metab.
-log(p-value)
Pathway impact
C. C2C12 myotubes: Functional enrichment of the integrated metabolome and proteome at 24h of ethanol exposure (compared to control)
Arginine and proline metabolism
Alanine, aspartate, and glutamate metabolism
Citrate Acid (TCA) cycle
-log10(p-value)
Pathway Impact
Arginine and proline metabolism
Decreased expression
Increased expression
Unchanged expression

A. Mouse skeletal muscle RNAseq

S.Fig 9

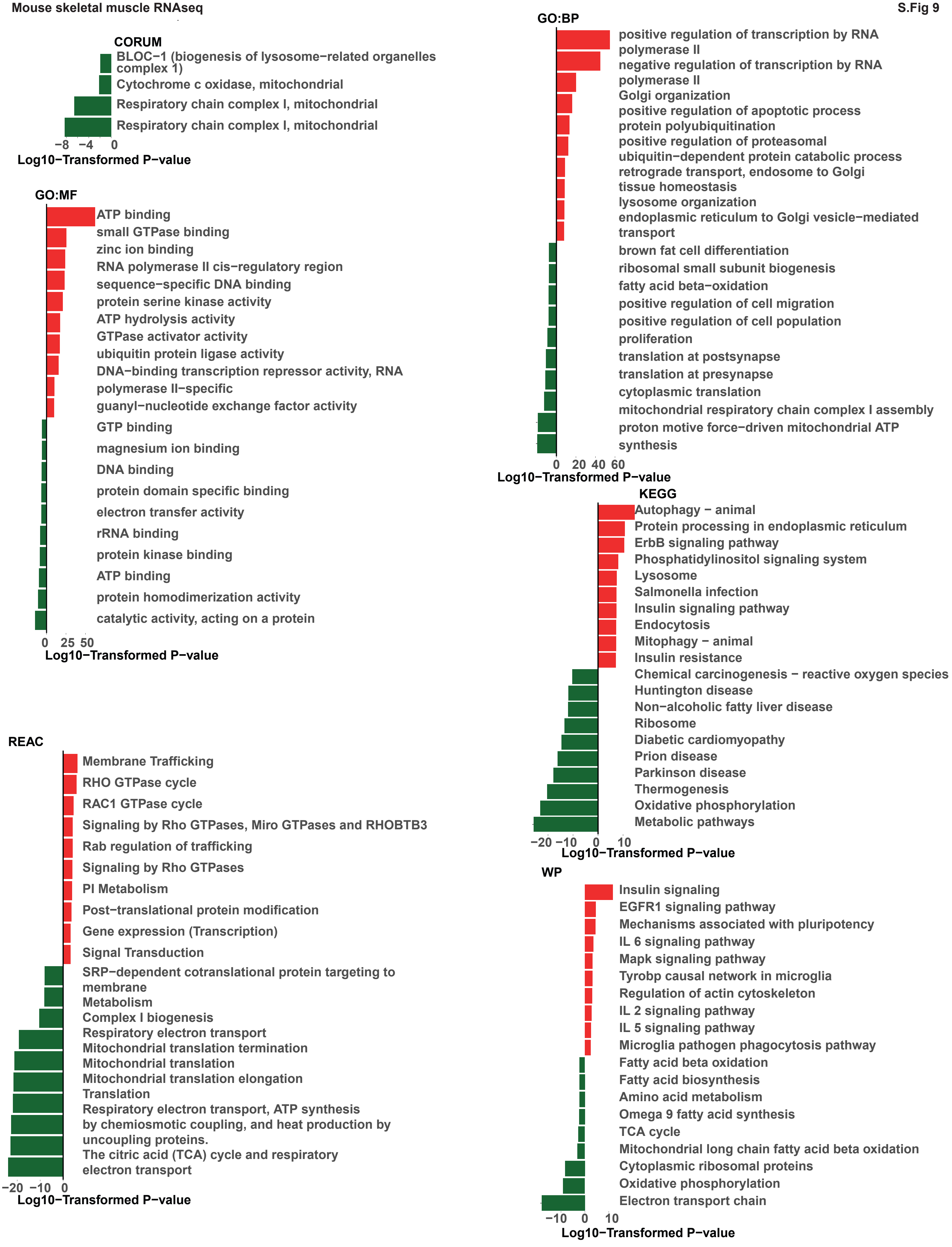

S.Fig 9

B. Mouse skeletal muscle proteomics

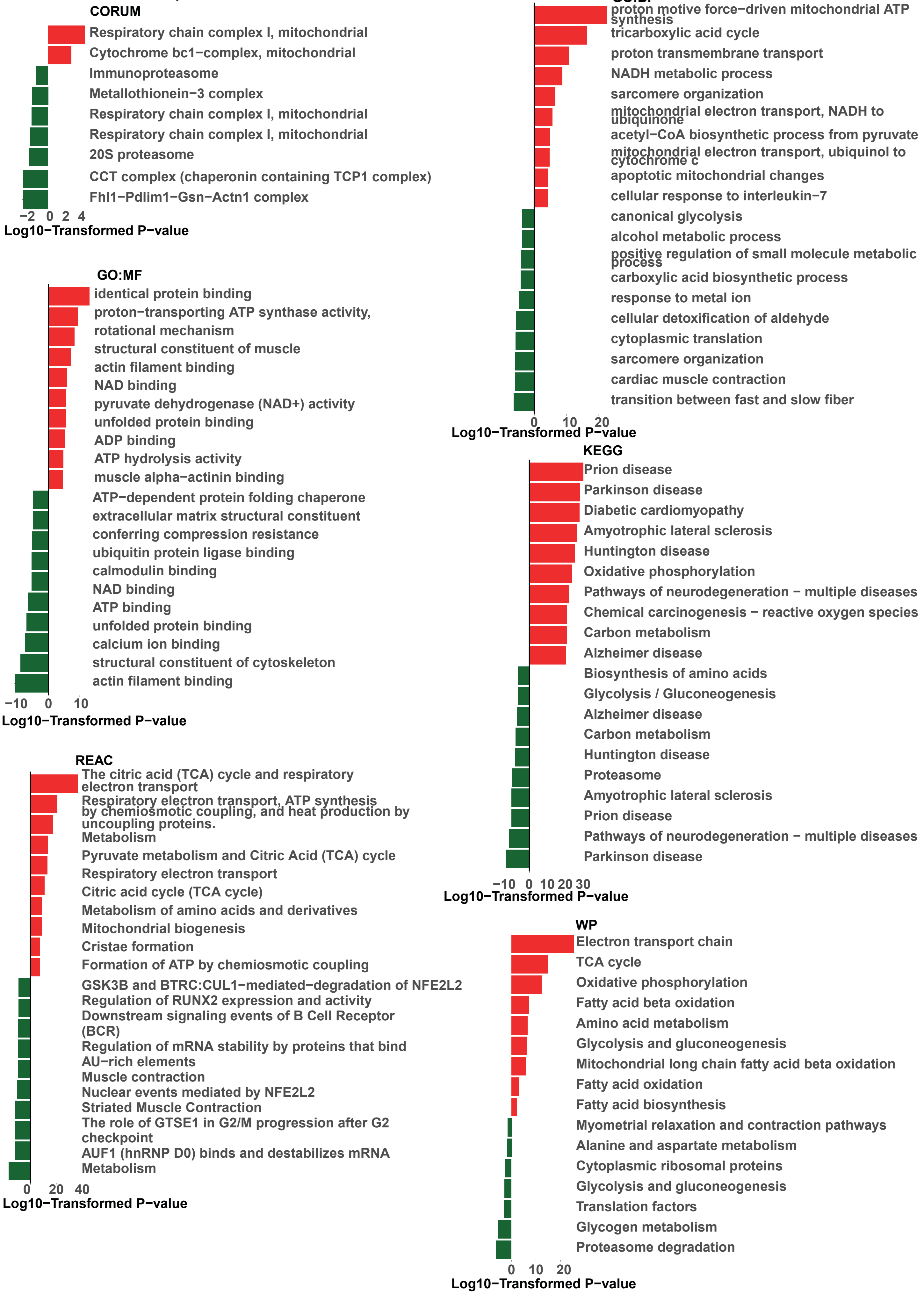

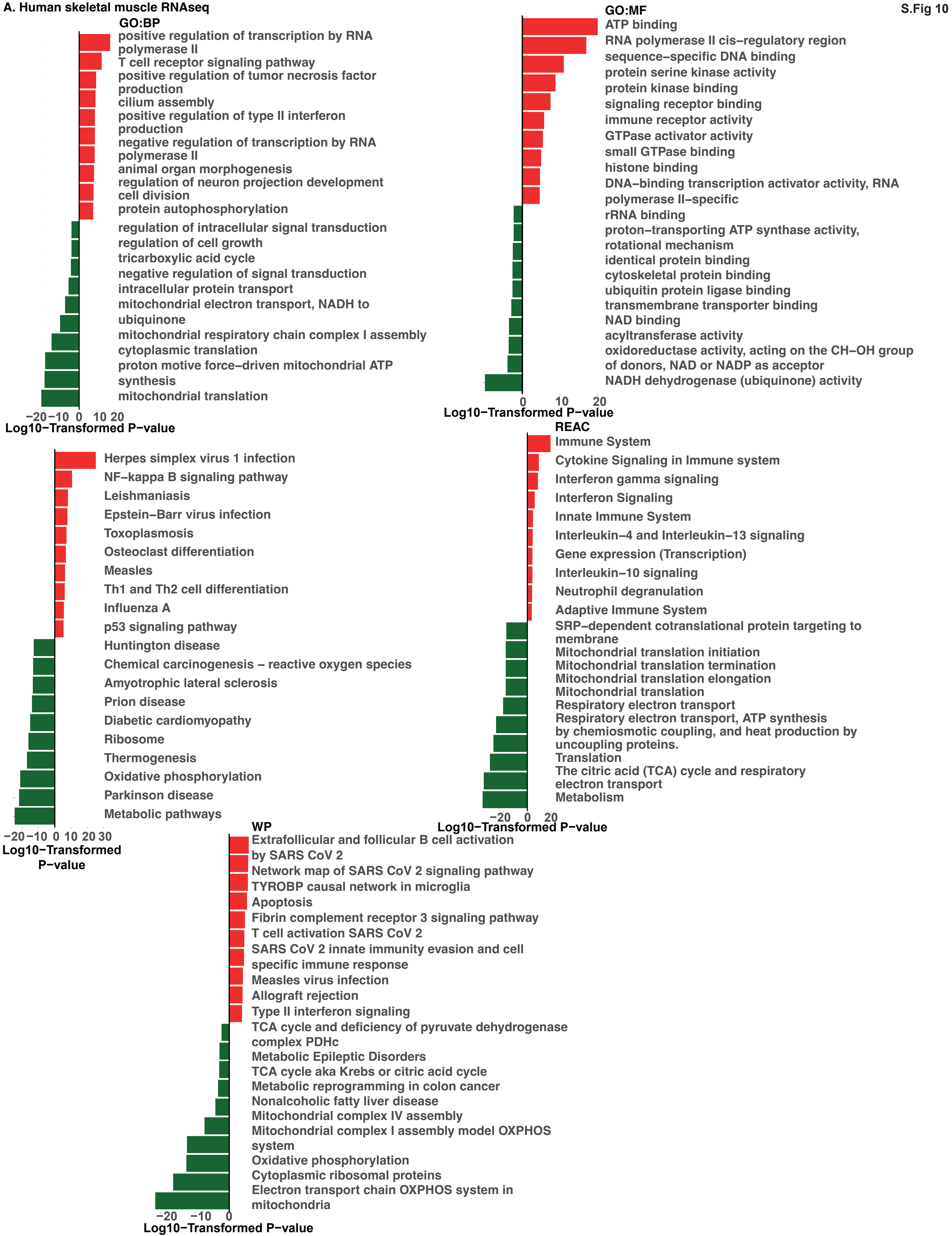
S.Fig 10
A. Human skeletal muscle RNAseq
GO:BP
positive regulation of transcription by RNA polymerase II
T cell receptor signaling pathway
positive regulation of tumor necrosis factor production
cilium assembly
positive regulation of type II interferon production
negative regulation of transcription by RNA polymerase II
animal organ morphogenesis
regulation of neuron projection development
cell division
protein autophosphorylation
regulation of intracellular signal transduction
regulation of cell growth
tricarboxylic acid cycle
negative regulation of signal transduction
intracellular protein transport
mitochondrial electron transport, NADH to ubiquinone
mitochondrial respiratory chain complex I assembly
cytoplasmic translation
proton motive force−driven mitochondrial ATP synthesis
mitochondrial translation
−20 −10 0 10 20
Log10−Transformed P−value
GO:MF
ATP binding
RNA polymerase II cis−regulatory region
sequence−specific DNA binding
protein serine kinase activity
protein kinase binding
signaling receptor binding
immune receptor activity
GTPase activator activity
small GTPase binding
histone binding
DNA−binding transcription activator activity, RNA polymerase II−specific
rRNA binding
proton−transporting ATP synthase activity, rotational mechanism
identical protein binding
cytoskeletal protein binding
ubiquitin protein ligase binding
transmembrane transporter binding
NAD binding
acyltransferase activity
oxidoreductase activity, acting on the CH−OH group of donors, NAD or NADP as acceptor
NADH dehydrogenase (ubiquinone) activity
0 10 20
Log10−Transformed P−value
Herpes simplex virus 1 infection
NF−kappa B signaling pathway
Leishmaniasis
Epstein−Barr virus infection
Toxoplasmosis
Osteoclast differentiation
Measles
Th1 and Th2 cell differentiation
Influenza A
p53 signaling pathway
Huntington disease
Chemical carcinogenesis − reactive oxygen species
Amyotrophic lateral sclerosis
Prion disease
Diabetic cardiomyopathy
Ribosome
Thermogenesis
Oxidative phosphorylation
Parkinson disease
Metabolic pathways
−20 −10 0 10 20 30
Log10−Transformed P−value
REAC
Immune System
Cytokine Signaling in Immune system
Interferon gamma signaling
Interferon Signaling
Innate Immune System
Interleukin−4 and Interleukin−13 signaling
Gene expression (Transcription)
Interleukin−10 signaling
Neutrophil degranulation
Adaptive Immune System
SRP−dependent cotranslational protein targeting to membrane
Mitochondrial translation initiation
Mitochondrial translation termination
Mitochondrial translation elongation
Mitochondrial translation
Respiratory electron transport
Respiratory electron transport, ATP synthesis by chemiosmotic coupling, and heat production by uncoupling proteins.
Translation
The citric acid (TCA) cycle and respiratory electron transport
Metabolism
−20 0 20
Log10−Transformed P−value
WP
Extrafollicular and follicular B cell activation by SARS CoV 2
Network map of SARS CoV 2 signaling pathway
TYROBP causal network in microglia
Apoptosis
Fibrin complement receptor 3 signaling pathway
T cell activation SARS CoV 2
SARS CoV 2 innate immunity evasion and cell specific immune response
Measles virus infection
Allograft rejection
Type II interferon signaling
TCA cycle and deficiency of pyruvate dehydrogenase complex PDHc
Metabolic Epileptic Disorders
TCA cycle aka Krebs or citric acid cycle
Metabolic reprogramming in colon cancer
Nonalcoholic fatty liver disease
Mitochondrial complex IV assembly
Mitochondrial complex I assembly model OXPHOS system
Oxidative phosphorylation
Cytoplasmic ribosomal proteins
Electron transport chain OXPHOS system in mitochondria
−20 −10 0
Log10−Transformed P−value

B. Human skeletal muscle proteomics

S.Fig 10

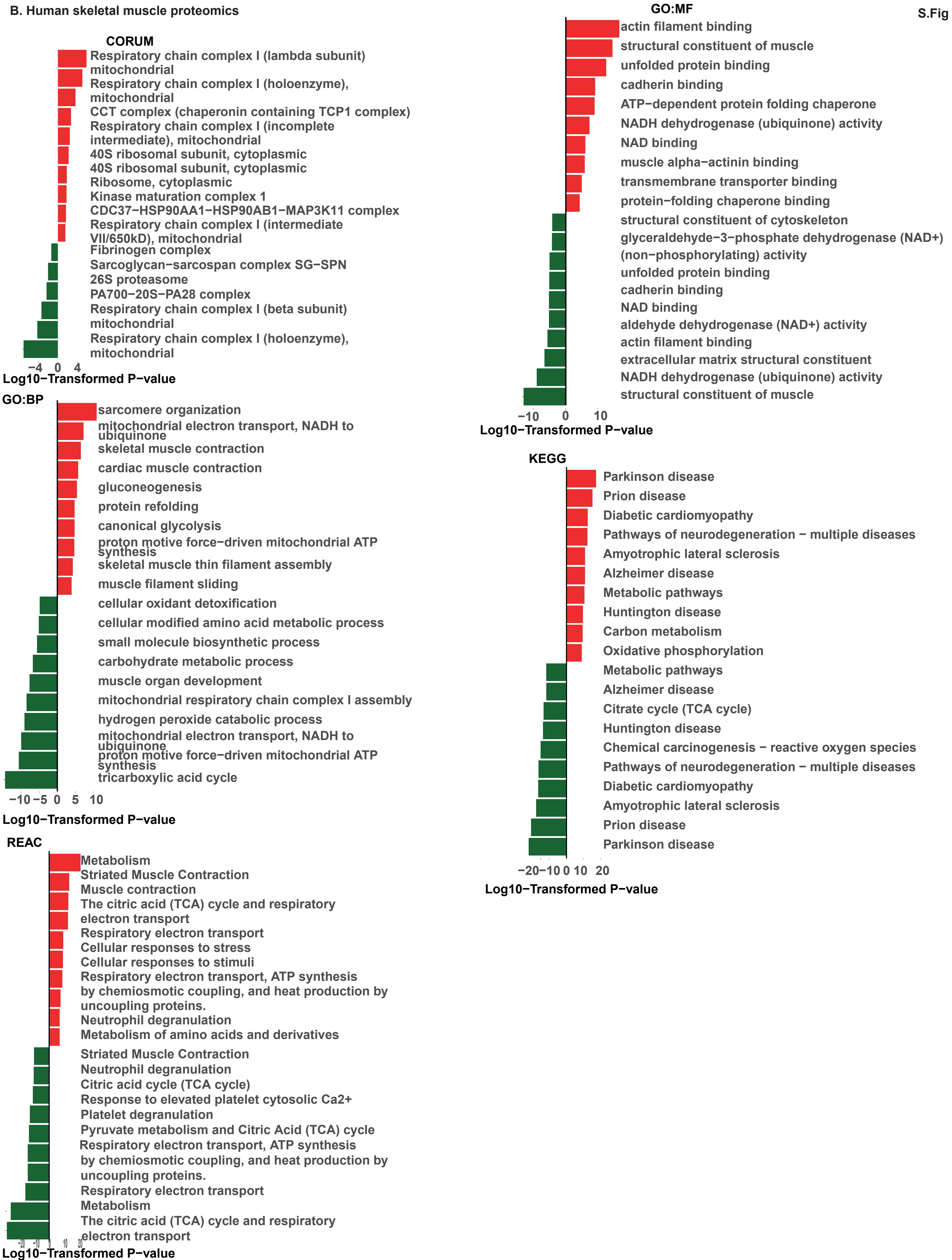

A. Persistent

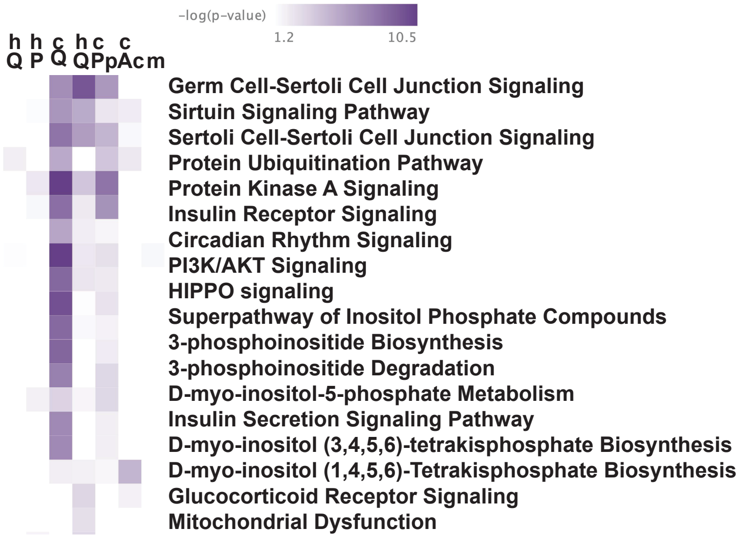

B. Pseudosilent

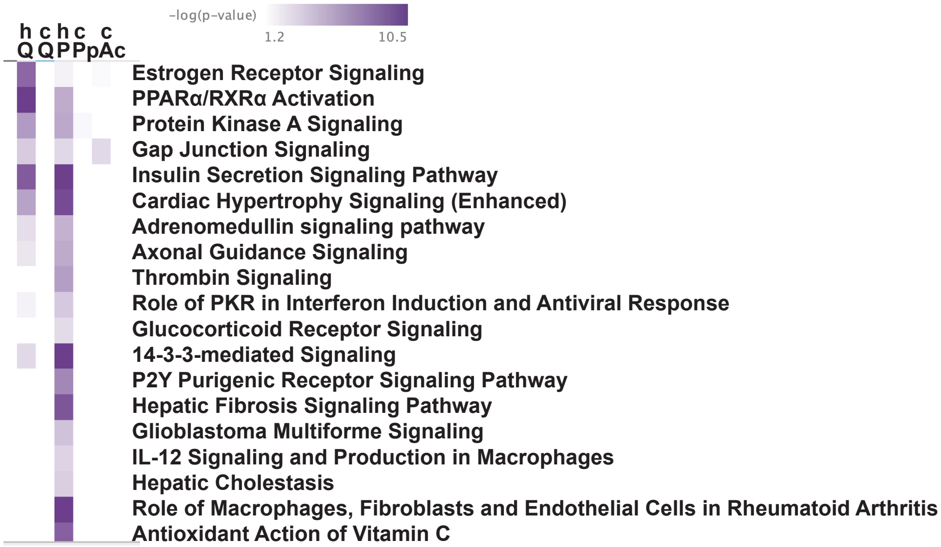

A. Top 100 Senescence Genes

S.Fig 12

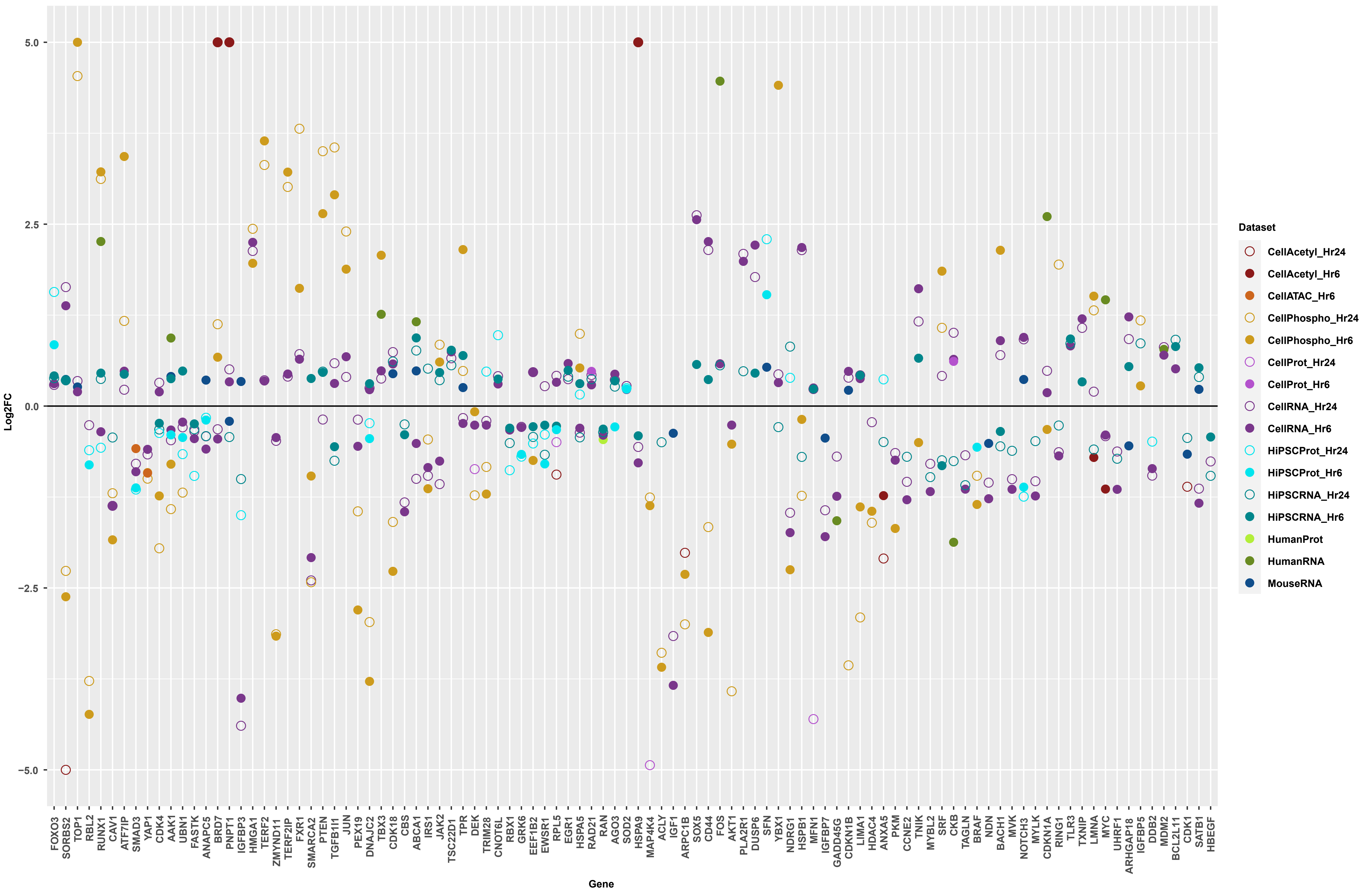

B. TCA Cycle Genes: Most in Same Direction & Average FC

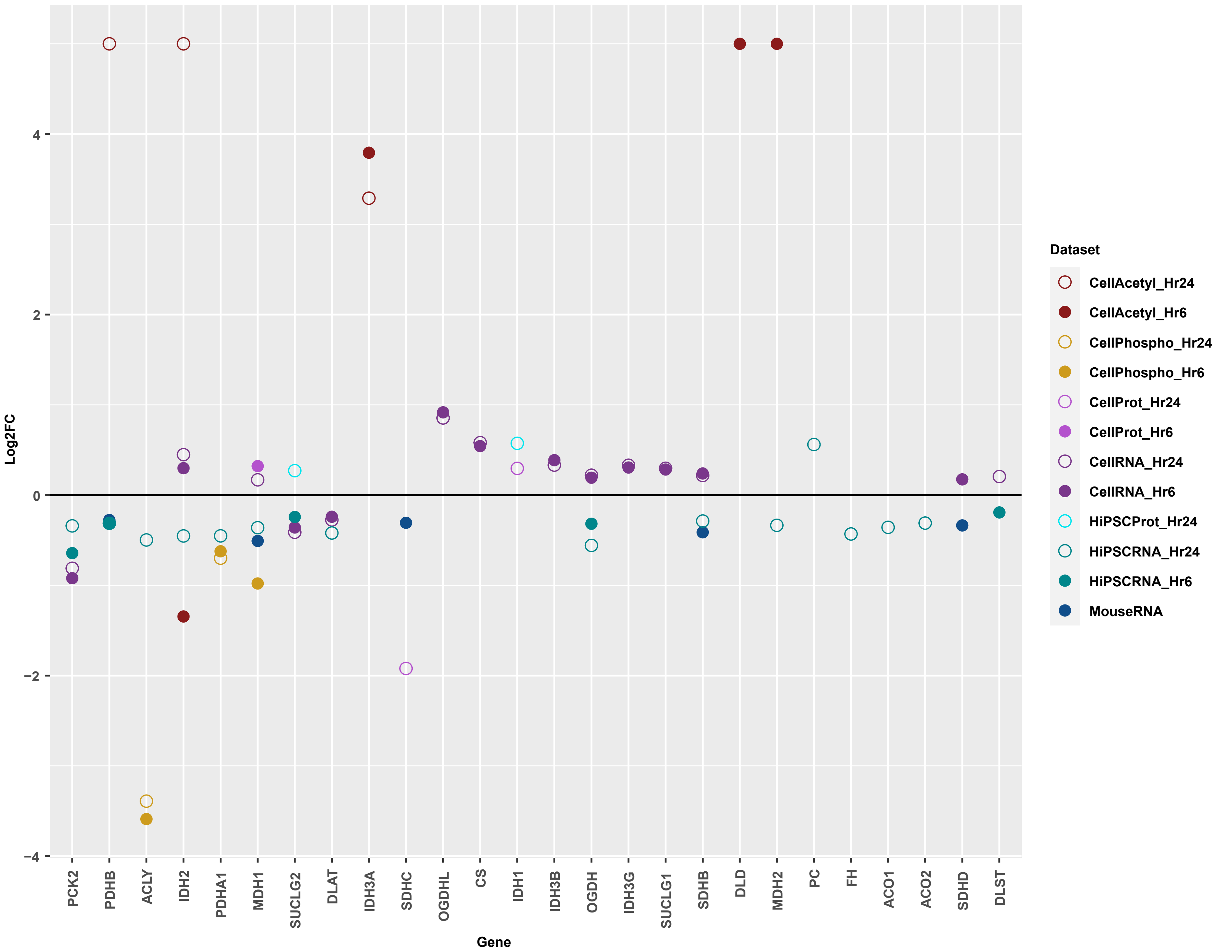

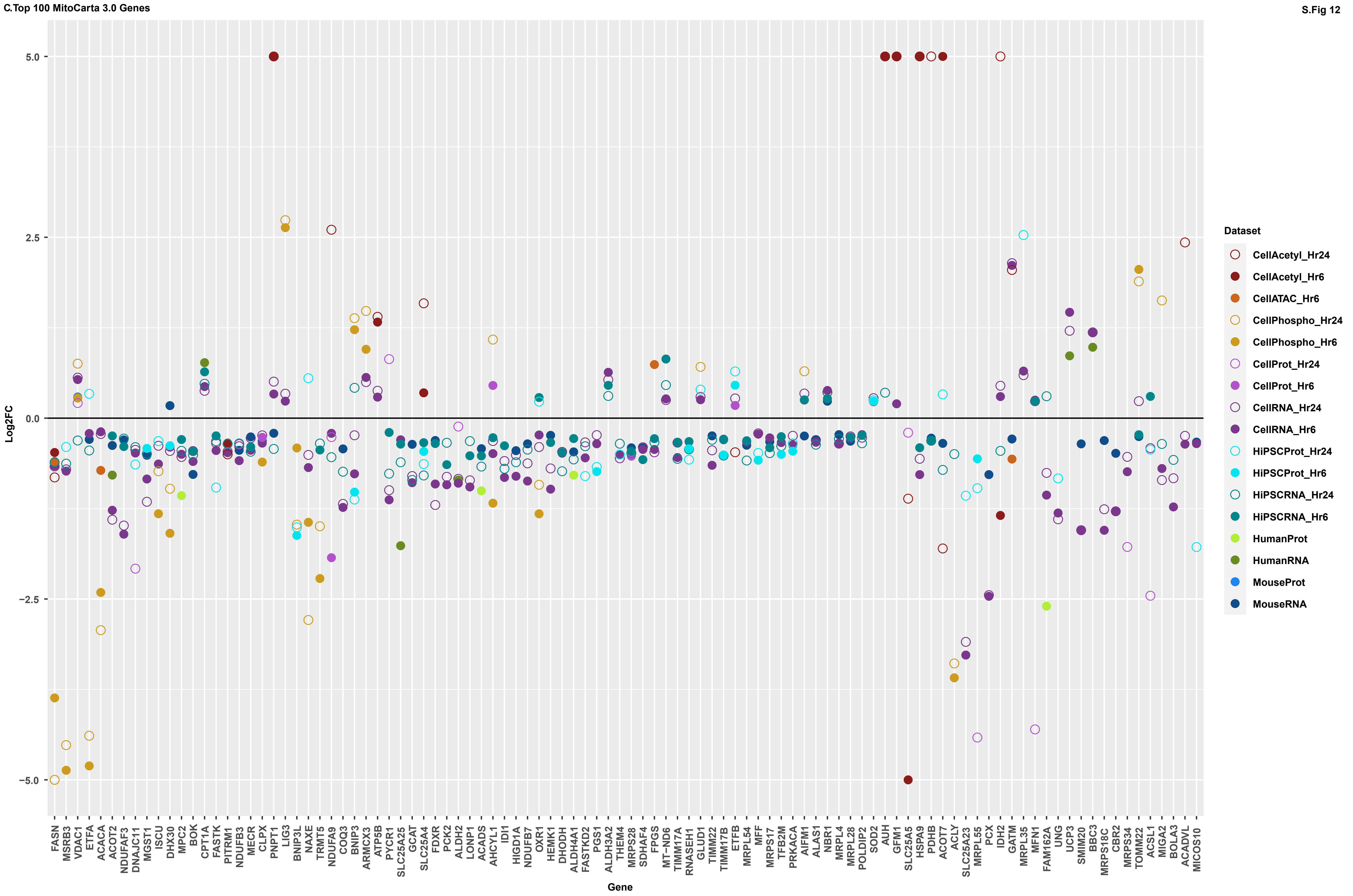

C.Top 100 MitoCarta 3.0 Genes
Log2FC
5.0
2.5
0.0
-2.5
-5.0
Gene
FASN
MSRB3
VDAC1
ETFA
ACACA
ACOT2
NDUFAF3
DNAJC11
MGST1
ISCU
DHX30
MPC2
BOK
CPT1A
FASTK
PITRM1
NDUFB3
MECR
CLPX
PNPT1
LIG3
BNIP3L
NAXE
TRMT5
NDUFA9
COQ3
BNIP3
ARMCX3
ATP5B
PYCR1
SLC25A25
GCAT
SLC25A4
FDXR
PCK2
ALDH2
LONP1
ACADS
AHCYL1
IDI1
HIGD1A
NDUFB7
OXR1
HEMK1
DHODH
ALDH4A1
FASTKD2
PGS1
ALDH3A2
THEM4
MRPS28
SDHAF4
FPGS
MT−ND6
TIMM17A
RNASEH1
GLUD1
TIMM22
TIMM17B
ETFB
MRPL54
MFF
MRPS17
TFB2M
PRKACA
AIFM1
ALAS1
NBR1
MRPL4
MRPL28
POLDIP2
SOD2
AUH
GFM1
SLC25A5
HSPA9
PDHB
ACOT7
ACLY
SLC25A23
MRPL55
PCX
IDH2
GATM
MRPL35
MFN1
FAM162A
UNG
UCP3
SMIM20
BBC3
MRPS18C
CBR2
MRPS34
TOMM22
ACSL1
MIGA2
BOLA3
ACADVL
MICOS10
Dataset
CellAcetyl_Hr24
CellAcetyl_Hr6
CellATAC_Hr6
CellPhospho_Hr24
CellPhospho_Hr6
CellProt_Hr24
CellProt_Hr6
CellRNA_Hr24
CellRNA_Hr6
HiPSCProt_Hr24
HiPSCProt_Hr6
HiPSCRNA_Hr24
HiPSCRNA_Hr6
HumanProt
HumanRNA
MouseProt
MouseRNA

D. Top 100 HIF1α Signaling Genes

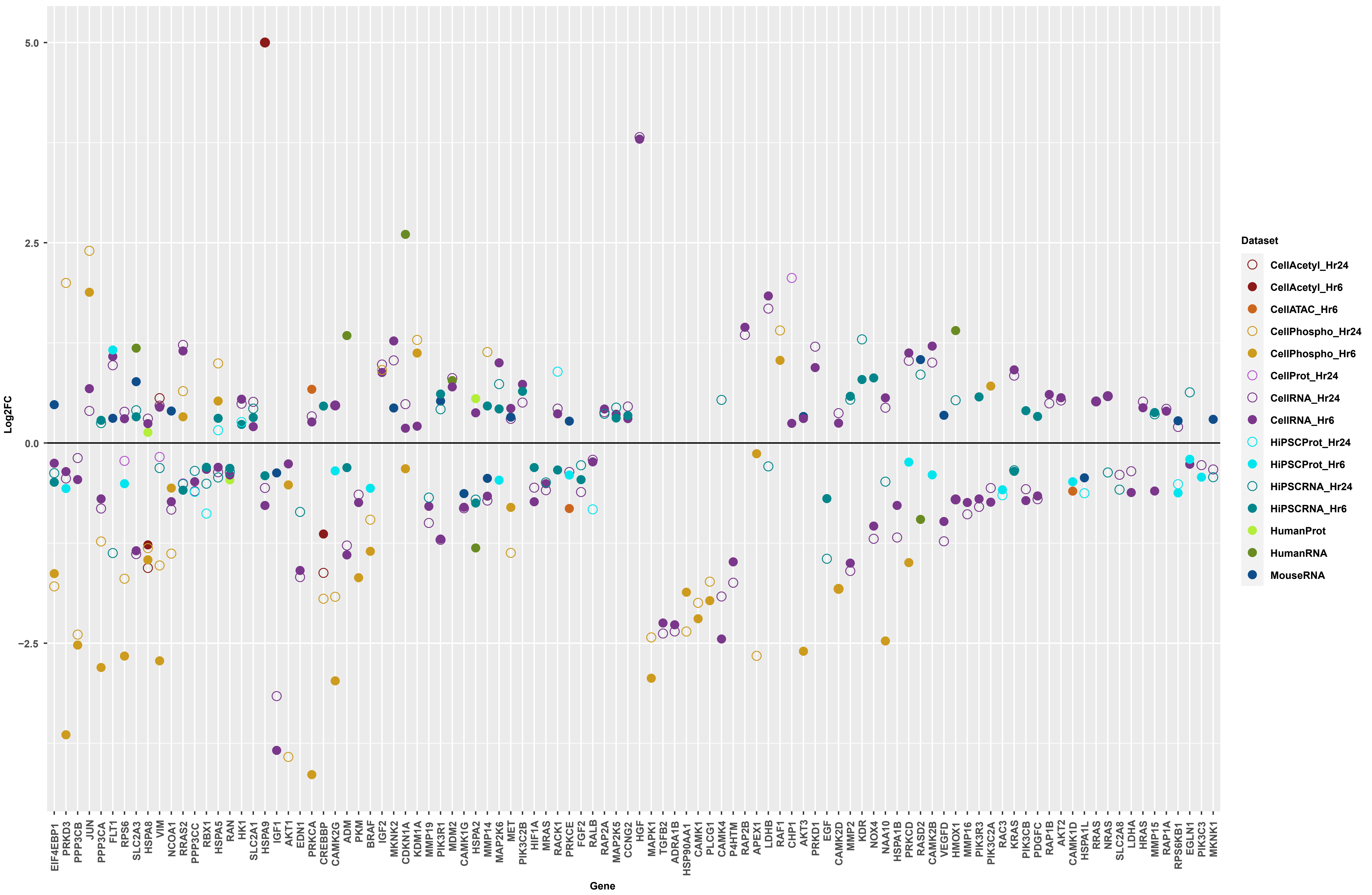

S.Fig 13

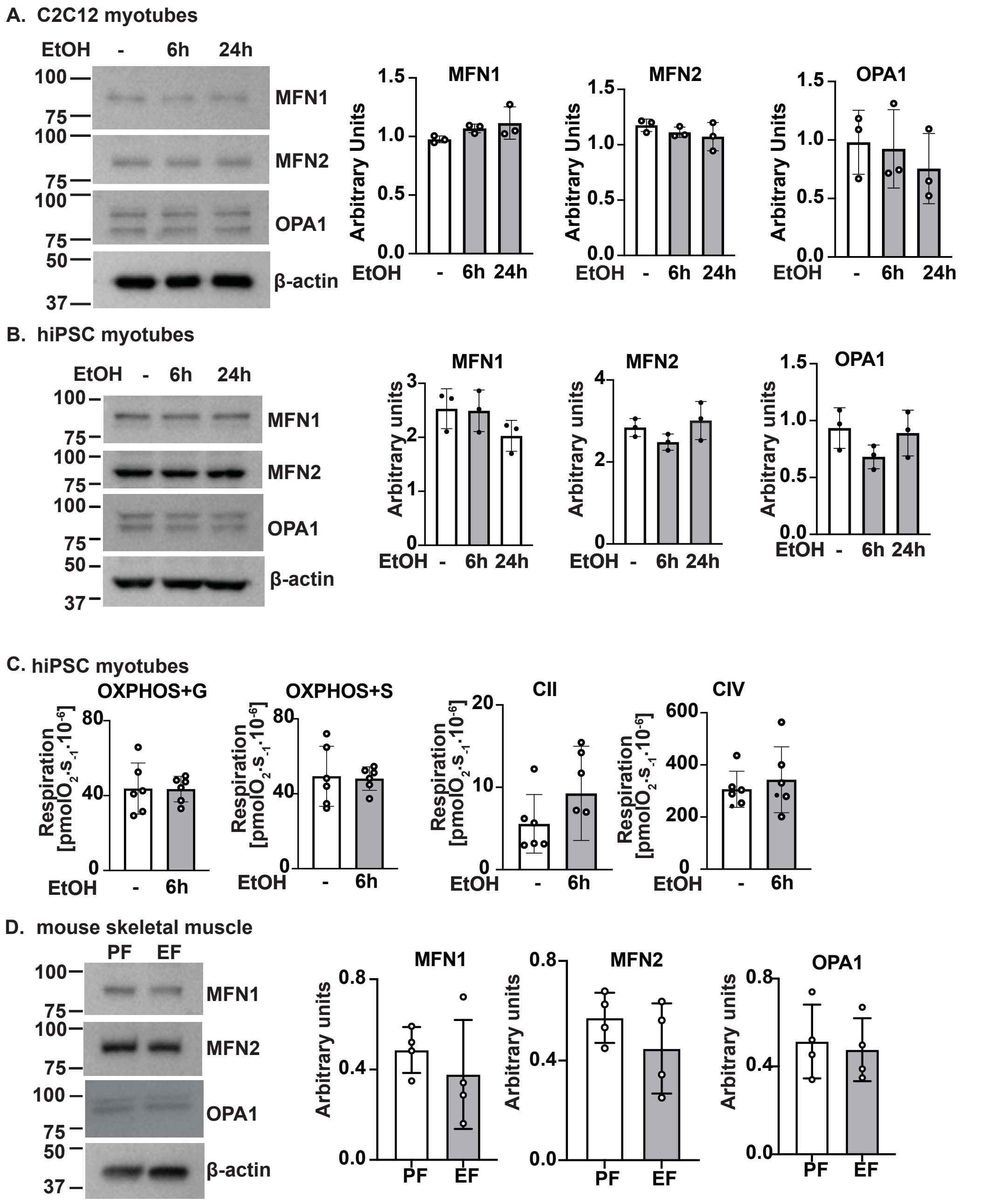
A. C2C12 myotubes
EtOH - 6h 24h
MFN1
MFN2
OPA1
β-actin
100
75
50
37
Arbitrary Units
B. hiPSC myotubes
Arbitrary units
C. hiPSC myotubes
OXPHOS+G
OXPHOS+S
CII
CIV
Respiration [pmolO2·s-1·10-6]
D. mouse skeletal muscle
PF EF

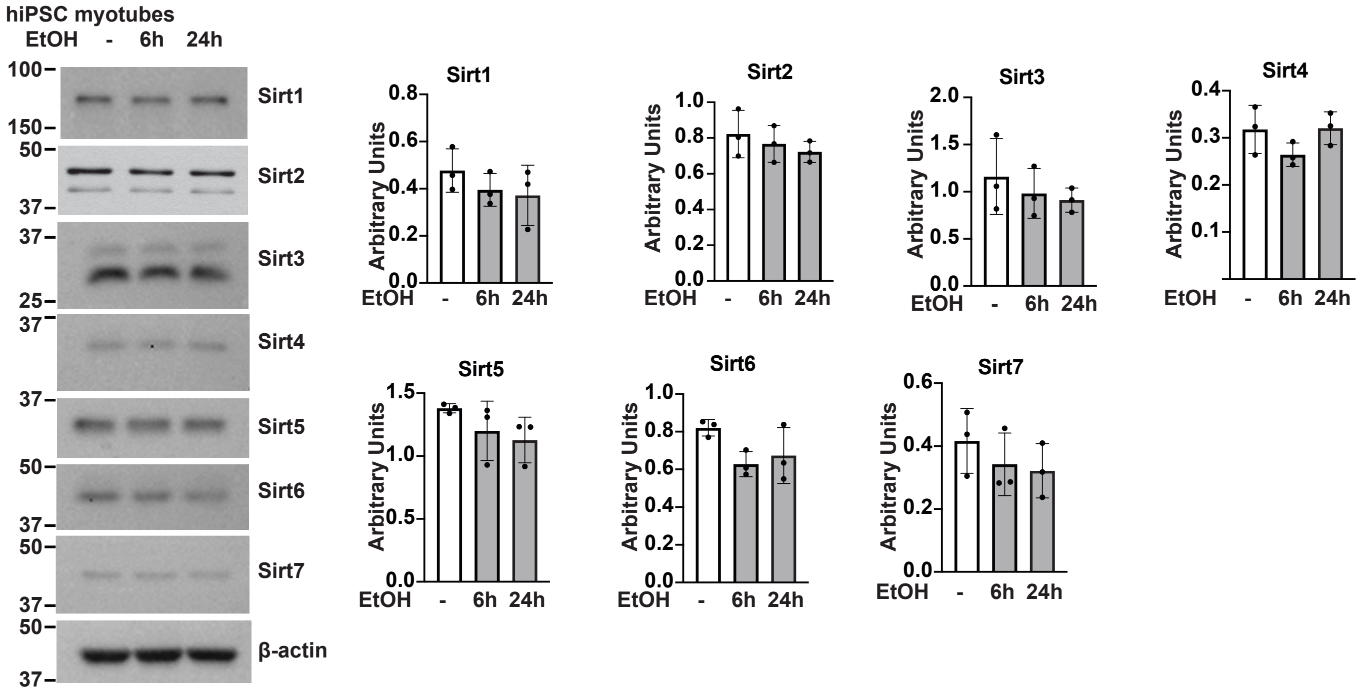
hiPSC myotubes
EtOH - 6h 24h
100
150
50
37
37
25
37
37
50
37
50
37
50
37
Sirt1
Sirt2
Sirt3
Sirt4
Sirt5
Sirt6
Sirt7
β-actin
Sirt1
Sirt2
Sirt3
Sirt4
Sirt5
Sirt6
Sirt7
Arbitrary Units
EtOH - 6h 24h

S.Fig 15

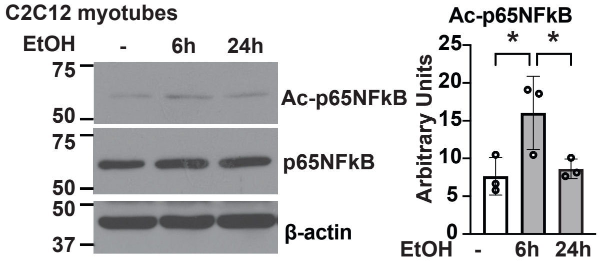
C2C12 myotubes
EtOH
-
6h
24h
75
50
Ac-p65NFkB
75
50
p65NFkB
50
37
β-actin
Ac-p65NFkB
*
*
Arbitrary Units
25
20
15
10
5
0
EtOH
-
6h
24h

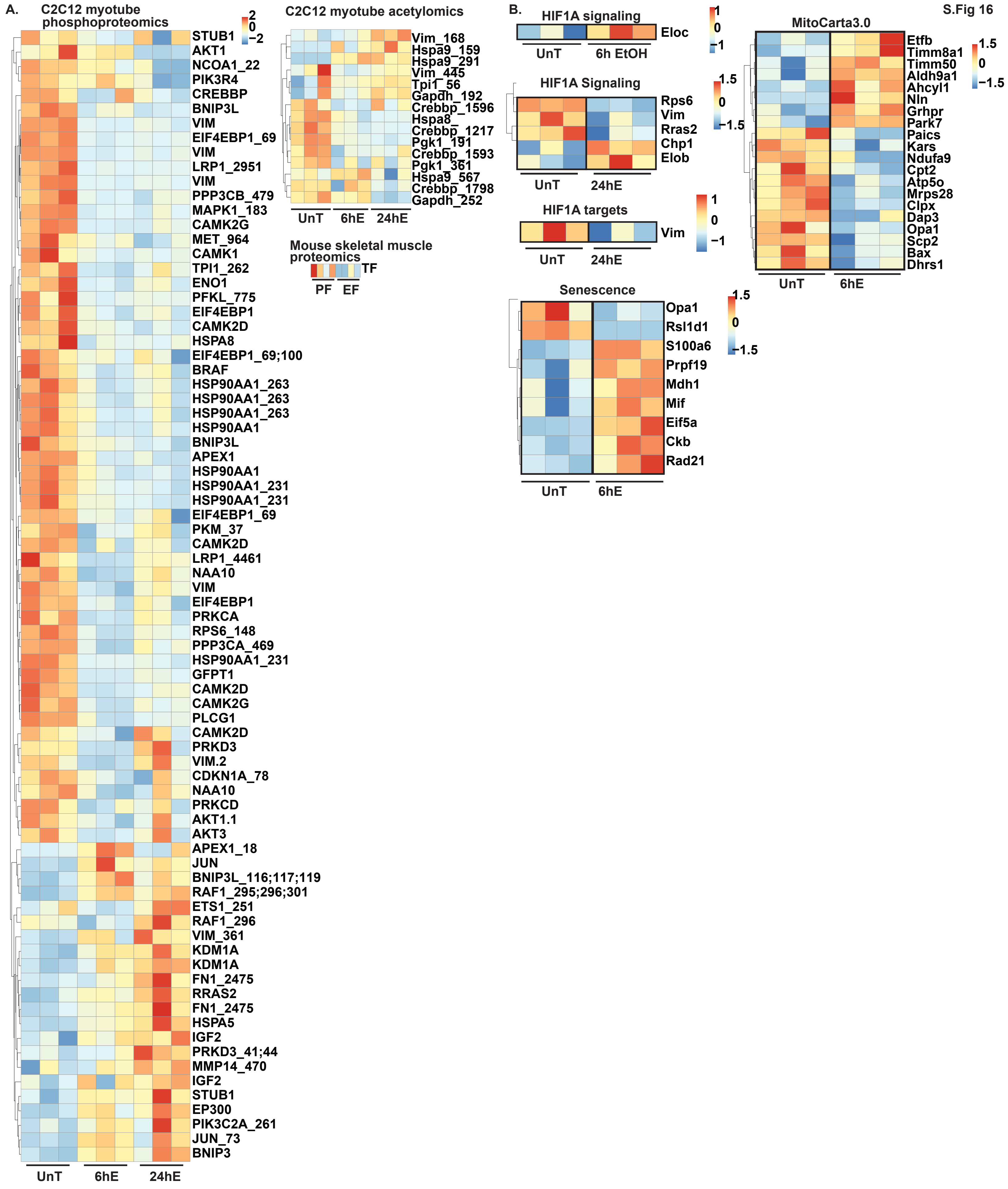
S.Fig 16
A.
C2C12 myotube phosphoproteomics
2
0
−2
STUB1
AKT1
NCOA1_22
PIK3R4
CREBBP
BNIP3L
VIM
EIF4EBP1_69
VIM
LRP1_2951
VIM
PPP3CB_479
MAPK1_183
CAMK2G
MET_964
CAMK1
TPI1_262
ENO1
PFKL_775
EIF4EBP1
CAMK2D
HSPA8
EIF4EBP1_69;100
BRAF
HSP90AA1_263
HSP90AA1_263
HSP90AA1_263
HSP90AA1
BNIP3L
APEX1
HSP90AA1
HSP90AA1_231
HSP90AA1_231
EIF4EBP1_69
PKM_37
CAMK2D
LRP1_4461
NAA10
VIM
EIF4EBP1
PRKCA
RPS6_148
PPP3CA_469
HSP90AA1_231
GFPT1
CAMK2D
CAMK2G
PLCG1
CAMK2D
PRKD3
VIM.2
CDKN1A_78
NAA10
PRKCD
AKT1.1
AKT3
APEX1_18
JUN
BNIP3L_116;117;119
RAF1_295;296;301
ETS1_251
RAF1_296
VIM_361
KDM1A
KDM1A
FN1_2475
RRAS2
FN1_2475
HSPA5
IGF2
PRKD3_41;44
MMP14_470
IGF2
STUB1
EP300
PIK3C2A_261
JUN_73
BNIP3
UnT
6hE
24hE
C2C12 myotube acetylomics
Vim_168
Hspa9_159
Hspa9_291
Vim_445
Tpi1_56
Gapdh_192
Crebbp_1596
Hspa8_
Crebbp_1217
Pgk1_191
Crebbp_1593
Pgk1_361
Hspa9_567
Crebbp_1798
Gapdh_252
UnT
6hE
24hE
Mouse skeletal muscle proteomics
TF
PF
EF
B.
HIF1A signaling
1
0
−1
Eloc
UnT
6h EtOH
HIF1A Signaling
1.5
0
−1.5
Rps6
Vim
Rras2
Chp1
Elob
UnT
24hE
HIF1A targets
1
0
−1
Vim
UnT
24hE
Senescence
1.5
0
−1.5
Opa1
Rsl1d1
S100a6
Prpf19
Mdh1
Mif
Eif5a
Ckb
Rad21
UnT
6hE
MitoCarta3.0
1.5
0
−1.5
Etfb
Timm8a1
Timm50
Aldh9a1
Ahcyl1
Nln
Grhpr
Park7
Paics
Kars
Ndufa9
Cpt2
Atp5o
Mrps28
Clpx
Dap3
Opa1
Scp2
Bax
Dhrs1
UnT
6hE

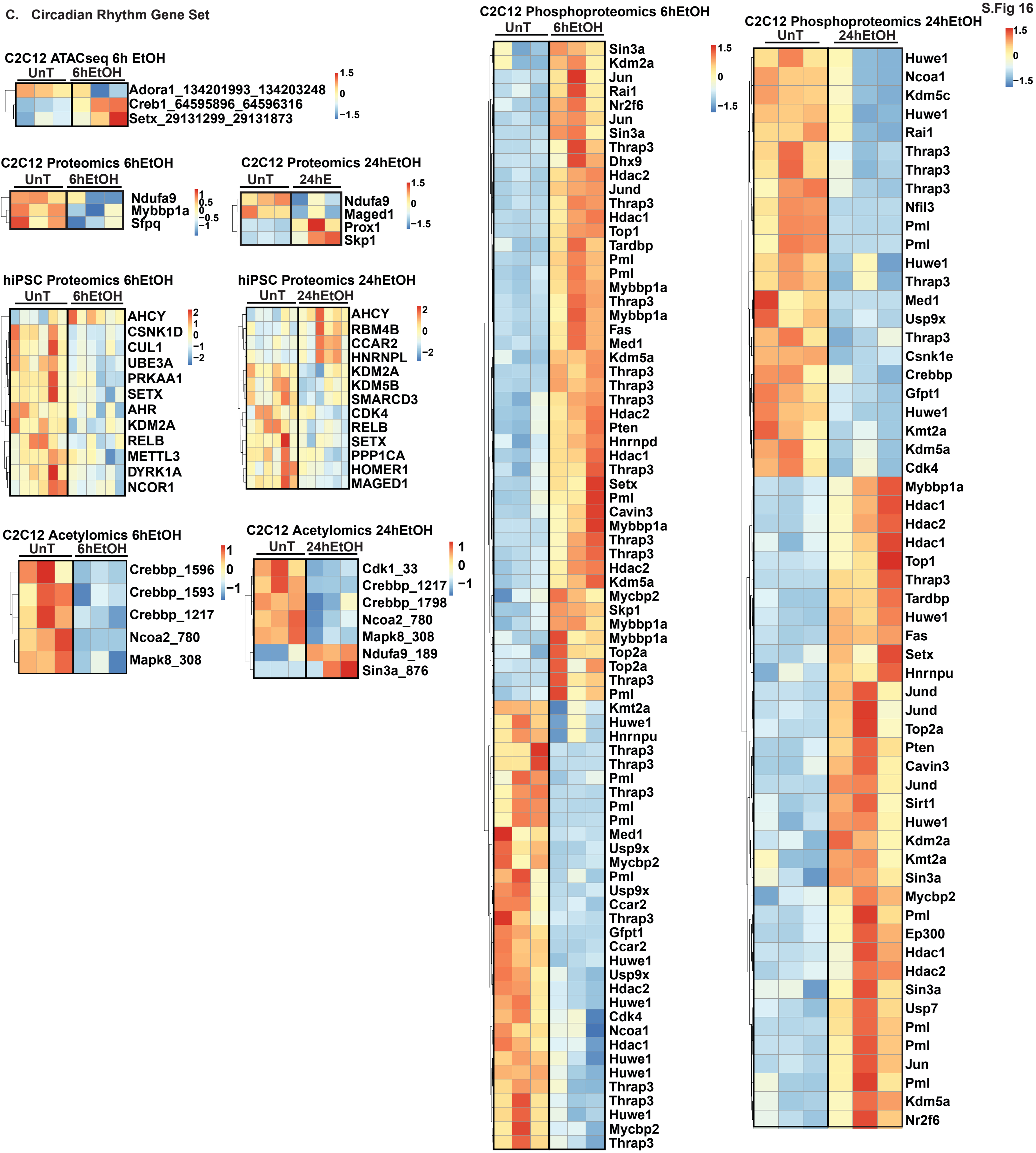
S.Fig 16
C. Circadian Rhythm Gene Set
C2C12 ATACseq 6h EtOH
UnT 6hEtOH
1.5 0 −1.5
Adora1_134201993_134203248
Creb1_64595896_64596316
Setx_29131299_29131873
C2C12 Proteomics 6hEtOH
UnT 6hEtOH
1 0.5 0 −0.5 −1
Ndufa9
Mybbp1a
Sfpq
C2C12 Proteomics 24hEtOH
UnT 24hE
1.5 0 −1.5
Ndufa9
Maged1
Prox1
Skp1
hiPSC Proteomics 6hEtOH
UnT 6hEtOH
2 1 0 −1 −2
AHCY
CSNK1D
CUL1
UBE3A
PRKAA1
SETX
AHR
KDM2A
RELB
METTL3
DYRK1A
NCOR1
hiPSC Proteomics 24hEtOH
UnT 24hEtOH
2 0 −2
AHCY
RBM4B
CCAR2
HNRNPL
KDM2A
KDM5B
SMARCD3
CDK4
RELB
SETX
PPP1CA
HOMER1
MAGED1
C2C12 Acetylomics 6hEtOH
UnT 6hEtOH
1 0 −1
Crebbp_1596
Crebbp_1593
Crebbp_1217
Ncoa2_780
Mapk8_308
C2C12 Acetylomics 24hEtOH
UnT 24hEtOH
1 0 −1
Cdk1_33
Crebbp_1217
Crebbp_1798
Ncoa2_780
Mapk8_308
Ndufa9_189
Sin3a_876
C2C12 Phosphoproteomics 6hEtOH
UnT 6hEtOH
1.5 0 −1.5
Sin3a
Kdm2a
Jun
Rai1
Nr2f6
Jun
Sin3a
Thrap3
Dhx9
Hdac2
Jund
Thrap3
Hdac1
Top1
Tardbp
Pml
Pml
Mybbp1a
Thrap3
Mybbp1a
Fas
Med1
Kdm5a
Thrap3
Thrap3
Thrap3
Hdac2
Pten
Hnrnpd
Hdac1
Thrap3
Setx
Pml
Cavin3
Mybbp1a
Thrap3
Thrap3
Hdac2
Kdm5a
Mycbp2
Skp1
Mybbp1a
Mybbp1a
Top2a
Top2a
Thrap3
Pml
Kmt2a
Huwe1
Hnrnpu
Thrap3
Thrap3
Pml
Thrap3
Pml
Pml
Med1
Usp9x
Mycbp2
Pml
Usp9x
Ccar2
Thrap3
Gfpt1
Ccar2
Huwe1
Usp9x
Hdac2
Huwe1
Cdk4
Ncoa1
Hdac1
Huwe1
Huwe1
Thrap3
Thrap3
Huwe1
Mycbp2
Thrap3
C2C12 Phosphoproteomics 24hEtOH
UnT 24hEtOH
1.5 0 −1.5
Huwe1
Ncoa1
Kdm5c
Huwe1
Rai1
Thrap3
Thrap3
Thrap3
Nfil3
Pml
Pml
Huwe1
Thrap3
Med1
Usp9x
Thrap3
Csnk1e
Crebbp
Gfpt1
Huwe1
Kmt2a
Kdm5a
Cdk4
Mybbp1a
Hdac1
Hdac2
Hdac1
Top1
Thrap3
Tardbp
Huwe1
Fas
Setx
Hnrnpu
Jund
Jund
Top2a
Pten
Cavin3
Jund
Sirt1
Huwe1
Kdm2a
Kmt2a
Sin3a
Mycbp2
Pml
Ep300
Hdac1
Hdac2
Sin3a
Usp7
Pml
Pml
Jun
Pml
Kdm5a
Nr2f6

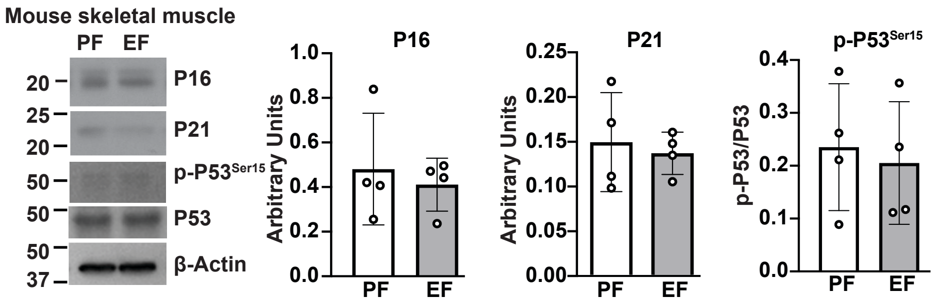
Mouse skeletal muscle
PF EF
20
25
20
50
50
50
37
P16
P21
p-P53Ser15
P53
β-Actin
P16
Arbitrary Units
1.0
0.8
0.6
0.4
0.2
0.0
PF EF
P21
Arbitrary Units
0.25
0.20
0.15
0.10
0.05
0.00
PF EF
p-P53Ser15
p-P53/P53
0.4
0.3
0.2
0.1
0.0
PF EF
